# A graph neural network-based interpretable framework reveals a novel DNA fragility–associated chromatin structural unit

**DOI:** 10.1186/s13059-023-02916-x

**Published:** 2023-04-24

**Authors:** Yu Sun, Xiang Xu, Lin Lin, Kang Xu, Yang Zheng, Chao Ren, Huan Tao, Xu Wang, Huan Zhao, Weiwei Tu, Xuemei Bai, Junting Wang, Qiya Huang, Yaru Li, Hebing Chen, Hao Li, Xiaochen Bo

**Affiliations:** 1 Institute of Health Service and Transfusion Medicine, Beijing, 100850 China; 2 4Paradigm Inc, Beijing, China; 3 The First Affiliated Hospital of Harbin Medical University, Harbin, 150001 China; 4 State Key Laboratory of Cardiovascular Disease, Fuwai Hospital, National Center for Cardiovascular Diseases, Chinese Academy of Medical Sciences and Peking Union Medical College, Beijing, China

**Keywords:** DSB, 3D chromatin structure, Interpretability, Graph neural network

## Abstract

**Background:**

DNA double-strand breaks (DSBs) are among the most deleterious DNA lesions, and they can cause cancer if improperly repaired. Recent chromosome conformation capture techniques, such as Hi-C, have enabled the identification of relationships between the 3D chromatin structure and DSBs, but little is known about how to explain these relationships, especially from global contact maps, or their contributions to DSB formation.

**Results:**

Here, we propose a framework that integrates graph neural network (GNN) to unravel the relationship between 3D chromatin structure and DSBs using an advanced interpretable technique GNNExplainer. We identify a new chromatin structural unit named the DNA fragility–associated chromatin interaction network (FaCIN). FaCIN is a bottleneck-like structure, and it helps to reveal a universal form of how the fragility of a piece of DNA might be affected by the whole genome through chromatin interactions. Moreover, we demonstrate that neck interactions in FaCIN can serve as chromatin structural determinants of DSB formation.

**Conclusions:**

Our study provides a more systematic and refined view enabling a better understanding of the mechanisms of DSB formation under the context of the 3D genome.

**Supplementary Information:**

The online version contains supplementary material available at 10.1186/s13059-023-02916-x.

## Background

DNA double-strand breaks (DSBs) are the DNA lesions most harmful to genome integrity that occur during transcription, DNA replication, and genotoxic agent exposure [1, 2]. Such DNA damage leads to genetic instability, which in turn may enhance the rate of cancer development [3]. A single DSB can be sufficient to kill a cell if it inactivates an essential gene or, in metazoa, triggers apoptosis [4]. DSBs that are left unrepaired may cause extensive loss of genetic information [5]. Faulty repair of DSBs can also lead to mutations or gross chromosomal rearrangements, which are hallmarks of cancer cells [2, 6].

The development of high-throughput sequencing techniques, such as DSBCapture [7], BLESS [8] and GUIDE-seq [9], has enabled the genome-wide mapping of DSBs. Based on these techniques, DSBs have been revealed to exhibit genomic preference. For example, DSBs occur preferentially at the TEAD motif (ATTCC/GGAAT) [10]; at regulatory elements, including promoters and active enhancers [11–13]; and at accessible DNA, hinted by epigenetic marks such as H3K4me1/2/3 [7, 14]. However, apart from genomic preference, DSBs also show a widely dispersed distribution across the genome [15] and neither the H3K4me3 mark nor proximal promoter activity is essential for the formation of DSBs [16–18]. Besides, topological stress mediated by transcription or replication as well as periodically spaced DNA bending also plays a positive role in DSB formation. Previous works focus on different aspects such as transcription, phase separation, DSB repair system, and DNA mobility in the context of DNA damage [19–22]. Though considerable progress has been made, these works provide scattered knowledge and sometimes produce ideas contradicting each other [23]. Therefore, which determinants other than above scattered factors define DSB target sites remains far from resolved.

Integrating 3D genome is a promising solution as it can organize those aspects into a systematic view, and it is fundamental enough to provide a settlement for various DSB-related events. Recent advances in chromosome conformation capture technologies, such as Hi-C [24] and ChIA-PET [25], have uncovered the relationships between DSBs and the 3D chromatin structure. For example, loop anchors serve as fragile sites that generate DSBs [26, 27], and the ordered topology of DSB-flanking chromatin may function as a barrier to enzymes whose uncontrolled activity could cause collateral DNA and/or chromatin damage [28]. Although ongoing efforts have been made, several limitations remain. First, most studies have focused on topologically associating domains (TADs) and chromatin loops. However, the large scale (hundreds of kilobases to megabase) of TADs does not allow for sufficient examination of DSB formation and corresponding transcriptional regulation, which commonly occur at the kilobase scale [8, 29]. Chromatin loops represent only a small subset of enriched features on Hi-C contact maps [30–32]. Thus, from a more refined and global view, the contributions of all features appearing on Hi-C contact maps to DSBs remain unclear. Second, detecting parts of complete Hi-C contact maps associated with DNA fragility is challenging. A recently developed machine learning–based approach for DSB prediction identified chromatin accessibility and long-range interactions as the best predictors [14]. That study demonstrated the ability of the computational approach to explore DSB-related factors, but the approach currently cannot capture the 3D genome information from Hi-C contact maps. Third, little attention has been given to the spatially organized determinants of DSB formation [33].

To solve the above limitations, we focused on the genome-wide Hi-C contact maps and designed a framework with GNN integrated as well as the advanced interpretable technique. We identified a novel and more refined chromatin structural unit, named the DNA fragility–associated chromatin interaction network (FaCIN). We demonstrated that FaCIN is a bottleneck-like structure and it enables the identification of candidate chromatin structural determinants. In brief, FaCIN reveals a universal form of how the fragility of a piece of DNA might be affected by the whole genome and helps to dissect the mechanisms driving DSBs under the context of 3D genome.

## Results

### GNN-based interpretable framework uncovers the relationship between 3D chromatin structure and DSB

**Fig. 1:**
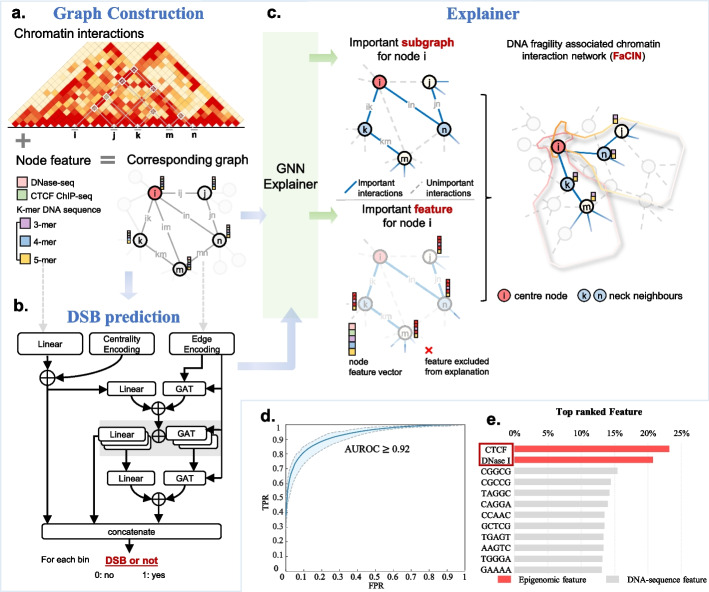
Overview of the framework to explain the relationship between the 3D chromatin structure and DSBs. **a** Input Hi-C contact map for each chromosome is transformed into an undirected graph where nodes represent genome bins and edges represent interactions. Node feature is a vector concatenated by three kinds of sub-features including: *k*-mer frequencies for DNA sequence, the CTCF ChIP-seq signals, and DNase I signals. **b** Architecture of DSB-GNN. The input graph first passes through a layer of two branches to encode edges and nodes, then goes through stacked layers of linear and GAT transformation. The node embeddings and edge embeddings are concatenated to finally produce the probability of a genome bin developing DSBs. **c** Identification of FaCIN via GNNExplainer. A node’s FaCIN is defined by its most important edges (i.e. chromatin interactions) and node features according to GNNExplainer’s explanation. **d** Receiver operating characteristic curve of DSB-GNN. Blue line reports the ROC curve averaged across all chromosomes. **e** CTCF and DNase I signals are the most important node features

To explain relationships between the 3D chromatin structure and DSBs, we first constructed a DSB prediction model and then generated interpretable explanations for predictions. In detail, we converted each chromosome into an undirected weighted graph G ∈ {*V, E*} (Fig. 1a), where *V* nodes represent 5-kb genome bins and *E* edges represent Hi-C contacts between nodes. Node feature derives from the *k*-mer (*K* = 3, 4, 5) DNA sequence, CTCF, and DNase I signals. Then, we built a graph neural network (GNN)-based DSB prediction model (DSB-GNN). DSB-GNN consists of mainly three graph attention convolution layers (GAT) with jump knowledge structure as well as encoding strategies for both node and edge (Fig. 1b). More details about the model architecture can be found in “Methods”.

We applied our approach to a normal human epidermal keratinocyte (NHEK) cell line and achieved high prediction accuracy [average area under the receiver operating characteristic curve (AUC) > 0.92 for each chromosome; Fig. 1d and Additional file 1: Fig. S1a]. We assessed the robustness of our model to variations in Hi-C read depth, bin size, and normalization (Additional file 2: Table S1). Results showed that the predictive performance exhibited a moderate decrease (AUC from 0.9251 to 0.8611 on raw data) along with down-sampled read depth from 100 to 20%. But it was not affected by different Hi-C normalization methods. While for resolution, with sufficient read depth, low resolution slightly reduced the performance but it tended to in turn bring elevation for particularly insufficient read depth. This was natural since high resolution used in low read depth would introduce much noise and sparsity. We performed ablation experiments and results showed that all components from both: (i) the model architecture such as self-attention mechanism and jumping knowledge structure, and (ii) the data we used such as Hi-C contact map as well as the node feature of *k*-mer, CTCF, and DNase I signals, they all make a due contribution (Additional file 2: Table S2). We benchmarked DSB-GNN with classical deep learning framework LightGBM [34] and machine learning framework Random Forest [35]. DSB-GNN overperformed both two methods (Additional file 2: Table S3). We also compared DSB-GNN with another method dedicated to DSB prediction [14, 36] (see “Methods” for comparison details). Compared to the method of Mourad et al., DSB-GNN was more suitable for whole genome study where DSBs and non-DSBs are typically imbalanced (Additional file 1: Fig. S1b). Above results demonstrated the power of DSB-GNN to capture relationships between the 3D chromatin structure and DSBs, allowing for further exploration into the explanations of DNA fragility.

Furthermore, we adopted the model-agnostic approach GNNExplainer [37] to interpret the contributing factors to DNA fragility under the context of 3D genome. For each genome bin, GNNExplainer yields a subset of Hi-C contact maps that are most influential for prediction of whether the bin contains a DSB. These crucial Hi-C contact maps, together with their node features, jointly formed the FaCIN (Fig. 1c).

We next examined the node features in FaCINs for all genome bins and results showed that CTCF and DNase I signals are the most important prediction marks (Fig. 1e and Additional file 1: Fig. S2a), which is consistent with previous findings [14] except that we observed CTCF is slightly more important than DNase I signal, supported by that chromosome loop anchors bound by CTCF and cohesin have higher vulnerability to DSBs [26]. Besides, we found that 6 out of the top-10 important *k*-mer features have a 4-bp overlap with DSB-preferred sequence (Additional file 1: Fig. S2b; see “Methods”).

Taken together, a well matching is shown between above results and currently established biological facts, which indicates a trustworthy capability of GNNExplainer and also encourages us to further examine FaCIN on whole genome to gain insights into the mechanistic understanding of DNA fragility.

### Unusual bottleneck pattern of FaCIN

To investigate the structural pattern of FaCIN, we first referred to each genome bin of interest as prediction site. For each prediction site, its FaCIN is identified as a connected subgraph, consisting of no more than 10 interactions that ranked top in their influence on the prediction. Different from loop or TAD, FaCIN is identified as a universal form of chromatin structural unit associated with DNA fragility. Specific definition of FaCIN can be found in “Methods”.

**Fig. 2:**
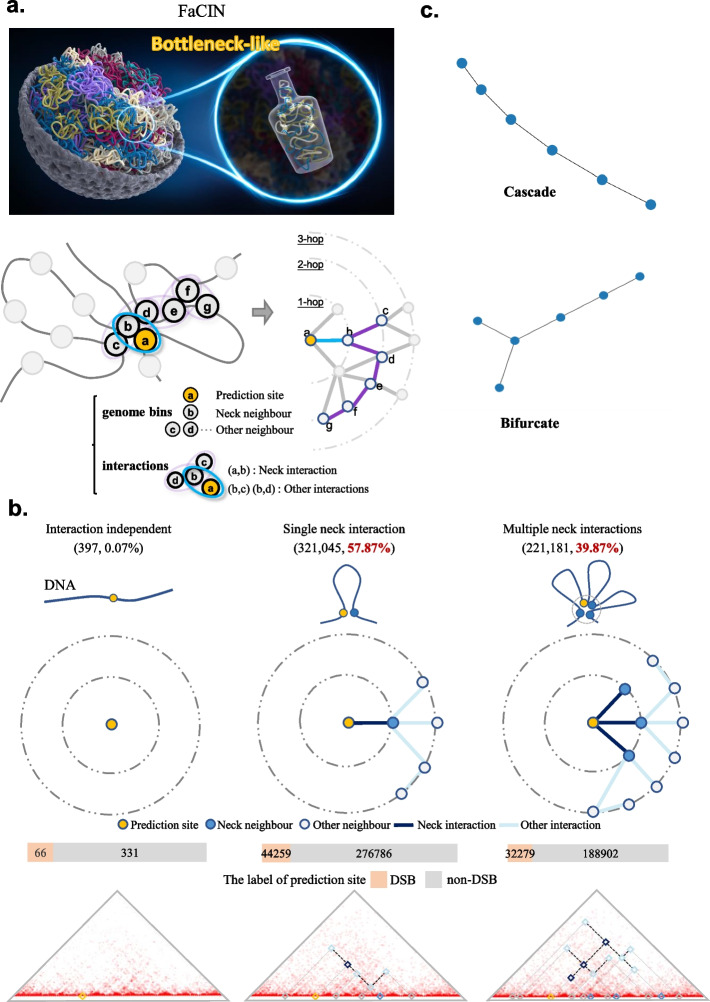
The bottleneck pattern of FaCIN. **a** Top: An overall schematic of FaCIN in the context of 3D chromatin structure. Tangled lines represent the intricate folding of chromatin. Bottom: Chromatin is binned with size of 5 kb and interacting bin pairs are abstracted into circles circled by ellipse. Each genome bin (ellipse) corresponds to a node (edge) on the right for a graphic form of FaCIN. Blue edge (ellipse) linking nodes a and b represents a neck interaction between the genome regions related to nodes a and b. Purple edges represent other interactions in FaCIN. **b** FaCINs with different neck interaction number and their corresponding reflection on Hi-C contact maps. Squares on Hi-C contact map are colored according to their role within a FaCIN. **c** Top-2 motifs that are enriched in FaCINs

Interestingly, we found FaCIN suggestive of a “bottleneck” (Fig. 2a). To figure out the bottleneck pattern, we performed the following calculations. First, according to Hi-C contact maps, each prediction site has on average 91 direct interactions, but only 1.6 of them are present in FaCINs (Additional file 1: Fig. S3). Specifically, prediction site that (i) with zero interactions (i.e., isolated) take less than 1% of FaCINs, (ii) with only one direct interaction account for a majority (nearly 58%) of FaCINs and (iii) with two or three direct interactions for around 40% of FaCINs (Fig. 2b). To sum up, in FaCIN, the prediction site typically contacts only one or two nodes while the latter subsequently contact far more nodes. Viewed from the prediction site, FaCIN’s interactions tend to form a shape going from narrow to wide that visually resembles a bottleneck. A schematic with more details of the bottleneck pattern can be found in Additional file 1: Fig. S4.

Second, we introduced the concept of betweenness centrality, a classic metric in graph theory, which indicates how often a node appears on the shortest path between any random node pairs. If a node has a higher value of betweenness centrality, it has a stronger mediating or bridging role as this metric actually. We calculated the betweenness centrality of 1-hop and 2-hop neighbors to see how often they appear on the shortest path between other nodes and prediction site (see details in “Methods”). We found that the 1-hop neighbors have on average a much higher betweenness centrality (Additional file 1: Fig. S5), meaning that 1-hop neighbors are in bottleneck positions that have more control over the information in FaCIN. Based on both visual resemblance and computational support, we identified the bottleneck pattern and renamed 1-hop neighbors as neck neighbors due to their special position. Subsequently, neck interactions, other neighbors, and other interactions are also determined (see more details in “Methods”).

The bottleneck pattern is expected to have potential biological role due not only to the above regularity, which suggests a general association between FaCIN with global properties of DNA fragility, but also to those explanations from GNNExplainer applied in some similarly complex tasks that have been proved justified [37]. For example, in molecular property prediction, patterns including “carbon ring” and some other chemical groups known from the prior knowledge are significantly enriched in GNNExplainer’s explanation for decision-making.

We further investigated the biological insights encoded in the bottleneck pattern. First, it indicates that the chromatin interactions associated with DNA fragility are organized, not randomly, but in a spatially ordered manner. To illustrate this point, we generated randomized graphs that have the same overall characteristics as the FaCIN (see details in “Methods”). Then we performed subgraph searching (see “Methods”) on both FaCINs and randomized graphs. We found that FaCINs showed enrichment of two motifs while randomized graphs failed (Fig. 2c, Additional file 1: Fig. S6). These two motifs exhibited “cascade” and “bifurcate” mode and, the existence of motif was in itself informative, indicating that FaCINs contain the universal building blocks concerning DNA fragility in terms of the chromatin interaction level.

Second, the bottleneck pattern shows that direct chromatin interactions are, counter-intuitively, not necessarily more influential to DNA fragility than those indirect. Otherwise, the prediction site should be surrounded by nearly all direct neighbors leading to the pattern shaped like a “cycle” (Additional file 1: Fig. S7), while results did not turn like that. This stresses that studying DSB under the view of 3D genome is quite necessary as some indirect interactions prevail over direct ones, which reveals a more complicated DSB mechanism than it appears. Besides, neck neighbors are not required to correspond to those regions that are most close to prediction site in 3D space with higher intensity of interaction. The 1D genomic length spanned by FaCIN’s interactions varies from kilobase to megabase (Additional file 1: Fig. S8 and Additional file 2: Table S4), indicating the DNA fragility–associated genome organization often involves long-range chromatin interactions.

In summary, FaCIN helps to reveal that the fragility of a piece of DNA is associated with other genomic regions in a cascading manner, that is, the prediction site directly communicates with neck neighbors and neck neighbors gather biological information from many more genome regions at distance.

### Characterization of neck interactions in the context of chromatin structure

**Fig. 3:**
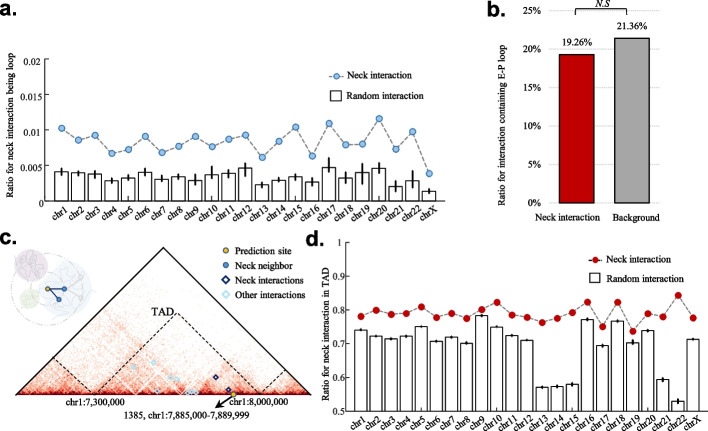
Characterization of neck interactions in the context of chromatin structure. **a** Ratio for neck interactions being loop is marked with blue dot, while the ratio for random interactions (10 repeats) is displayed with box. Neck interactions are significantly enriched in loop anchors (*p* < 0.001, hypergeometric test). **b** E-P loops show no enrichment of neck interactions. **c** Example for the FaCIN of DSB site at chr1: 7,885,000–7,889,999 reflected on Hi-C contact map. TADs are separated by black dashed lines. **d** Ratio for neck interactions that entirely locate in a TAD is marked with red dot, while for random interactions (10 repeats) this ratio is marked with box. Neck interactions are more likely to locate in a TAD than random interactions (*p* < 0.001, hypergeometric test)

To characterize neck interactions, we first investigated their relationships with well-known chromatin structural components, namely loops [31, 38] and TADs [39]. We obtained 19,632 chromatin loops and 2832 TADs from publicly available Hi-C data on the NHEK cell line [31]. A chromatin loop occurs when stretches of genomic sequence that lie on the same chromosome (configured in cis) are in significantly closer physical proximity to each other than to intervening sequences [31, 40]. We found that the neck interactions were significantly enriched in loop interactions (*p* < 0.001, hypergeometric test, Fig. 3a and Additional file 1: Fig. S9). E-P loops are fundamental controllers of cell-type-specific gene expression [38, 39] and are mediated by the structural regulator Yin Yang 1 (YY1) [41]. We detected E-P loops, and found no enrichment of neck interactions of FaCINs in them (Fig. 3b). TADs are domains with high frequencies of chromatin interaction [42] and serve as functional units for DNA damage response [43]. We found that nearly 80% of neck interactions are located within a single TAD; this proportion is significantly higher than that for all chromatin interactions (Fig. 3c, d). Besides, TAD boundaries also showed significant neck interaction enrichment (Additional file 1: Fig. S10). These results suggest that neck interactions are restricted by TADs.

The enrichment of neck interactions in loop anchors and TADs, as well as at TAD boundaries, shows that neck interactions help to explain how TADs and loops contribute to DSB formation.

### FaCIN allows for a settlement of DSB-related findings from different perspectives

We investigated how neck interactions are biologically distinct between DSB and non-DSB sites. The result showed that the average length of neck interactions at DSB sites (527.6 kbp, median 145 kbp) was comparable to that for non-DSB sites (Additional file 1: Fig. S11a). Similarly, the intensity of neck interactions at DSB and non-DSB sites was also comparable (Additional file 1: Fig. S11b). These results are supported by the previous finding that stabilization of chromatin topology safeguards genome integrity [28], suggesting the maintenance of the key 3D chromatin structure when a DSB occurs.

**Fig. 4:**
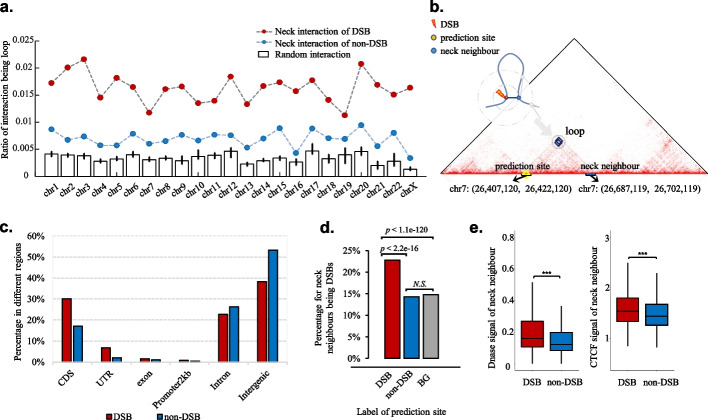
Neck interactions at DSB sites are biologically distinct from those at non-DSB sites. **a** Neck interactions at DSB sites (dashed line with red dot) are more likely to be loops than those at non-DSB sites (dashed line with blue dot). **b** Example that the neck interaction of DSB site is a loop, of which the left anchor is the DSB site and the right anchor is a neck neighbor of DSB site. **c** Neck neighbors of DSB sites are more enriched in region of CDS and UTR, compared with those of non-DSB sites. **d** Neck neighbors of DSB sites are more likely to be DSBs. **e** Neck neighbors of DSB sites are more enriched with CTCF and DNase I signals than those of non-DSBs

However, neck interactions at DSB sites were more enriched in loop anchors than were those at non-DSB sites (Fig. 4a). This finding is consistent with the vulnerability of loop anchors to DSBs [26, 27]. In addition, we identified 1242 loops with DSBs on one anchor and another anchor linked by neck interactions (Fig. 4b, Additional file 2: Table S5), and this number was significantly higher than that for random interactions (*p* < 0.001, hypergeometric test). Considering the importance of loop extrusion for the formation of DNA damage repair foci [43], these DSB-associated chromatin loops may be candidate sets for further experiments to study DSB formation.

We further examined neck neighbors and observed that most of the neck neighbors of DSB sites themselves were largely non-DSB (nearly 80%, Additional file 1: Fig. S12). It quite matches the knowledge as follows: DSBs are often introduced to release the torsional stress introduced by continued cell activities like transcription and replication. TOP2B, a main isoform of mammalian type II topoisomerases, will transiently break and rejoin DNA strands [26]. But at an unknown frequency, TOP2B can fail to rejoin the broken DNA strands, leading to the result that two physically interacting genomic regions turn to one having TOP2B-induced breaks and the other being intact.

Neck neighbors of DSB sites were significantly enriched in coding regions CDS (odds ratio = 2.12, *χ*^2^ = 12,077, *p*-value < 2.2e − 16) as well as UTR (odds ratio = 3.12, *χ*^2^ = 9106.8, *p*-value < 2.2e − 16), compared with those of non-DSB sites (Fig. 4c). Similarly, moreover, neck neighbors of DSB sites were more likely to be DSBs (Fig. 4d), suggesting a DSB clustering phenomenon. This is supported by that more closely positioned DSBs are more likely to interact [44]. As closely positioned DSBs cannot access the repairing factors hindered by a same chemical block or compact chromatin, these DSBs might be left unrepaired together. We further investigated the epigenome marks of neck neighbors and observed that DNase I and CTCF signals were significantly higher at neck neighbors of DSB sites than at those of non-DSB sites (Fig. 4e). CTCF enrichment and high chromatin accessibility at DSB neck neighbors help to explain DSB formation.

Above results show that 3D genome provides a more systemic view and the FaCIN found under such a view allows for a settlement of DSB-related key findings from varied perspectives and thus is rooted in a biologically reasonable ground.

### FaCIN provides new insights for DSB formation

**Fig. 5:**
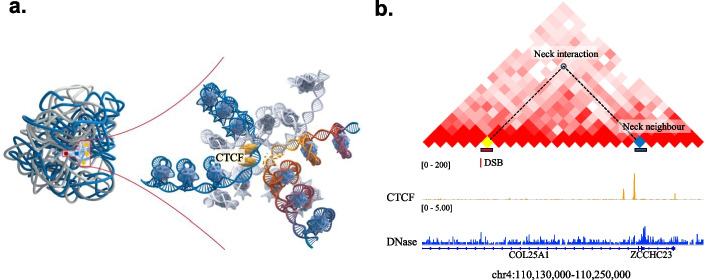
Interpretation for DSB formation model in the context of 3D chromatin structure. **a** DSB-surrounding space, rather than the cleavage site itself, may develop the DSB through its FaCIN with key chromatin interactions spatially organized like a bottleneck. **b** Example on Hi-C contact map to explain the model in **a.** The neck interaction connects a DSB site and its non-DSB neck neighbor. The well-established DSB signals of CTCF binding and accessible chromatin, however, do not show up at the cleavage site, but occur at its neck neighbor

Although progress has been made in precise mechanism of DSB formation and several works have offered well-established DSB marks such as CTCF binding and accessible chromatin [43, 45], some difficult questions remain unanswered. For example, why do some DSBs occur while exhibiting no known marks? Based on the above results, FaCIN provides new insights into DSB formation with respect to the 3D chromatin structure. In contrast to previous work [10, 14, 36], in which factors related to DSBs have been considered mainly from view of the linear genome (e.g. the sequence preference at the cleavage site or epigenetic information for the DSB-flanking region), FaCIN reveals how the DSB surrounded space, rather than the cleavage site, may influence DSB formation through one or several chains of chromatin interactions. These interactions correspond to key genomic loci that may determine DSBs through varied epigenetic signals, such as CTCF binding and chromatin accessibility (Fig. 5a). That is, the well-known DSB marks might be absent from DSB cleavage site but show up at their neck neighbors (Fig. 5b). The new insights suggest that to dissect DSB formation requires interpreting it in the context of 3D genome organization.

## Discussion

DSBs are major threats to cells, and the 3D chromatin structure provides an important template for DSB formation and repair [28]. To explain the relationships between DSB and 3D genome poses a challenge in modeling DNA fragility with chromatin structural information incorporated. Recent works such as the methods in Mourad et al. and Ballinger et al. have offered sparks by using DNA shape and Hi-C data respectively [14, 36]. Inspired by their works, we also use Hi-C data to incorporate 3D genome information, but consider more about the template role of 3D genome, that is, chromatin is the context in which DSB and its repair take place. In DSB-GNN, Hi-C contact map is converted into graph, of which the nodes and edges represent the genome regions and chromatin interactions. The graph provides a scaffold that naturally encodes rich information from genome as well as the chromatin organization. Compared to simply stacking Hi-C with other types of features, the way that DSB-GNN uses Hi-C data is very likely to be more reasonable and more promising to provide new insights as well.

The bottleneck pattern of FaCIN indicates that the fragility of a piece of DNA is associated with other genomic regions in a cascading manner, that is, the prediction site directly communicates with neck neighbors and neck neighbors gather biological information from many more genome regions at distance. Related analysis indicates that FaCIN is promising to coordinate DSB-associated phenomena observed under different conditions. FaCIN’s bottleneck pattern helps to identify candidate genomic loci and chromatin interactions as structural determinants for DSB formation. FaCIN’s size varies from kilobase to megabase, indicating the DNA fragility–associated genome organization might exceed the space of ordinary TAD level. This also helps us to understand long-range chromatin interactions from a new perspective.

With hundreds of publicly available Hi-C datasets accumulated [31, 32, 46–50], DSB-GNN can be employed to explore cell-type-specific structural units related to genome integrity. Besides, ideas from our work merit extension to more tasks, such as the identification of the chromatin structure associated with DSB repair.

Our GNN-based model showed a powerful ability to model relationship between chromatin structural information and DNA fragility, and hence provided possible direction to pursue a unifying perspective. In turn, such a process helps to reveal the current limitations in interpretable deep learning. In this work, we did not focus on only refining what is known but more on bridging the gaps from scattered perspectives. This work is likely to be a modest step to advance the iterative cycle of development in both interpretable deep learning and genomics.

## Conclusions

In summary, this study exemplifies the potential of the interpretable AI for DNA-damage-related researches. We develop a GNN-based framework DSB-Graph and demonstrate how the interpretations derived from the relationship between the 3D genome and DSB enable identification of a novel DNA fragility–associated chromatin structural unit. This unit is promising to present a unified view to coordinate different DSB-associated phenomena. Overall, this framework is applicable beyond DSB to other genomic events that have particularly intricate relationship with the 3D chromatin structure.

## Methods

### Data resource

The publicly available Hi-C data of NHEK cell line is obtained from Rao et al. [31], and we used the 5-kb resolution intrachromosomal contact maps. The DSB data identified by DSBCapture in NHEK cell line is available in the NCBI Gene Expression Omnibus with accession number GSE78172 [7]. The ChIP-seq data of CTCF and DNase-seq data for NHEK cells were retrieved from ENCODE project [51].

### Hi-C data preprocessing

We used the raw Hi-C data. Unlike most Hi-C related studies, our theme is distinct from those aiming to provide discoveries based on significant chromatin structural units. Therefore, several points here are different and need to be explained in detail.

First, we only used intrachromosomal data. The reasons for excluding inter-chromosomal interactions are two-fold. On the one hand, the computation will otherwise not be easily manageable. Taking all inter-chromosomal interactions into account means that the graph will expand to the genome scale and the number of nodes will substantially increase while posing a real challenge to computational time. On the other hand, DSBs are widely dispersed across whole genome while inter-chromosomal interactions are much sparser than intrachromosomal ones.

Second, we did not perform normalization to remove the noise or biases. For most Hi-C downstream analyses, such as loop identification, it is more suitable to use normalized data than using raw counts. One could not have identified reliable loops without removing the biases in Hi-C data, as the definition of loop directly depends on the quality of Hi-C data. However, for DSB-GNN, the ground truth (DSB or non-DSB label) derives from DSBCapture, which is another high-throughput sequencing technique unrelated to Hi-C experiment. Therefore, the noise and biases introduced by Hi-C data would not harm the reliability of DSB label. As the ground truth is fixed, whether a model is affected by the noise or biases of input will manifest in its performance, for example, the performance of DSB-GNN will drop if the data noise or biases cannot be overcome. We tested DSB-GNN with both KR- and ICE-normalized data and their performances are close to that of using raw counts (see “Methods” section for robustness evaluation). In addition, current normalization methods make assumptions that certain factors in Hi-C experiments are responsible for the biases or that the biases are scalar, multiplicative, one-dimensional, and so on. But whether these assumptions are applicable to the context of DSB is actually unknown.

Third, we did not distinguish distal interactions from those proximal. As the significance of an interaction might be distorted by linear proximity, this step is often required for most Hi-C related studies. While in DSB-GNN, FaCIN is identified as a universal form in terms of chromatin interactions, which exactly requires the raw data as global as possible and therefore should not exclude interactions according to significance.

Finally, to identify FaCIN from the raw counts as globally as possible, we tried to reserve almost every non-zero contact as an interaction. The merit of doing this is that we would not miss the patterns that are functionally important but not statistically significant. To determine the threshold under which a contact can be discarded without affecting performance, we calculated the distribution of raw contact counts (Additional file 1: Fig. S13a) and tested different thresholds. Results showed that when we require the raw contact counts to be at least 2, the performance and the computational efficiency are both satisfactory (Additional file 1: Fig. S13b and Additional file 2: Table S6).

### Graph representation

We model each chromosome as an undirected graph and there are in total 23 graphs since we do not consider Y chromosome. Each graph can be formally defined as G ∈ {V, E}, where node v_i_ ∈ V represents a 5-kb genome bin, and edge e_i_ ∈ E represents the interaction between corresponding genome bins.

Both edges and nodes have attributes reflecting the associated properties, such as the following:

1. Edge weight is the count of corresponding entry in raw observed contact maps.
2. Node type can be either DSB or non-DSB, according to whether or not there is DNA double-strand break in this node’s genome region.
3. Node feature is a vector of length 1346 concatenated by three kinds sub-features including (i) the *k*-mer frequencies for DNA sequence (of length 4^3^ + 4^4^ + 4^5^ = 1344 for *K* = 3, 4 and 5), (ii) the CTCF ChIP-seq peak density, and (iii) the DNase-seq peak density for the 5-kb region of this node. These node features are selected according to previous studies [14, 36].

### DSB prediction model

Here we introduce the overall framework of the three-layer DSB-GNN model. Regarding the choice of number of layers, we trained different models with number of GAT layers ranging from 1 to 5 and 3-GAT layer model achieves the highest AUROC (Additional file 2: Table S7). Like many other GNN-based frameworks, our model consists of two steps: aggregation of neighbor message and update of node representation. Specifically, for node ${v}_{i}$, let its feature vector be ${x}_{i}$ and its representation in the *l*th layer be ${h}_{i}^{(l)}$ and ${h}_{i}^{\left(0\right)}$=${x}_{i}.$ The computation of neighbor aggregation (AGG function) and update (UPDATE function) in the *l*th layer is as follows:

1 $${m}_{i}^{(l)}={\mathrm{AGG}}^{\left(l\right)}\left(\left\{{h}_{i}^{\left(l-1\right)}:j\in N\left({v}_{i}\right)\right\}\right)$$

2 $${h}_{i}^{(l)}={\mathrm{UPDATE}}^{\left(l\right)}\left({h}_{i}^{\left(l-1\right)},{m}_{i}^{\left(l\right)}\right)$$

where ${m}_{i}^{(l)}$ represents the message aggregated from the neighbors of ${v}_{i}$ in the *l*th layer, and $N\left({v}_{i}\right)$ is the set of *one-hop* neighbors of ${v}_{i}$. The term *hop* is used to determine the neighborhood radius. For example, given the node ${v}_{i}$, its *one-hop* neighbors are those nodes that have direct connection with ${v}_{i}$; its *two-hop* neighbors are those nodes that have no connection with ${v}_{i}$ but with ${v}_{i}$’s *one-hop* neighbors.

To build a more effective model, we introduced the *self-attention* mechanism proposed by graph attention networks (GAT) [52]. In original GAT, the attention coefficient used in AGG function is expressed as:

3 $${e}_{ij}=\beta \left(W{h}_{i}, W{h}_{j}\right), {\alpha }_{ij}={\mathrm{softmax}}_{j}\left({e}_{ij}\right)=\frac{\mathrm{exp}({e}_{ij})}{{\sum }_{k\in N({v}_{i})}\mathrm{exp}({e}_{ik})}$$

where ${e}_{ij}$ is the attention coefficient that indicates the importance of ${v}_{j}$’s features to ${v}_{i}$, $\beta$ is a learnable weight vector, and ${\alpha }_{ij}$ is the result of ${e}_{ij}$ after softmax function to facilitate the comparison of different nodes’ coefficients. This original version of attention coefficient in Eq. 3 only considers node feature but neglects edge feature. However, in the context of DSB prediction, the edge feature contains very important structural information of 3D chromatin organization. Therefore, we added edge feature into the calculation of attention coefficient as follows:

4 $${\alpha }_{ij}=\frac{\mathrm{exp}(\mathrm{LeakyReLU}({\beta }^{T}[{W}_{N}{h}_{i}||{W}_{N}{h}_{j}||{W}_{E}{h}_{(i,j)}]))}{{\sum }_{k\in N({v}_{i})}\mathrm{exp}(\mathrm{LeakyReLU}({\beta }^{T}[{W}_{N}{h}_{i}||{W}_{N}{h}_{k}||{W}_{E}{h}_{(i,k)}]))}$$

where $\beta$ is a learnable weight vector, ${W}_{N}$ and ${W}_{E}$ denote two trainable weight matrices, and || denotes the concatenation operation. The edge feature ${h}_{(i,j)}$ is obtained from the interaction strength ${e}_{(i,j)}$ between two genome bins through a linear layer, which is defined as *edge encoding*. The purpose of *edge encoding* is to keep the edge feature and the node feature dimension consistent. Finally, the neighbor aggregation and update of node representation are formulated as:

5 $${h}_{i}^{(l)}=\sigma ({\sum }_{j\in N({v}_{i})}{\alpha }_{ij}^{(l-1)}{W}^{(l-1)}{{h}_{j}}^{(l-1)})$$

where $\sigma$ represents nonlinear transformation.

To further improve the expressive ability of the model, we introduced the jumping knowledge (JK) architecture [53], defined a *centrality encoding* to consider the node degrees following Graphormer [54] and we also introduced the *positional encoding* in Transformer [55] as a supplement to node features to add sequential information of chromatin (use the ID of the genome bin as position). After adding the *centrality encoding* and the *positional encoding* to the input, the model can capture both the semantic correlation and the node importance via attention mechanism.

### Implementation details

We took one chromosome as a hold out for test and the remaining chromosomes were used for training (21 chromosomes) and validation (one chromosome). We trained the DSB prediction model on training set and evaluated it on the test set. We performed this process 23 times and each chromosome was given the opportunity to be used as test set for one time. Cross entropy is used as the loss function. All models were implemented with Pytorch (version 1.7.0) [56] on a GPU 2080 Ti. In addition, in order to facilitate the GNN implementation, we used the popular GNN library of Deep Graph Library (DGL) of version 0.6.0 [57].

### Ablation experiments

To provide the contributions of all components of DSB-GNN, we performed complete ablation experiments on both input features and structural designs. Details are as below:

(i) For node feature, we remove each sub-feature at a time.
(ii) For Hi-C information, simply removing Hi-C network is not applicable as the graph constructed from Hi-C is a necessary input and if the graph does not exist, the whole GNN-base model could no longer work. We replaced the contact counts in adjacency matrix (computational representation of Hi-C graph) with all zeros and kept all other elements unchanged.
(iii) For components of the model design, to test the contribution of self-attention strategy, we compared DSB-GNN with a GCN-based model which has almost a same structure except without self-attention mechanism. Besides, we also tested the contributions of other components including edge coding, centrality encoding, positional encoding, and Jumping Knowledge (JK) structure.

Results of above ablation experiments (Additional file 2: Table S2) showed that the self-attention strategy, the JK structure, the use of *k*-mer, CTCF, DNase-seq, and Hi-C data each makes a due contribution.

### Robustness evaluation

In reality, different experimental settings might produce Hi-C data with varied read depth and subsequently affect the selection for optimal bin size. Therefore, in most Hi-C related studies, the selection of the resolution should match the read depth to avoid introducing too much noise and sparsity. This match is necessary for downstream analysis such as calling loops or TADs which involves identifying interactions with significantly higher contacts. These factors together with different normalization approaches all pose a challenge to the robustness of a model that leverages Hi-C data.

Here, in our study, unlike loops or TADs, FaCIN focuses on revealing a universal form in terms of chromatin interaction and therefore does not require filtering significant interactions. In contrast, what FaCIN requires is exactly the raw data as global as possible. Despite this, it is still important to evaluate our model’s robustness against parameters like different Hi-C read depth, bin size, and normalization approaches.

Specifically, we first down-sampled the NHEK Hi-C dataset into 4 subsets ranging in size from 20 to 100% of the initial sequencing depth. For example, compared to the initial data, a 20% set had around a fifth of the total contacts while being restricted to be subject to a same distribution. Each of the subset was then retained (raw) or normalized with Knight-Ruiz (KR) and ICE approaches [58, 59]. Then the datasets were further binned with different sizes of 5-kb, 10-kb, and 25-kb. We tested DSB-GNN on these datasets and found that:

1. The predictive performance exhibited a moderate decrease (AUC from 0.9251 to 0.8611 on raw data) along with down-sampled Hi-C read depth from 100 to 20%, indicating the robustness of our model against data read depth.
2. The predictive performance was not affected by different Hi-C normalization methods. This is not surprising because in our model the normalization is no longer a preprocessing step but partly transferred to the GNN model.
3. While for resolution, with sufficient read depth, low resolution slightly reduced the performance but it tended to in turn bring elevation for particularly insufficient read depth. This was natural since high resolution used in low read depth would introduce much noise and sparsity.

To sum up, the 5-kb bin size is used as a window to examine DSBs instead of to discern new loops or TADs. Despite that noise and sparsity problems might exist, they are solved actually within DSB-GNN. Besides, the comparative performances for different normalization approaches also indicate that normalization is no longer a preprocessing step but partly transferred to DSB-GNN. Above results are provided in Additional file 2: Table S1.

### Method comparison

We compared DSB-GNN with LightGBM [34] and Random Forest (RF) [35] (two widely used methods to benchmark deep learning works). We also performed ablation experiments with subsets of features on them. Results showed that DSB-GNN consistently outperformed LightGBM and RF across different subsets of features, and integrated features brought a boosted performance for all three methods. Above information is provided in Additional file 2: Table S2.

Furthermore, we compared DSB-GNN with a method proposed by Mourad et al. which is specialized for DSB studies [14, 36]. This method also takes DSB prediction as a binary classification. We first reproduced this method totally following the instructions in the paper. Reproduced results on NHEK dataset (AUC = 0.9678) were close to their reported performance (AUC = 0.970). As described in Mourad et al., by choosing a same number of non-DSB sites with genomic sequences that well match in sizes, GC, and repeat contents of DSB sites, their data set is constructed as a class-balanced one where DSB vs non-DSB is 1:1. However, this is seldom the real case, where non-DSB should largely outnumber DSB, for example, for the NHEK dataset we used where the DSB sites are mapped by DSBCapture high-throughput sequencing, the ratio of DSB to non-DSB on whole genome is roughly 1:6. We used this imbalanced dataset to compare DSB-GNN and the method in Mourad et al., and results showed that DSB-GNN performed better in a class-imbalanced situation while the method in Mourad et al. was more advanced in discriminating DSB-related details from others trivial.

Another method proposed by Ballinger et al. treats DSB prediction as a regression problem. It estimates the DSB frequency per 50-kb region along the whole genome with a random forest regression model. The authors evaluated their model using Pearson’s correlation between predicted and observed DSB frequency. Due to the differences in task property, a direct comparison might not perfectly suit here so we did not include this method into comparison.

### General principle of GNNExplainer for explanation

GNNExplainer is the first model-agnostic approach to provide interpretable explanations for predictions made by any GNN-based model [37]. We next introduce its general principle for explanation.

(i) Denotations. For node ***v***, let us denote that computation graph by ***G***_***c***_(***v***), the associated binary adjacency matrix by ***A***_***c***_(***v***) ∈ {0, 1}^*n*×*n*^, and the associated feature set by*** X***_***c***_(***v***) = {*x*_*j*_ |*v*_*j*_ ∈ ***G***_***c***_(***v***)}. The GNN model *Φ* learns a conditional distribution ***P***_*Φ*_ = {***Y***|***G***_***c***_, ***X***_***c***_}, where ***Y*** is a random variable representing labels {**1**,..,***C***}, indicating the probability of nodes belonging to each of ***C*** classes. A GNN’s prediction is given by $\widehat{{\varvec{y}}}$** =** *Φ*(***G***_***c***_(***v***)***, X***_***c***_(***v***)), meaning that it is fully determined by the model *Φ*, graph structural information ***G***_***c***_(***v***), and node feature information ***X***_***c***_(***v***). In effect, this implies that we only need to consider graph structure ***G***_***c***_(***v***) and node features ***X***_***c***_(***v***) to explain $\widehat{{\varvec{y}}}$. Formally, GNNExplainer generates explanation for prediction $\widehat{{\varvec{y}}}$ for node ***v*** as (***G***_***s***_(***v***), ***X***_***s***_(***v***)), where ***G***_***s***_(***v***) is a small subgraph of the computation graph ***G***_***c***_(***v***) and ***X***_***s***_(***v***) is a small subset of node features that are most important for explaining $\widehat{{\varvec{y}}}$.

(ii) Problem Formulation. GNNExplainer formalizes the notion of “most important” using mutual information ***MI*** and it turns out to be the following optimization framework:

6 $$\underset{{G}_{S}}{\mathrm{max}}MI \left(Y, \left({G}_{S}, {X}_{S}\right)\right)=H\left(Y\right)-H(Y|G={G}_{S}, X={X}_{S})$$

For node ***v***, ***MI*** quantifies the change in the probability of prediction $\widehat{{\varvec{y}}}$** =** *Φ*(***G***_***c***_(***v***)***, X***_***c***_(***v***)) when ***v***’s computation graph is limited to explanation subgraph ***G***_***s***_(***v***) and its node features are limited to ***X***_***s***_(***v***).

(iii) Calculation. Examining Eq. (6), we see that the entropy term *H*(*Y*) is constant because model *Φ* is fixed for a trained GNN. As a result, maximizing mutual information between the predicted label distribution ***Y*** and explanation (***G***_***s,***_*** X***_***s***_) is equivalent to minimizing conditional entropy term *H*(*Y* | *G* = ***G***_***s,***_* X* = ***X***_***s***_), which can be expressed as follows:

7 $$H\left(Y|G={G}_{S}, X={X}_{S}\right)= -{\mathbb{E}}_{Y|{G}_{S},{X}_{S}}[log {P}_{\Phi }(Y|G={G}_{S}, X={X}_{S})]$$

Explanation for prediction $\widehat{{\varvec{y}}}$ is thus a subgraph ***G***_***s***_ that minimizes uncertainty of *Φ* when the GNN computation is limited to ***G***_***s***_. In effect, ***G***_***s***_ maximizes probability of$\widehat{{\varvec{y}}}$. By imposing a constraint on ***G***_***s***_’s edge number as: |*** G***_***s***_ |≤ ***K***_***m***_, ***G***_***s***_ has at most*** K***_***m***_ edges. In effect, this implies that GNNExplainer generates ***G***_***s***_ by taking ***K***_***m***_ edges that give the highest mutual information with the prediction.

If we treat ***G***_***s***_∼$\mathcal{G}$ as a random graph variable and plus the Jensen’s inequality, the objective in Eq. 7 can be eventually transformed into:

8 $$\underset{\mathcal{G}}{\mathit{min}}\;H\left(Y|G={\mathbb{E}}_{\mathcal{G}}\left[{G}_{s}\right], X={X}_{s}\right)$$

where the ${\mathbb{E}}_{\mathcal{G}}\left[{G}_{s}\right]$ can be implemented by a masking of the computation graph of adjacency matrix, ${A}_{c}\odot \sigma (M)$, where ${A}_{c}$ denotes the associated binary adjacency matrix of computation graph ***G***_***c***_, ${\varvec{M}}$∈ℝ ^*n*×*n*^ denotes the mask matrix whose parameters that GNNExplainer aims to learn, $\odot$ denotes element-wise multiplication, and $\sigma$ denotes the sigmoid that maps the mask matrix to [0, 1]^*n*×*n*^. The mask matrix $M$ is equal to the size of adjacency matrix ${A}_{c}$. During computation, $M$ is at first randomly initialized (referred to as initial mask matrix) and its real-valued parameters (coefficients) are adjusted to optimize the following loss:

9 $$loss=CrossEntropy\left(\Phi \left({G}_{c},\boldsymbol{ }M\right),Label\right)$$

where $CrossEntropy\left(\Phi \left({G}_{c},\boldsymbol{ }M\right)\right)$ is the cross entropy between the label and the prediction with edges masked out. In general, to explain the prediction ${\widehat{y}}_{i}$ for node ${v}_{i}$, if removing an edge between ${v}_{j}$ and ${v}_{k}$ strongly decreases the probability of prediction ${\widehat{y}}_{i}$, then the absence of this edge (${v}_{j}, {v}_{k}$) is a good counterfactual explanation for prediction ${\widehat{y}}_{i}$. It means the edge (${v}_{j}, {v}_{k}$) is of significant importance to the label of ${v}_{i}$. Conversely, if the removal of (${v}_{j}, {v}_{k}$) does not decrease the probability of prediction ${\widehat{y}}_{i}$, then this edge is not important to ${v}_{i}$ (Additional file 1: Fig. S14).

In this way, to explain the prediction for a given node, GNNExplainer assigns each edge an importance score and gives a ranking edge list. Likewise, GNNExplainer also learns a feature selector $F$ for nodes in explanation ***G***_***s***_ to generate ***X***_***s***_. Explanations (***G***_***s,***_*** X***_***s***_) are jointly optimized for maximizing a modified objective of mutual information in Eq. (6). Note that as current GNN-based models all use a layer-wise rule to propagate information and update embeddings for all nodes, therefore, for each prediction site, the edge masking only needs to be performed inside the scope covered by the propagation-involved area. In our work, DSB-GNN is a three-layer model and we thus performed the edge masking within the area of maximum 2-hop (on average 91 1-hop interactions and 9446 2-hop interactions per node), that is, the FaCIN was set to be a subgraph within 2-hop regions. In brief, for each node, GNNExplainer provides a set of explanation of edge list and node feature list that are ranked by importance.

### Definition of FaCIN and elements of its bottleneck pattern

Each node has its own FaCIN which is defined by its most influential edges and features according to GNNExplainer’s interpretation. FaCIN is a connected graph denoted as **G_Bottleneck = {N, E}**, where ***N*** represents the node set and ***E*** represents the edge set. Let ***n***_*p*_ denote the prediction site in FaCIN. ***E*** edge set is defined as no more than 10 edges that are most influential to ***n***_*p*_. An ***e***_(*i,j*)_ in ***E*** corresponds to an interaction on Hi-C contact map, and the pair of nodes ***n***_*i*_ and ***n***_*j*_ joined by ***e***_(*i,j*)_ correspond to the genome regions connected by the interaction. Each edge ***e***_(*i,j*)_ needs to satisfy:

$$\left\{\begin{array}{l}{{\varvec{n}}}_{i}\epsilon \{{{\varvec{n}}}_{p}, {{\varvec{N}}}_{1h}\left({{\varvec{n}}}_{p}\right), {{\varvec{N}}}_{2h}\left({{\varvec{n}}}_{p}\right)\}\\ {{\varvec{n}}}_{j}\epsilon \{{{\varvec{n}}}_{p}, {{\varvec{N}}}_{1h}\left({{\varvec{n}}}_{p}\right), {{\varvec{N}}}_{2h}\left({{\varvec{n}}}_{p}\right)\}\\ i \ne j\\\Delta {{\varvec{v}}}_{({\varvec{i}},{\varvec{j}})}\;\text{ranks in Top10, if}\;{\varvec{e}_{(i,j)}}\;\text{removed}\end{array}\right.$$

where ***N***_*1h*_(***n***_*p*_) denotes 1-hop neighbors of ***n***_*p*_, that is, the nodes whose shortest path to ***n***_*p*_ only consists of one edge. Likewise, ***N***_*2h*_(***n***_*p*_) denotes 2-hop neighbors, the nodes whose shortest path to ***n***_*p*_ consists of two edge. For example, if prediction site ***n***_*p*_ interacts with node ***b***, node ***b*** interacts with node ***c***, but there is no interaction between ***c*** and ***n***_*p*_. Then, ***b*** is a 1-hop neighbor of ***n***_*p*_ and ***c*** is a 2-hop neighbor. $\Delta {{\varvec{v}}}_{({\varvec{i}},{\varvec{j}})}$ is calculated by GNNExplainer and represents the difference of ***n***_*p*_ prediction scores before and after removing an edge*** e***_(*i,j*)_. The difference value is positively correlated to the contribution of edge ***e***_(*i,j*)_ to correctly predict ***n***_*p*_.

Please note that the shortest path mentioned above is defined on Hi-C contact map. For nodes ∈ ***N***_*2h*_(***n***_*p*_), their shortest paths to*** n***_*p*_ are not necessarily present in FaCIN unless with a Top 10 ranking in $\Delta {\varvec{v}}$. If a node whose total interactions on Hi-C contact graph are less than 10, its ***E*** edge set includes just these interactions.

In accordance with the bottleneck pattern of FaCIN (As described in the main body of the text), we renamed the 1-hop neighbors as neck neighbors, and the interactions between prediction site and neck neighbors as neck interactions. In FaCIN, the definition of neck neighbor is actually: (i) a node directly interacts with prediction site on Hi-C contact graph, and satisfies that (ii) removing the neck interaction will affect DSB-GNN’s decision-making on prediction site and leads to a variation of prediction score that ranks higher than that can be led to by removing other nodes. The length of neck interaction is the genomic distance between a pair of loci and the intensity of neck interaction represents the number of contacts between the loci. 2-hop neighbors are renamed as other neighbors, and the interactions between prediction site and 2-hop neighbors as other interactions accordingly.

### Calculation of betweenness centrality

In graph theory, the betweenness centrality for each node is the number of all shortest paths between any pairs of nodes that pass through this node. It actually measures how well a node is connected across the whole graph. In FaCIN, as we focus on the label of prediction site (being DSB or non-DSB), we only consider how often a 1-hop or 2-hop neighbor appears on the shortest path between other nodes and prediction site, rather than all pairs of nodes.

### Randomized graph generation

We generated randomized graphs using Random Walk on raw Hi-C contact maps and restricted them to have the same overall characteristics as the FaCIN: a connected subgraph for a genome bin that covers at maximum a two-hop region with no more than 10 edges.

### Subgraph search for motif

The search for topological subgraph aims to find whether a set of graphs contain enriched motifs. For all the FaCINs obtained from an individual chromosome, the search is performed as follows:

1. Create an empty list in which to store the subgraph and its occurrence number.
2. Examine a FaCIN and use an “is_isomorphic” function from NetworkX [60] (a Python package) to determine whether current FaCIN is identical to any existing subgraph in list. If it is, the number of corresponding subgraph plus 1. If it is not, store the current FaCIN as a new subgraph in the list. Continue this step until examining all FaCINs.
3. At last, count occurrence numbers for all present subgraphs and only those subgraphs with significantly higher occurrence number can be taken as motifs.

Above results are organized in a chromosome-wise manner (Additional file 3) and exhibited in a simplified form unlike in a complete form with neighboring information in Fig. 2a (bottom). The cascade motif and bifurcate motif are the top-2 out of all candidates which account for over 80% FaCINs on whole genome (Additional file 1: Fig. S6a). To avoid misunderstanding, we take cascade motif as an example and provide its more detailed illustration in Additional file 1: Fig. S6b.

### Neck interaction in TAD

We obtained 2832 TADs of the Hi-C data for NHEK provided by Rao et al. [31]. The TADs are defined as intervals bounded by a start coordinate and an end coordinate. Each neck interaction connects two genome bins and the genomic distance between these two bins is referred to as the interaction’s length. With appropriate orientation, if the end coordinate of the first bin and the start coordinate of the second bin both locate in a TAD’s interval, we referred to this neck interaction as in TAD. We then extracted all neck interactions and identified all neck interactions in TAD using command of “-e -1” with BEDOPS [61]. Furthermore, we generated 10 sets of artificial interactions that randomly locate in genome but restricted them to share the same length distribution with the neck interactions. We then calculated the number of the artificial interactions in TAD and used the results to provide a global background for comparison.

### Identification of enhancer-promoter loop

We downloaded the enhancers from a highly recognized database EnhancerAtlas 2.04 [62]. In this database, Gao et al. defined enhancers based on 12 high-throughput experiment methods. They developed an unsupervised learning approach to weigh each track of experiment methods and combined them to determine the consensus enhancers. The promoters are defined as the intervals (− 2000 bp to + 2000 bp) around a transcriptional start site (TSS). If a 5-kb genome bin overlaps with a promoter (an enhancer), then we refer to it as a promoter node (enhancer node). Then, an interaction that connects one enhancer and one promoter was used as E-P loop.

### Neck interaction being loop

We first specify the data source of our used loops. From 3D Genome Browser (http://3dgenome.org), the loops (19,632 in total) provided by a computational method Peakachu [63] were directly downloaded. Peakachu has demonstrated the validity and reliability of their predicted chromatin loops genome-wide. Next is about the details of calculation. The loops are specified by two anchors and each anchor is a small region with a start coordinate and an end coordinate. Each neck interaction connects two genome bins. We defined the middle coordinate of the first bin as the interaction’s start and the middle coordinate of the second bin as the interaction’s end. If a neck interaction’s start and end respectively locate in two anchors of a loop, it is called a neck interaction being loop or constituting loop. We calculated the start and end for all neck interactions and identified the number of them being loops. Again, the same calculation was performed on those artificial interactions to provide a global background.

## Supplementary Information

Additional file 1: Fig. S1. AUROC of DSB-GNN on each chromosome for NHEK cell line and performance comparison. Fig. S2. The distribution of the importance score of all node features and the top 10 k-mer features. Fig. S3. The comparison between the numbers of direct chromatin interactions and neck interactions for a prediction site on average. Fig. S4. Details for FaCIN’s bottleneck pattern. Fig. S5. An illustration for betweenness centrality and the comparison between neck neighbours and other neighbours in terms of betweenness centrality. Fig. S6. Results of subgraph search on FaCIN and random graph. Fig. S7. A schematic comparison between bottleneck and cycle patterns. Fig. S8. The 1D genomic length spanned by FaCIN’s interactions for 1-hop and 2-hop interactions. Fig. S9. The description of hypergeometric test for neck interactions and loop interactions.Fig. S10. Neck interactions in TAD boundaries. Fig. S11. The average length and intensity of neck interactions at DSB and non-DSB sites. Fig. S12. Percentage for neck neighbours of DSB prediction sites. Fig. S13. Statistics of the raw Hi-C contact counts. Fig. S14. A schematic for GNNExplainer masking approach to identify important edges.

Additional file 2: Table S1. Robustness evaluation for DSB-GNN against different Hi-C processing parameters. Table S2. Results of ablation experiments. Table S3. Performance comparison between DSB-GNN, LightGBM and Random Forest with different subsets of features. Table S4. The distribution of 1D genomic distance from prediction site to 1-hop and 2-hop neighbours. Table S5. 1242 loops with at least one anchor developing DSBs. Table S6. Comparison of different thresholds for contact counts. Table S7. Comparison of different numbers of GAT or GCN layers.

Additional file 3: File S1. The top-5 motifs of FaCINs for each chromosome.

Additional file 4 .

## Data Availability

In this work, the used Hi-C data and DSB data of NHEK cell line are respectively obtained from Rao et al. [31] and the NCBI Gene Expression Omnibus with accession number GSE78172 [7]. The corresponding ChIP-seq data of CTCF and DNase-seq data are retrieved from ENCODE project [51]. Source codes for running DSB-GNN are available on GitHub (https://github.com/Ranceeeee/DSB_GNN) under the MIT license and these codes are also deposited at Zenodo: https://zenodo.org/record/7750113 [64].
