# Supplementary material for: A graph neural network-based interpretable framework reveals a novel DNA fragility–associated chromatin structural unit
Source: Genome Biol. 2023 Apr 24;24:90. doi: 10.1186/s13059-023-02916-x (PMC10124043; doi:10.1186/s13059-023-02916-x)

Fig. S1 a Area under the receiver operating characteristic curve (AUROC) > 0.92 for each chromosome. **b** Comparison between DSB-GNN with method proposed by Mourad et al.

a.

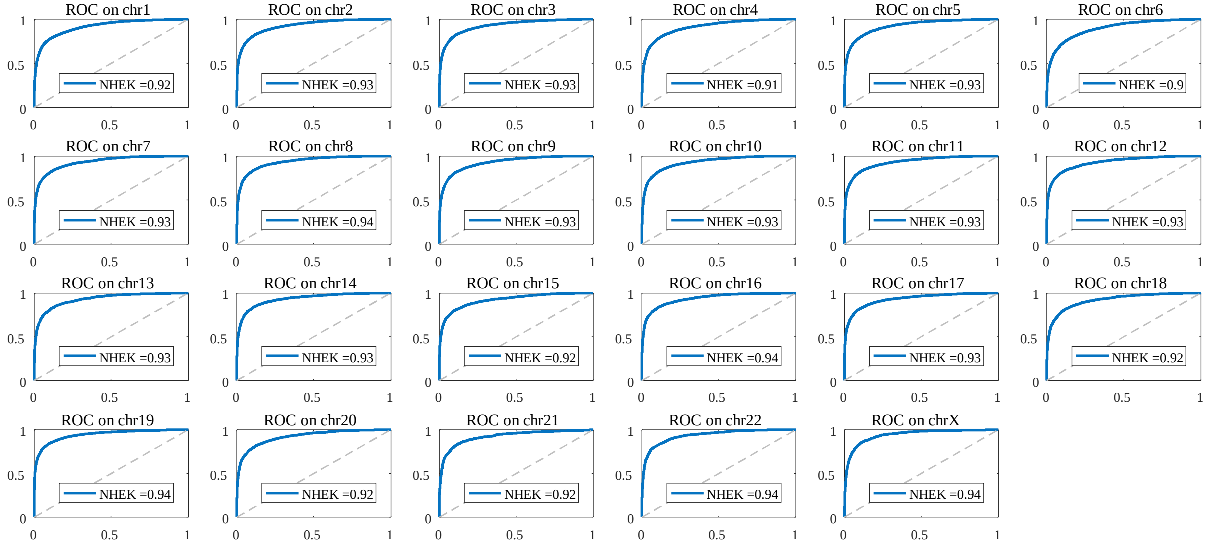

b.

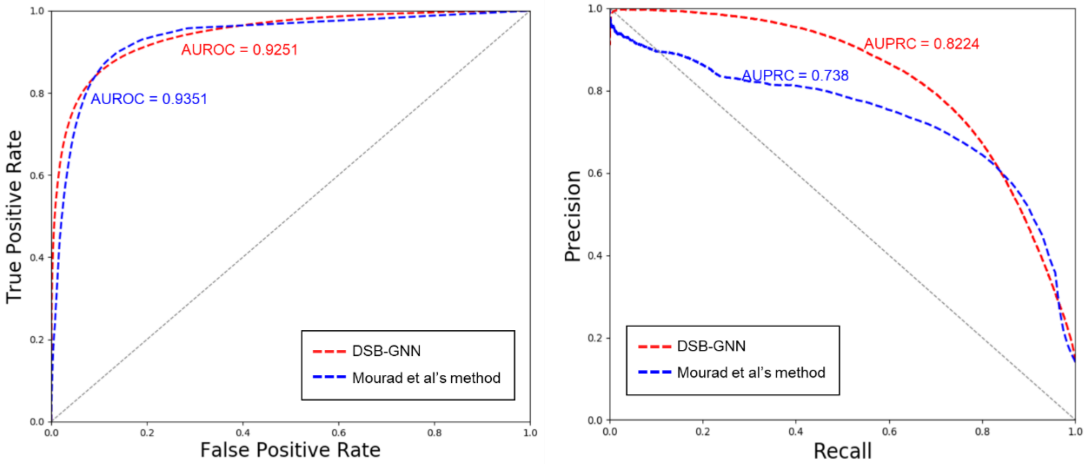

Fig. S2 a CTCF and DNase I signals are the most important two features among all 1346 features. **b** 6 out of the top 10 K-mer features have a 4-bp overlap with DSB preference sequence.

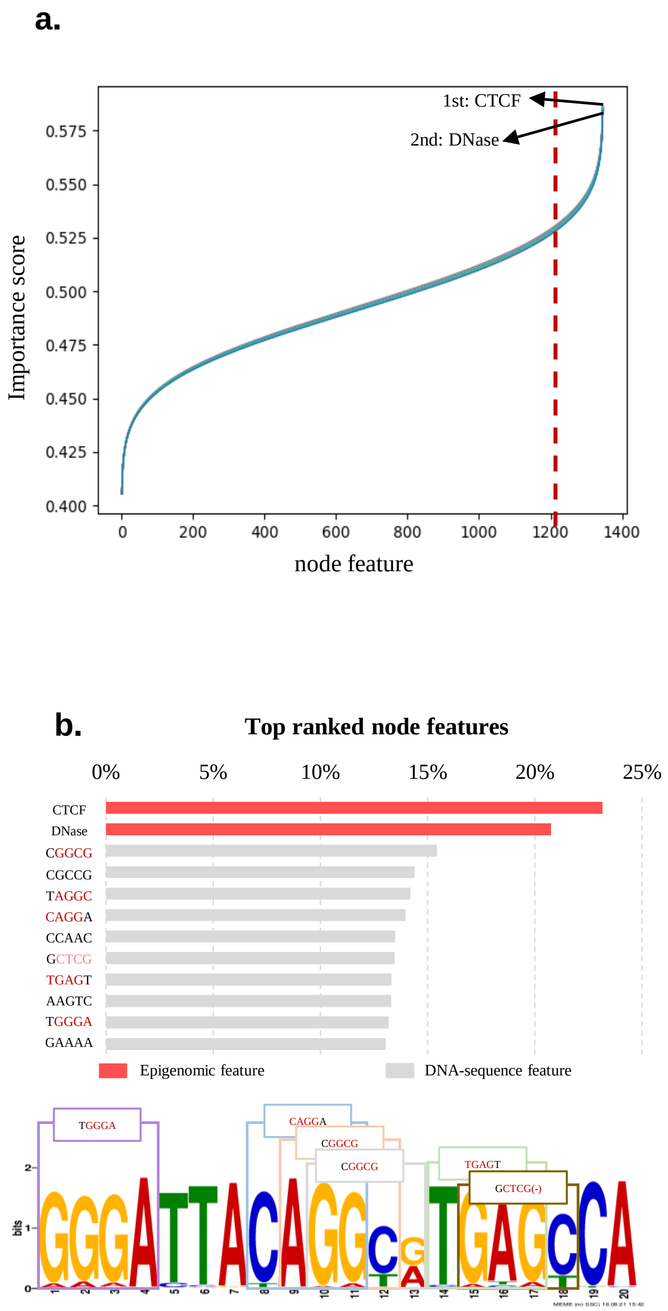

Fig. S3 One prediction site has an average of 91 direct chromatin interactions (blue) while among them only 1.6 are neck interactions (orange). The gray bar indicates the upper limit of interactions in FaCIN.

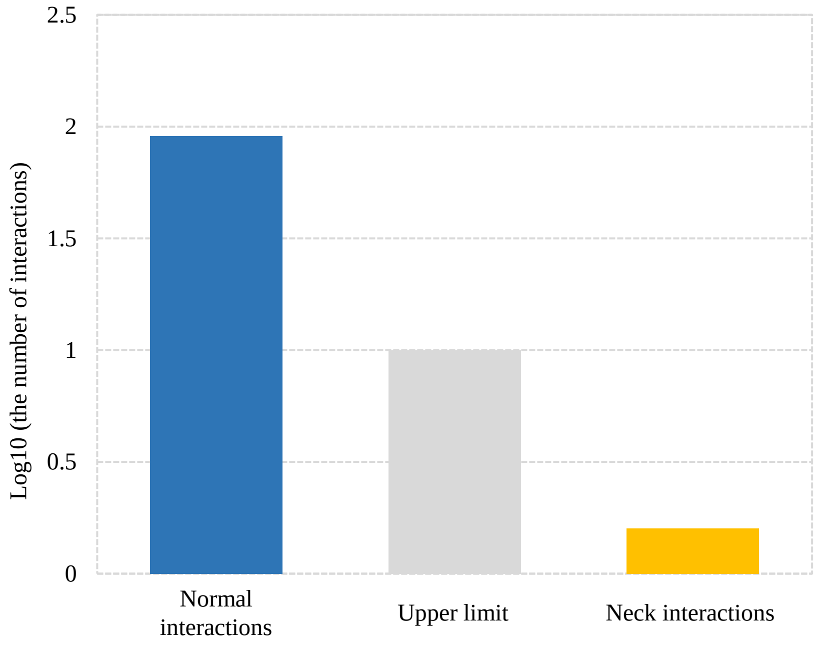

Fig. S4 More details for FaCIN's bottleneck pattern. **a** The left gives a local view under the 3D space. Tangled lines represent the intricate folding of chromatin, and different line colors are used for better distinction. Chromatin is binned and grey short thick lines are used to mark consecutive 5-kb genome bins. If we read along the line coloured blue, we will find one by one the genome bins noted as *a*, *b*, *c*, *d*, *e* and so on. Likewise, the olive one consists of the ordered genome bins *f*, *g*, *h*, *i*, *j* and *k*; the off-white one consists of the bins *l*, *m*, *n*, *o*, *p*, *q* and *r*. Highlighted regions represent the physical contacts between bin pairs. **b** The right is a simplified schematic of the left one, organized as a graph where nodes represent for genomic bins and edges for the interactions. The subgraph with a light grey background is the FaCIN of node a. Take *a* as the prediction site, the only two interactions directly link *a* to *l* and *f* are neck interactions, therefore *l* and *f* are named *a*'s neck neighbours; further, the neck neighbours connect with nodes of fastly expanding number. These two contrastive parts jointly form a topological shape suggestive of a bottle. Viewed from the *a* prediction site, FaCIN's interactions form a shape going from narrow to wide that visually resembles a bottleneck. Out of an intuitive purpose, we describe FaCIN's pattern as bottleneck-like.

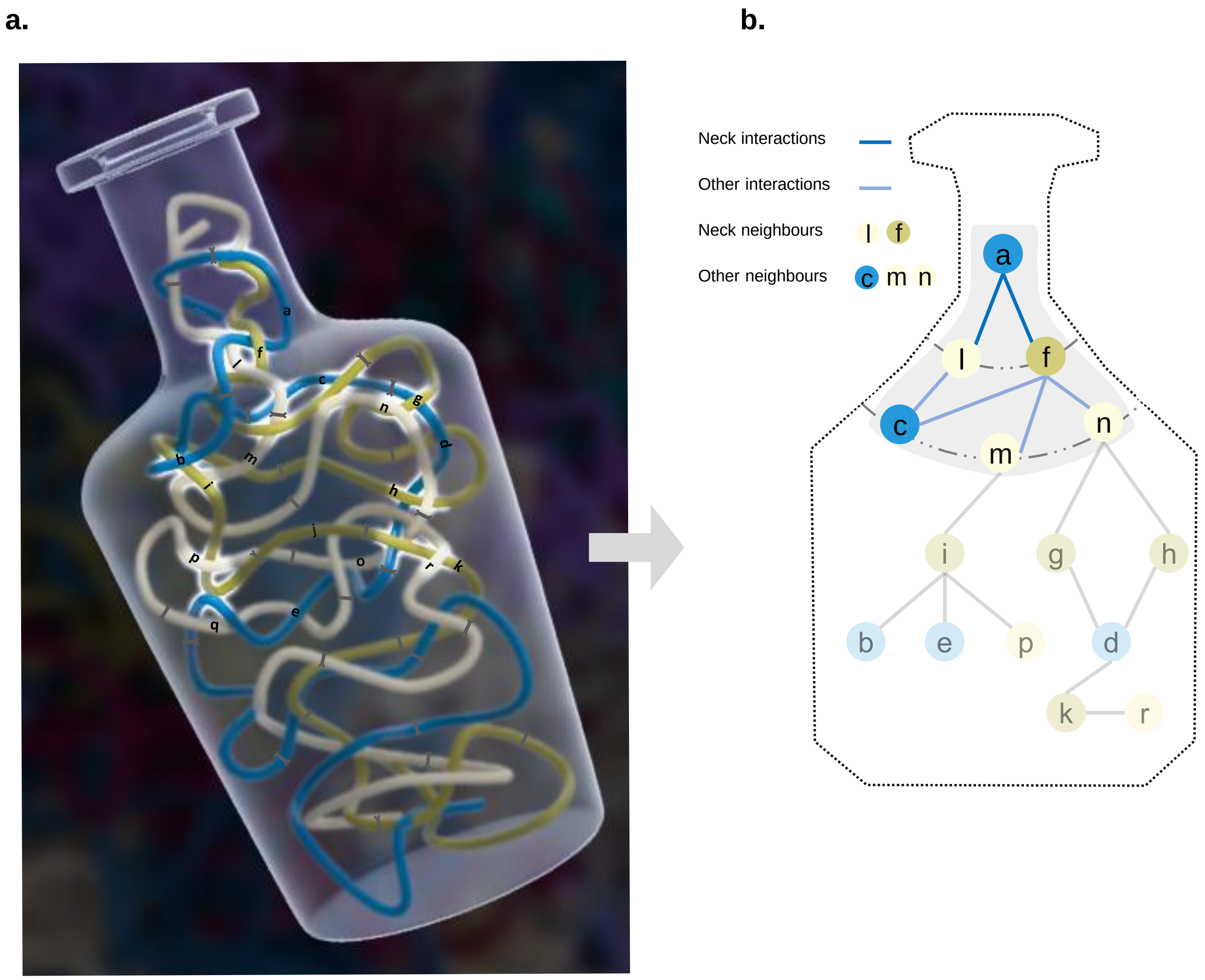

Fig. S5 a An illustration for calculation of betweenness centrality. On this tiny graph, the betweenness centrality of the yellow node for the red-green node pair is 2/3 = 0.667, as the number of shortest paths between red-green node pair is 3 and among them the yellow node appears twice. **b** Betweenness centrality of neck (1-hop) neighbours and other (2-hop) neighbour for node pairs of *(prediction site, any other node).* The p-value was calculated using t-test.

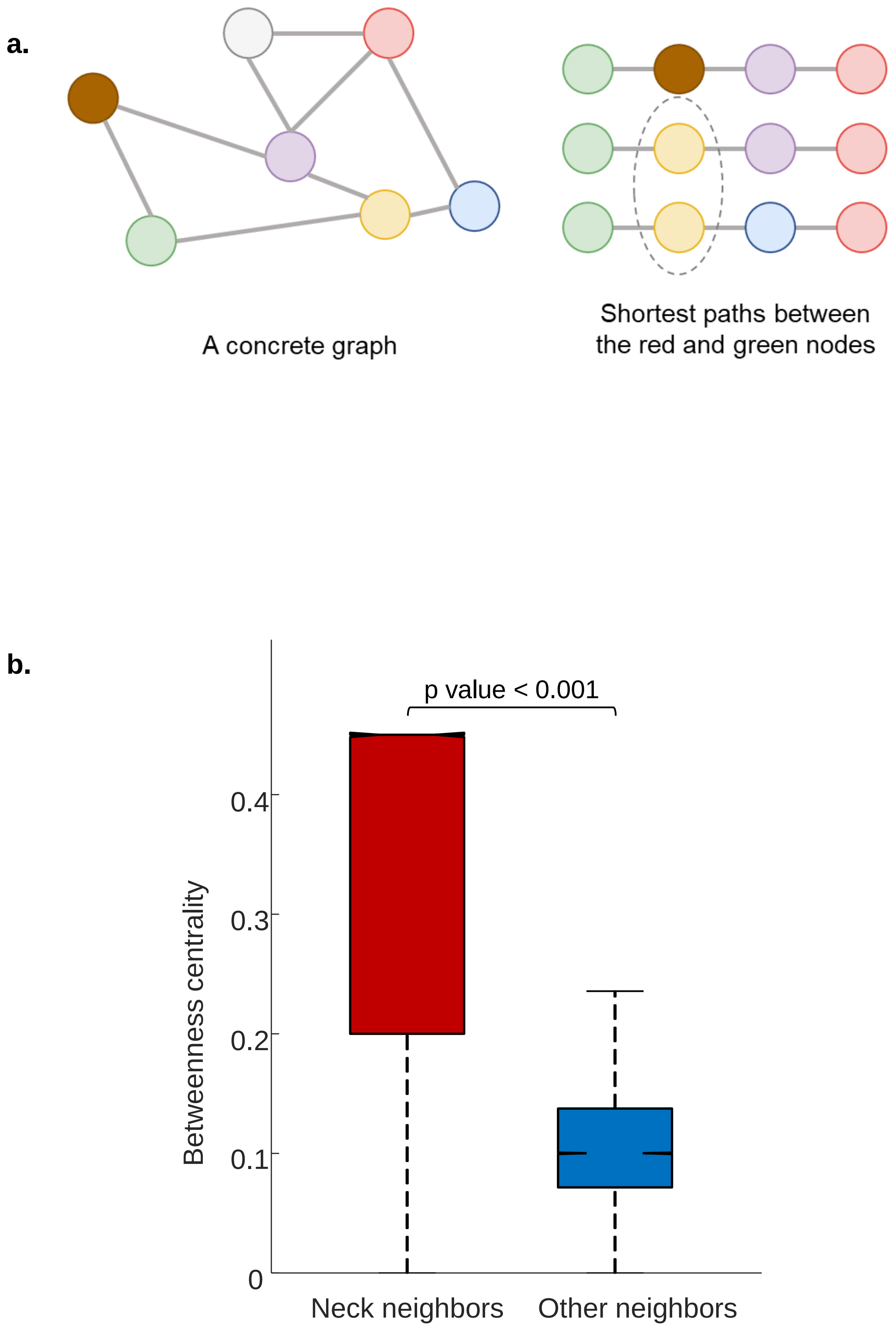

Fig. S6 Results of subgraph search on FaCIN and random graph. **a.** Top 5 subgraphs for FaCINs (up) and randomized graph (bottom). The cascade motif and bifurcate motif are the top 2 out of all candidates which account for over 80% FaCINs on whole genome. While on randomized graphs, subgraphs show no enrichment of any motif and account for patterns that appear merely due to the general Hi-C interactions. **b.** The illustration of cascade motif in a complete form. Cascade motif involves six nodes and the prediction site can appear at any position. Considering the symmetry will reduce the possible positions of predicted site from six to three. Once the prediction site is determined, identifying its 1-hop and 2-hop neighbours will be fairly straightforward.

a.

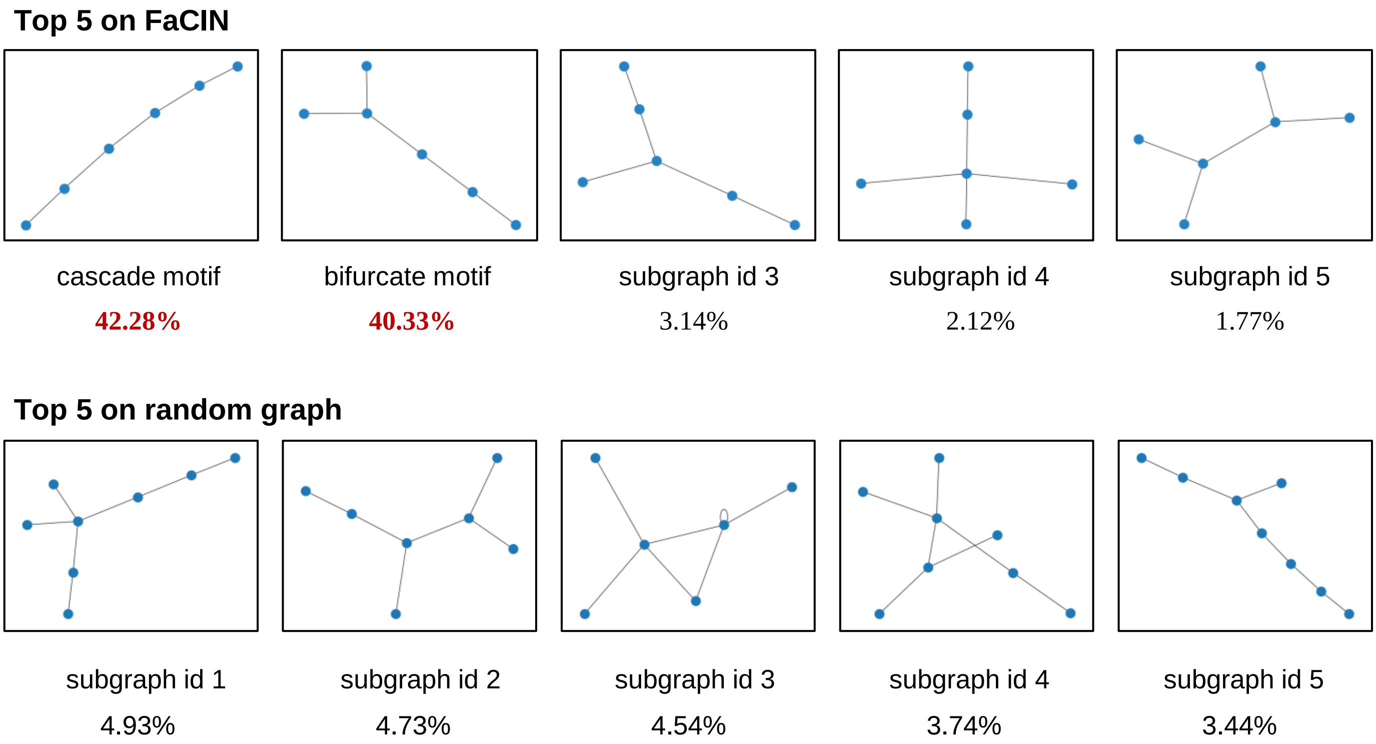

b.

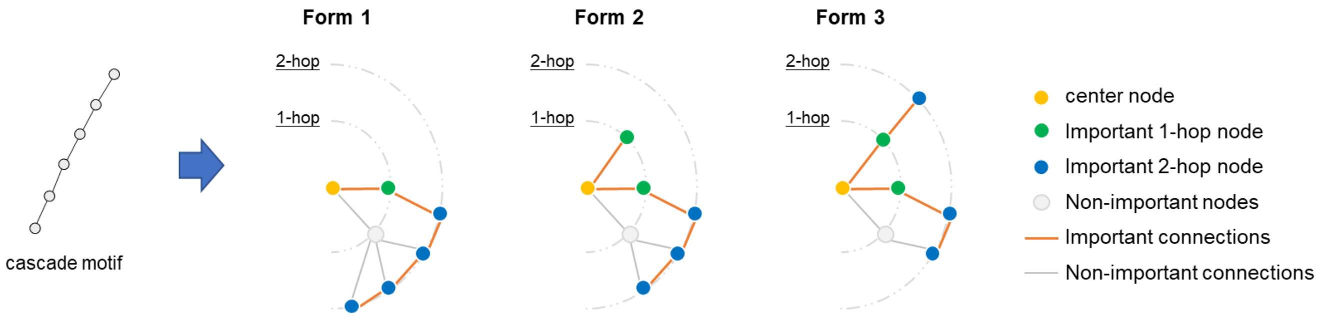

Fig. S7 Schematic comparison between bottleneck (left) and cycle (right) patterns. Overlook the node types, then these two graphs are isomorphic as they all contain the same number of nodes connected in the same way. However, a FaCIN cannot be determined unless its prediction site is determined first. Therefore, the nodes should not be treated without distinction. Bottleneck pattern is a manner where the prediction site directly communicates with one neck neighbor and the neck neighbor gathers biological information from far more genome regions at distance. Cycle pattern describes an entirely different manner where the prediction site is evenly affected by multiple neighbors around.

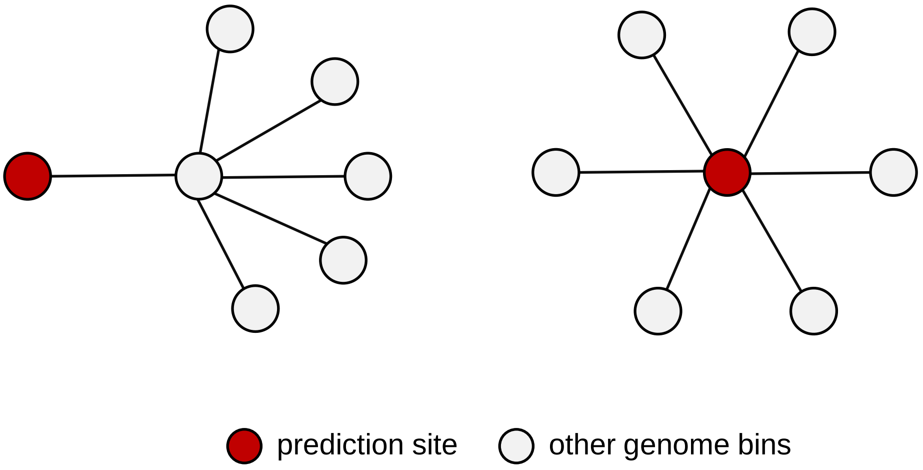

Fig. S8 The 1D genomic length spanned by FaCIN's interactions for **a.** 1-hop interactions and **b.** 2-hop interactions.

a.

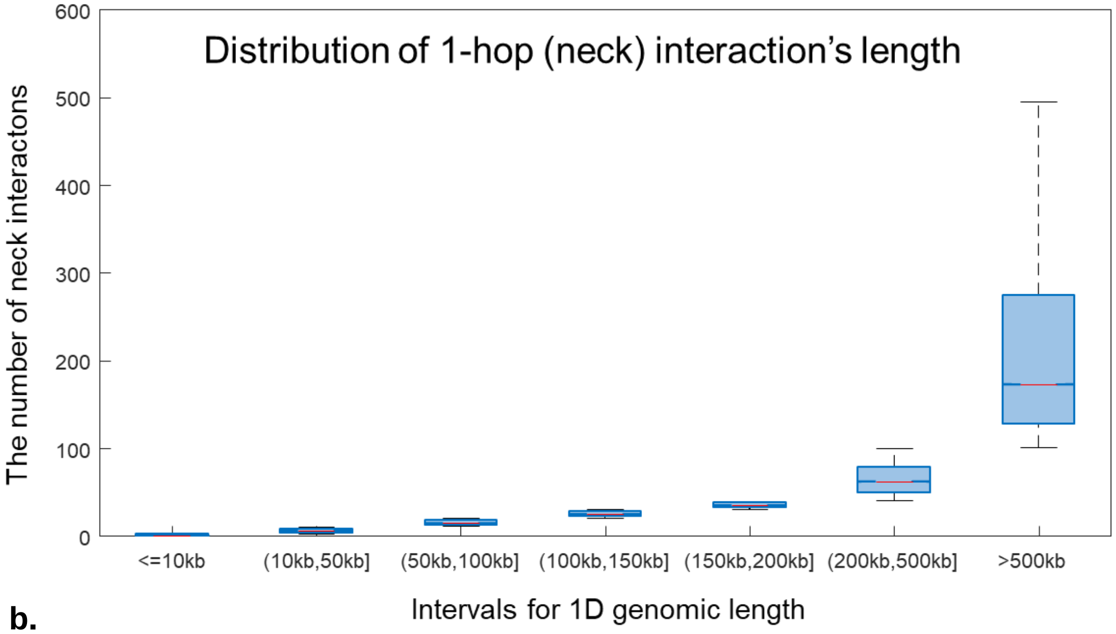

b.

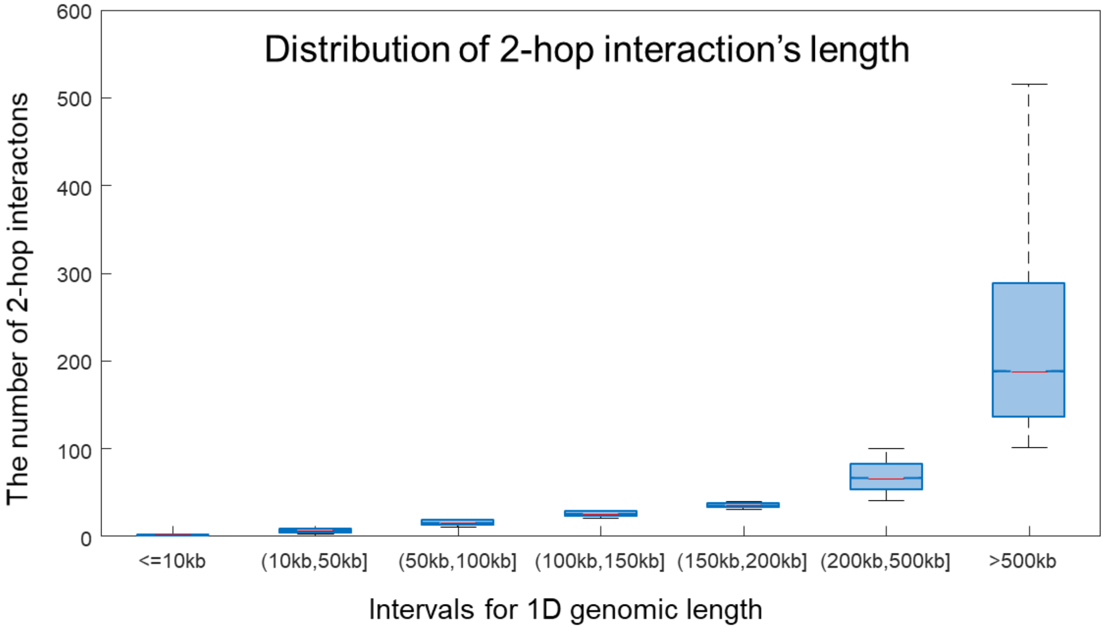

Fig. S9 Description of hypergeometric test for neck interactions and loop interactions. the upper figure was the illustration of loop interactions and non-loop interactions, and the lower figure was used to show neck interactions at the same genomic regions as the upper figure. Four numbers were used to perform hypergeometric test: N indicates the number of the whole-genome interactions; M indicates the number of loop interactions; n indicates the number of all neck interactions, and k indicates the number of neck interactions which are also loop interactions. N, M, n, k is 25917290, 19632, 865635, 1150, respectively. We calculated the probability that at least k neck interactions are also loop interactions, not exactly k neck interactions. We performed R cmd “phyper (k-1, M, N, n, lower.tail = FALSE) and got p-value = 4.30e-71.

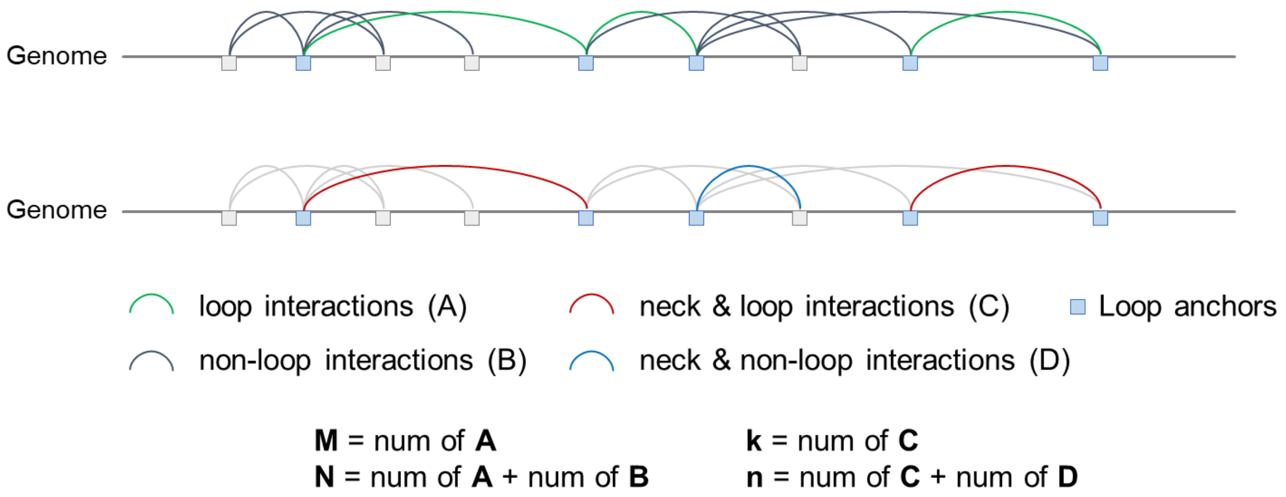

Fig. S10 Neck interactions are significantly enriched in TAD boundaries. For an interaction that joins the two boundary loci of a particular TAD, we refer to it as an interaction in TAD boundary. Neck interactions in TAD boundary is 85, nearly triple that average number of random interactions (randomly selected from the whole genome with the same number as the total neck interactions, 100 repeats). Significance is calculated using hypergeometric test.

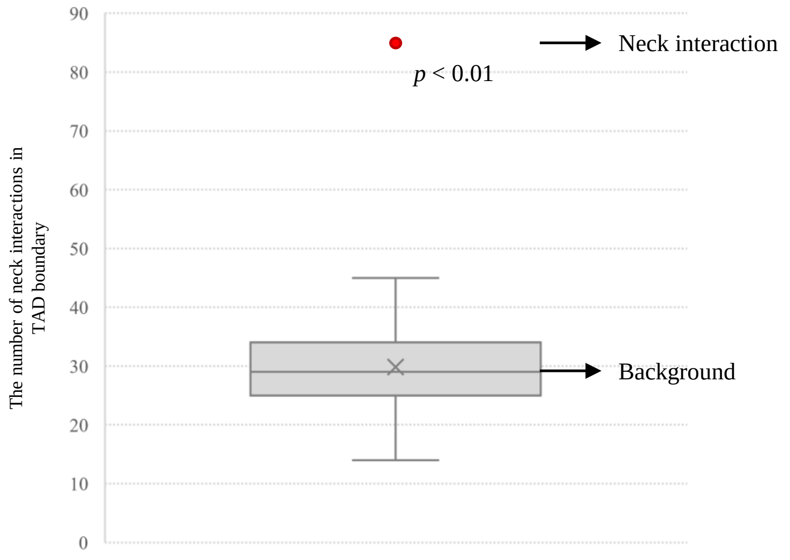

Fig. S11 **a** The average length of neck interactions at DSB and non-DSB sites. **b** The intensity of neck interactions at DSB and non-DSB sites.

a.

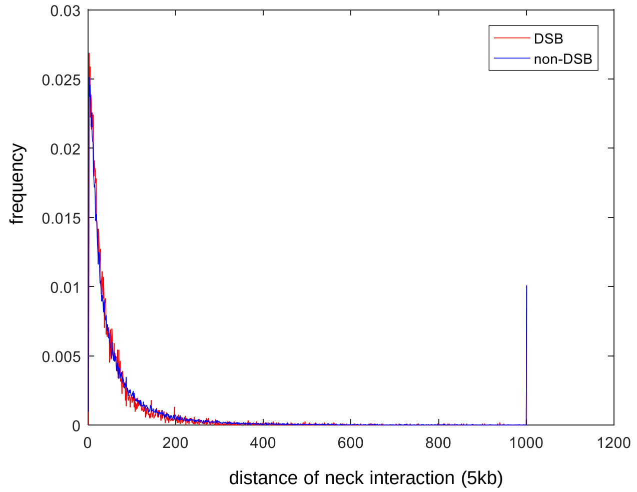

b.

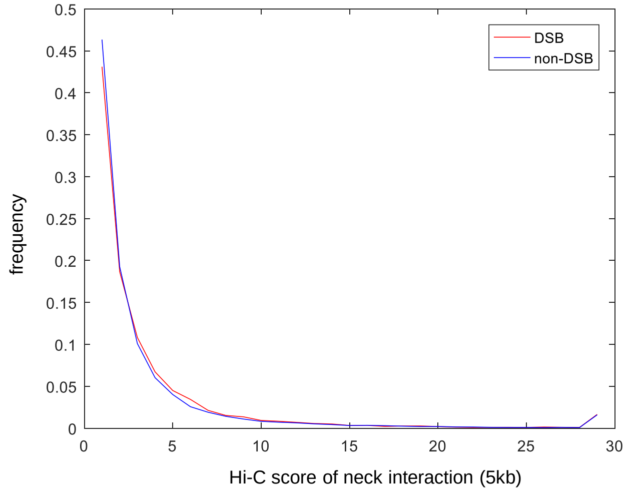

Fig. S12 Percentage for neck neighbours of DSB prediction sites.

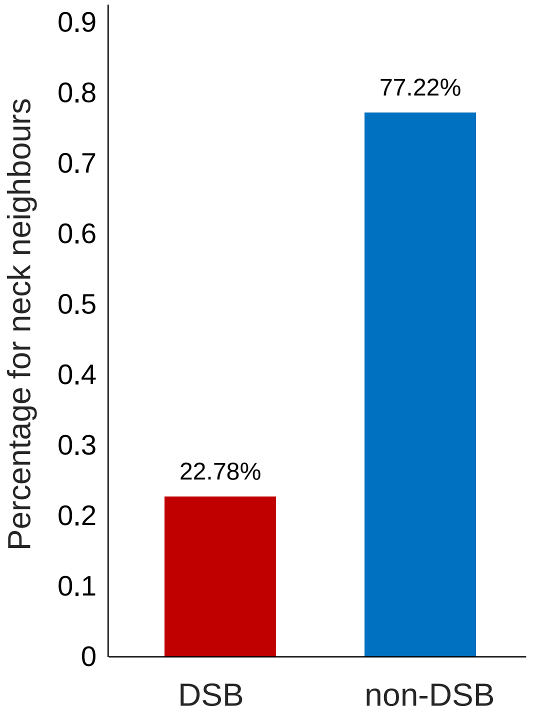

Fig. S13 **a** Distribution of raw contact counts. **b** Number of interactions under different thresholds.

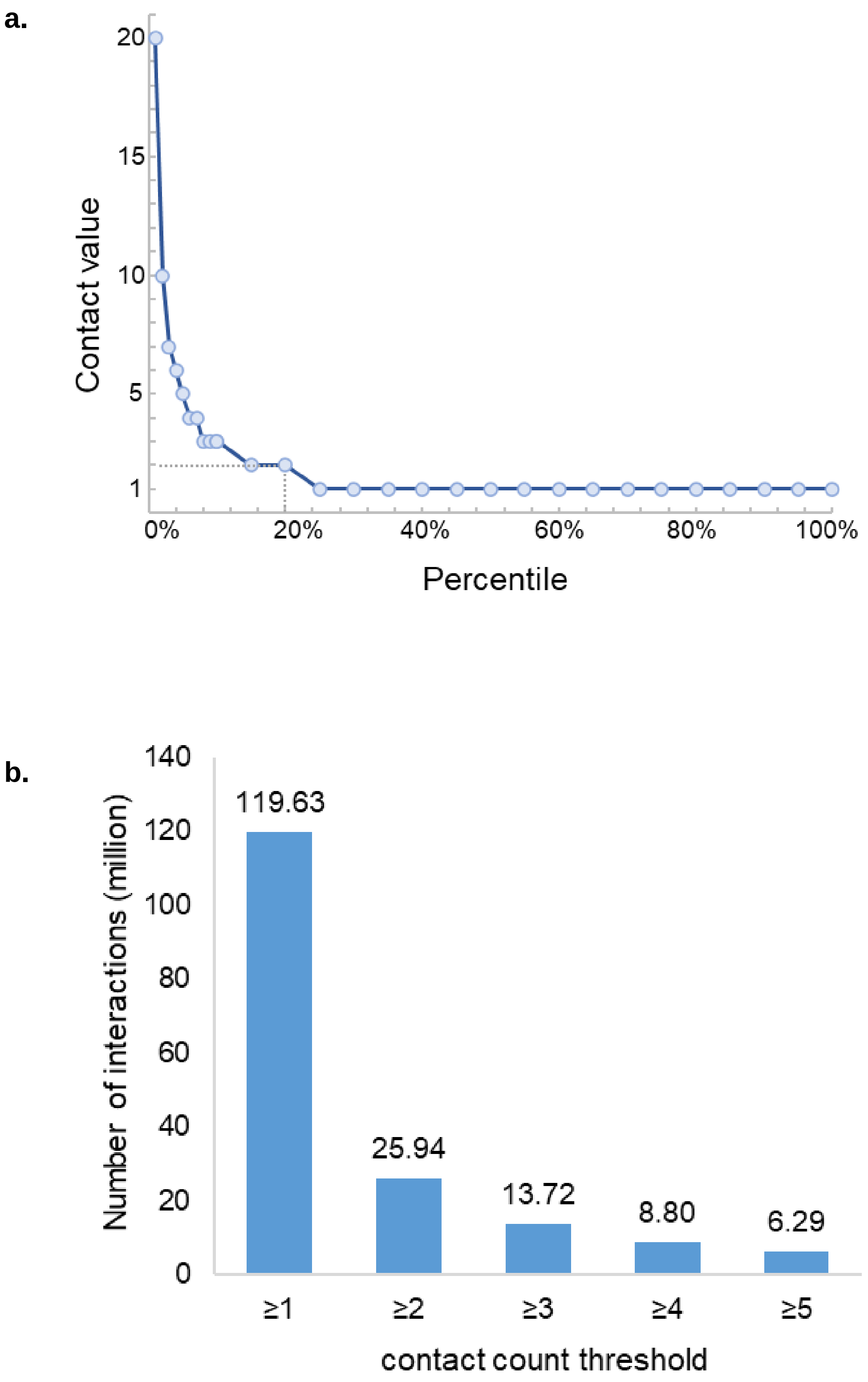

Fig. S14 Schematic for GNNExplainer masking approach to identify important edges.

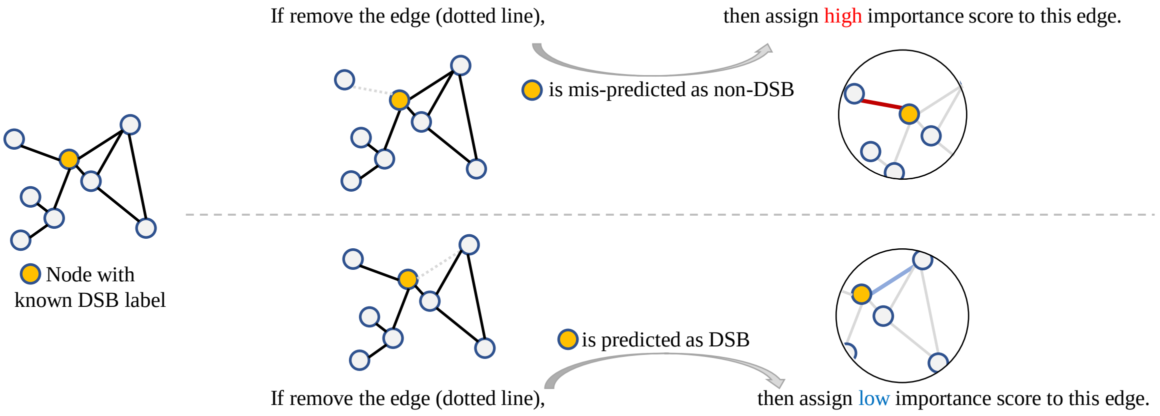

Supplement: Supplementary file 1 — Additional file 1: Fig. S1. AUROC of DSB-GNN on each chromosome for NHEK cell line and performance comparison. Fig. S2. The distribution of the importance score of all node features and the top 10 k-mer features. Fig. S3. The comparison between the numbers of direct chromatin interactions and neck interactions for a prediction site on average. Fig. S4. Details for FaCIN’s bottleneck pattern. Fig. S5. An illustration for betweenness centrality and the comparison between neck neighbours and other neighbours in terms of betweenness centrality. Fig. S6. Results of subgraph search on FaCIN and random graph. Fig. S7. A schematic comparison between bottleneck and cycle patterns. Fig. S8. The 1D genomic length spanned by FaCIN’s interactions for 1-hop and 2-hop interactions. Fig. S9. The description of hypergeometric test for neck interactions and loop interactions.Fig. S10. Neck interactions in TAD boundaries. Fig. S11. The average length and intensity of neck interactions at DSB and non-DSB sites. Fig. S12. Percentage for neck neighbours of DSB prediction sites. Fig. S13. Statistics of the raw Hi-C contact counts. Fig. S14. A schematic for GNNExplainer masking approach to identify important edges. [file 13059_2023_2916_MOESM1_ESM.pdf]
