# Supplementary material for: A graph neural network-based interpretable framework reveals a novel DNA fragility–associated chromatin structural unit
Source: Genome Biol. 2023 Apr 24;24:90. doi: 10.1186/s13059-023-02916-x (PMC10124043; doi:10.1186/s13059-023-02916-x)
Supplement: Supplementary file 3 — Additional file 3: File S1. The top-5 motifs of FaCINs for each chromosome. [file 13059_2023_2916_MOESM3_ESM.docx]

Additional file 3 of

**Interpretation on graph neural network reveals a novel DNA fragility–associated chromatin structural unit**

**Content:**

The top-5 motifs of FaCINs for each chromosome. From left to right, motifs are organized in descending order of occurring frequency.
