# Supplementary material for: A graph neural network-based interpretable framework reveals a novel DNA fragility–associated chromatin structural unit
Source: Genome Biol. 2023 Apr 24;24:90. doi: 10.1186/s13059-023-02916-x (PMC10124043; doi:10.1186/s13059-023-02916-x)
Supplement: Supplementary file 4 — Additional file 4 . [file 13059_2023_2916_MOESM4_ESM.docx]

Review History

**First round of review**

**Reviewer 1**

**Are you able to assess all statistics in the manuscript, including the appropriateness of statistical tests used?** There are no statistics in the manuscript.

**Comments to author:**

In this manuscript, the authors examined the potential associations between the 3D genome structural features and DNA double-strand breaks (DSBs) by proposing a GNN-based and interpretable framework. The idea of implementing GNNExplainer into this framework is innovative and promising. The FaCIN unit and its bottleneck pattern are interesting and may be meaningful for further study. Please find the following detailed comments:

1. As the authors described, they proposed a framework that integrates deep graph neural network (GNN). However, a general limitation of GNN is its layers cannot be too deep, which is well-known as the over-smoothing issue. The authors should provide more details in model construction, and it is also important to discuss how they deal with the over-smoothing issue in their framework.

2. As the authors described, FaCINs is identified as a new unit for understanding DSBs. DNA lesions are important issue and DSB is a key type of DNA lesions. It is necessary to compare FaCINs with known DSB elements and further provide more insights, for example, how FaCINs associated with DSB repair.

3. The biological meanings of "the 1242 loops with DSBs on one anchor and another linked by neck interactions" (Line 193). Further analysis is needed.

4. I suggest the authors analyze the size of FaCIN, that is, calculate the distance between FaCIN node and its one-hop and two-hop neighborhoods, and examine the DNA elements in that genomic region. This may be important for gene regulation and even DSB repair.

5. What hypothesis tests the authors used are missed, Line 170, 194, Fig. 4d.

6. Fig. 4e, the y-label, "Dnase signal" should be "DNaseI signal" or "DHS signal".

7. Fig 1.a, the number of 3-mer seq feature is 64 but not 256.

**Reviewer 2**

**Are you able to assess all statistics in the manuscript, including the appropriateness of statistical tests used?**Yes, and I have assessed the statistics in my report.

**Comments to author:**

In this work, Sun et al. described a GNN-based method for predicting double-strand break (DSB) sites based on Hi-C contacts, sequence features, and chromatin (accessibility and CTCF binding) signals. The authors also used a method called GNNExplainer to interpret the models and identify features important for the predictions, based on which the concept of DNA fragility-associated chromatin interaction network (FaCIN) was proposed and some analyses related to it were performed.

Major comments:

1. No strong justifications are provided for the use of complex GNN in predicting DSB sites:

a. If the purpose is to how that the prediction performance is improved, comparison with other existing methods and ablation studies (with features only, with network structure only, with subsets of features only, without the self-attention mechanism, etc.) are needed to show the amount of improvement achieved.

b. If the purpose is to provide biological insights, the final conclusions of the study, such as enrichment of DSB sites at loop regions and accessible chromatin, seem not particularly novel.

c. Even with the interpretation method and the FaCIN concept, the manuscript offers limited insight into the general features of DSB sites. For instance, the authors have not explained how the chains and bifurcation motifs are generally related to DSB.

2. Regarding "bottleneck" and "neck interactions":

a. The key concept of "bottleneck" is not formally defined. The fancy illustrations in Figure 2a and Figure S2 do not help understand exactly what a bottleneck is, mathematically or in terms of genome architecture.

b. The term "neck interaction" is quite confusing. According to the description in the manuscript, "for a given genome bin of interest, we refer to this genome bin as prediction site. FaCIN of this prediction site consists of interactions and each of them belongs to neck interactions or other interactions, according to whether it directly connects the prediction site." -- So, no matter which genomic bin is taken as the prediction site, its direct interactions in the FaCIN are always called neck interactions?

3. The authors claim that they have used "deep graph neural network" in their work:

a. However, it seems that in the whole manuscript the number of GAT layers used is not stated.

b. It has been reported that having more GNN layers does not necessarily lead to better performance, sometimes even worse beyond 2-3 layers. It is therefore not clear why the authors advocate the "deep" nature of their network.

c. The authors have not compared the prediction performance with different numbers of GNN layers to justify the need for a "deep" network.

4. It is not certain from the manuscript how much the results are affected by Hi-C read depth and data resolution (i.e., bin size):

a. The robustness of the method with respect to these parameters have not been evaluated.

b. The NHEK Hi-C data set used does not have a high read depth, and therefore it is doubtful whether using 5kb bin size would lead to too much noise and sparsity.

5. Regarding the subset of Hi-C contacts used:

a. The authors have not justified the omission of inter-chromosomal interactions.

b. The authors have not carefully distinguished between proximal interactions more due to one-dimensional genomic proximity and distal interactions more due to three-dimensional genome architecture.

c. The authors have not explained what quantity they have used to indicate interaction strength in the edge weights.

6. Regarding the GNNExplainer method:

a. Exactly how masking is performed seems not clearly described. For instance, does it involve re-training the embedding and the classifier?

b. Computationally, how feasible is it to mask each edge one by one to evaluate its importance?

7. Different parts of the methodology, and correspondingly the results, may depend strongly on some threshold values:

a. Threshold for two genomic bins to be considered interacting according to a Hi-C data set. For instance, the "neck interaction" shown in Figure 5b does not look like a strong interaction.

b. Threshold for the amount of change in a prediction such that the omission of a Hi-C interaction is considered importantly affecting the prediction

8. "These motifs provided statistical evidence of the bottleneck pattern in FaCIN":

a. In fact, the authors have not provided any statistical evidence that these motifs are enriched given the Hi-C contact map. They have only said that the "forward chain" and "binary parallel" motifs are most frequent, but they may also be most frequent in the Hi-C interactions in general.

b. Even if the motifs are significantly enriched in the FaCINs, how this would provide statistical evidence of the bottleneck pattern is unclear.

9. Regarding the chromatin loops:

a. It is necessary to state their definitions.

b. The number (19,632) seems very small if "chromatin loops" simply mean reliable chromatin interactions.

c. The mentioning that "Loops are basically groups of interactions with special functions, such as enhancer-promoter (E-P) linkage" is confusing. Is functional role a requirement for a chromatin interaction to be called a chromatin loop? Shouldn't "chromatin loop" be a more general concept?

10. In the whole paper, when p-values are reported, the corresponding statistical tests are not specified.

Other comments:

1. Contacts in a Hi-C map may be due to 3D chromatin interactions or genomic rearrangements. The authors have not made any efforts to distinguish between them.

2. Transcription is described by the authors as "a threat to genome stability", but in fact transcription-coupled repair is also a major mechanism for maintaining genome stability.

3. "E edges represent Hi-C contact maps between nodes" -> "Edges in E represent Hi-C contacts between nodes"

4. "Its bottleneck pattern indicates that the aggregated information ultimately flows to one destination" -- Although the Hi-C interaction network is undirected, the GAT layers are ordered and therefore there is a direction of information flow. Does the "bottleneck pattern" really support "flows to one destination"?

5. "Motifs, such as transcription factor-binding site and regulatory network motifs" -- These are very different types of motifs that are seldom considered together.

6. "The most frequent motifs exhibited a 'forward chain' and 'binary-parallel' mode":

a. Why is it considered "forward"?

b. Why is it called "binary-parallel" here and "bifurcate" in Figure 2c?

7. "recent Hi-C experiments have generated more than 30 publicly available datasets" -- This sentence suggests that the authors do not really have a good idea of the amount of publicly available Hi-C data.

**Reviewer 3**

**Are you able to assess all statistics in the manuscript, including the appropriateness of statistical tests used?**Yes, and I have assessed the statistics in my report.

**Comments to author:**

The authors proposed a deep graph neural network (GNN) model to predict DSB from DNA sequence and 3D chromatin structure. They then applied GNNExplainer, a masking-based method to study feature importance, and defined "DNA fragility-associated chromatin interaction network" (FaCIN), a connected graph of top important features and edges predicted by GNNExplainer. They found that that direct interactions in FaCIN (which they called neck interactions) showed interesting associated with TAD and loops, and argued that they can serve as chromatin structural determinants of DSB formation. While the application of GNN and GNNExplainer to study the impact of 3D chromatin structure on DSB is novel, more details should be provided on the methods description, and additional experiments should be performed to establish that the FaCIN learnt by the model are biologically meaningful. See below for detailed comments.

Major comments:

1. Method descriptions: while the algorithm details of GNN and GNNExplainer is well described, detailed description of the preprocessing of Hi-C is missing. Preprocessing of Hi-C data is crucial to remove biases and to identify real contact events. This step is thus crucial for the model to learn meaningful connections associated with DSB. However, the authors provided zero description of the preprocessing of the Hi-C data. Did the authors use raw or preprocess contact map? How is it normalized? At what cutoff is a contact considered an edge in the graph? This information is important for a fair evaluation of the model performance, and whether the features the model learnt are biologically relevant, or just systematic biases in Hi-C experiments.

In addition, the following information are also missing from the Methods section:

- details of all statistical tests being performed.

- in the section of characterization of neck interactions, authors mentioned "we detected E-P loops", how are E-P loops detected. Are enhancers defined based on using histone marks?

- how is "topological subgraph search" performed?

2. Benchmarking: the main contribution of this manuscript is building a DSB prediction model by incorporating 3D chromosomal information. And the focus is on how 3D interactions contribute to DSB. Thus, it is important to establish that by incorporating 3D information, the model can predict DSB with improved performance. An important benchmark would be comparing the GNN model to a model that predicts DSB from the same set of features (CTCF, DNase and k-mers), but without Hi-C information.

3. the authors should provide more evidence that the FaCIN reveals a bottleneck pattern. The authors observe that while each prediction site has on average 91 direct interactions, only 1.6 out of top FaCIN interactions are direct. 91 and 1.6 are not comparable, since author mentioned in the Methods that for FaCIN, "we limit the (FaCIN) graph size to be max 10 top ranking edges …". Thus, the comparison in Fig. S3 is misleading. A fair comparison should be comparing (# direct interaction / # total interaction) for each prediction site to 1.6/10 in FaCIN. A statistical test should be performed to suggest whether FaCIN is enriched/depleted of direct interactions.

4. Questions regarding the features used for GNN model. Why use kmer as features? Why not use convolutional filters as feature extractors as used in many deep genomic models? 3,4,5-mers used in this study can lose information on local positional dependency, Which longer k-mers or CNN kernels can better captures. Peak number is not a good estimator of local accessibility, it is sensitive to the smoothing parameter of the peak caller. A sequence of small peaks can be called as a "broad peak", and similarly sometimes broad peaks can be split to smaller peaks. Especially for DNase, having a very broad peak versus a single narrow peak have very different meaning for the accessibility of the bin, while both will have a peak number of one. The authors should provide more justification for their choice of features.

5. The paragraph on "motif search" is confusing. The authors discussed "motif analysis" in terms of "motifs, such as transcription factor binding site and regulatory network motifs", but the analysis is on topological subgraphs. A TF motif search would involve performing TF motif enrichment analysis using tools like FIMO or Homer to look for enriched PWM at DNA sequences. The author looked for enriched topological subgraph, which is not related to TFs. The authors also didn't explain their approach in the Methods, or the biological relevance of the top hits presented in fig. 2c or additional file 2. Why does the presence of "forward chain" and "binary-parallel" suggest that "FaCIN contains the universal building blocks for chromatin organization at the interaction level"?

Minor comments:

1. GNNExplainer assigns importance scores to different input features. The authors only focused on CTCF and DNase features. What about the other k-mer features. Any explanation for the top k-mer features? Do they match any known sequence patterns such as TF motifs?

2. Fig3a, Fig.3d, and Fig.S4 need better legends. Details of statistical test should be better explained. For example, in Fg. 3a what are the boxes and points? What is the statistical test being used?

3. "CTCF and DNase signals were significantly higher at neck neighbors …", what are their importance score with GNNExplaner?

4. There's only 1.6 neck interaction per bin, but the focus of the analysis is only on neck interactions? Can the authors also discuss the rest of the FaCIN interactions?

5. Citation 18: formatting error.

**Authors Response**

**-------------------------------------**

**Response to Reviewer #1**

**-------------------------------------**

**Reviewer #1 (Remarks to the Author):**

**General comment:**

*In this manuscript, the authors examined the potential associations between the 3D genome structural features and DNA double-strand breaks (DSBs) by proposing a GNN-based and interpretable framework. The idea of implementing GNNExplainer into this framework is innovative and promising. The FaCIN unit and its bottleneck pattern are interesting and may be meaningful for further study. Please find the following detailed comments.*

**Response**: Thanks for the reviewer to evaluate our work to be innovative and promising. All the suggestions and questions are valuable and help improve the overall quality of this work. According to the major issues raised below, we have added the required analysis. Our detailed responses to the comments are as follows (changes in revised manuscript are marked in red).

**Major comments:**

1. *Major*

*As the authors described, they proposed a framework that integrates deep graph neural network (GNN). However, a general limitation of GNN is its layers cannot be too deep, which is well-known as the over-smoothing issue. The authors should provide more details in model construction, and it is also important to discuss how they deal with the over-smoothing issue in their framework.*

**Response**: As the reviewer pointed out, over-smoothing is a classical problem with GNNs.

The basic idea of over-smoothing is that after several iterations of message passing and aggregating of each GNN layer, the embeddings for all the nodes can become very similar to one another. However, this issue can be alleviated by *vector concatenations*, *skip* or *residual connections*, which try to directly preserve information for a node from its previous rounds of message passing, so that the updated node representations would not depend too strongly on the message aggregated from the neighbors [*W. L. Hamilton, "Graph representation learning", Synthesis Lectures on Artificial Intelligence and Machine Learning, vol. 14, no. 3, pp. 1-159*].

In our model DSB-GNN, how we prevent over-smoothing issue with above strategies is thoroughly illustrated in methods *DSB prediction model* section. The model can leverage longer-term dependencies in the graph while avoiding generating over-smoothed node embeddings. In our revised manuscript, we have added the corresponding information.

1. *Major*

*As the authors described, FaCINs is identified as a new unit for understanding DSBs. DNA lesions are important issue and DSB is a key type of DNA lesions. It is necessary to compare FaCINs with known DSB elements and further provide more insights, for example, how FaCINs associated with DSB repair.*

**Response**: Thanks for this comment. Our previous manuscript has adopted loops and TADs, the well-studied chromatin structural units, to make a comparison with FaCINs. As the reviewer suggested, we have compared FaCINs with more known DSB-related findings.

**For example**, reported by Canela, Andres et al [*Cell (2017). PMID: 28735753*]: DSBs are often introduced to release the torsional stress from continues cell activities like transcription and replication, TOP2B transiently break and rejoin both DNA strands (see the *Fig. 2 for reviewer*). But at an unknown frequency, TOP2B can fail to rejoin the broken strands, leading the two physically interacting genomic regions turn to one has TOP2B-induced breaks and the other is intact. Likewise, in FaCIN, we observed that most (nearly 80%) of the neck neighbours of DSB sites are largely non-DSB.


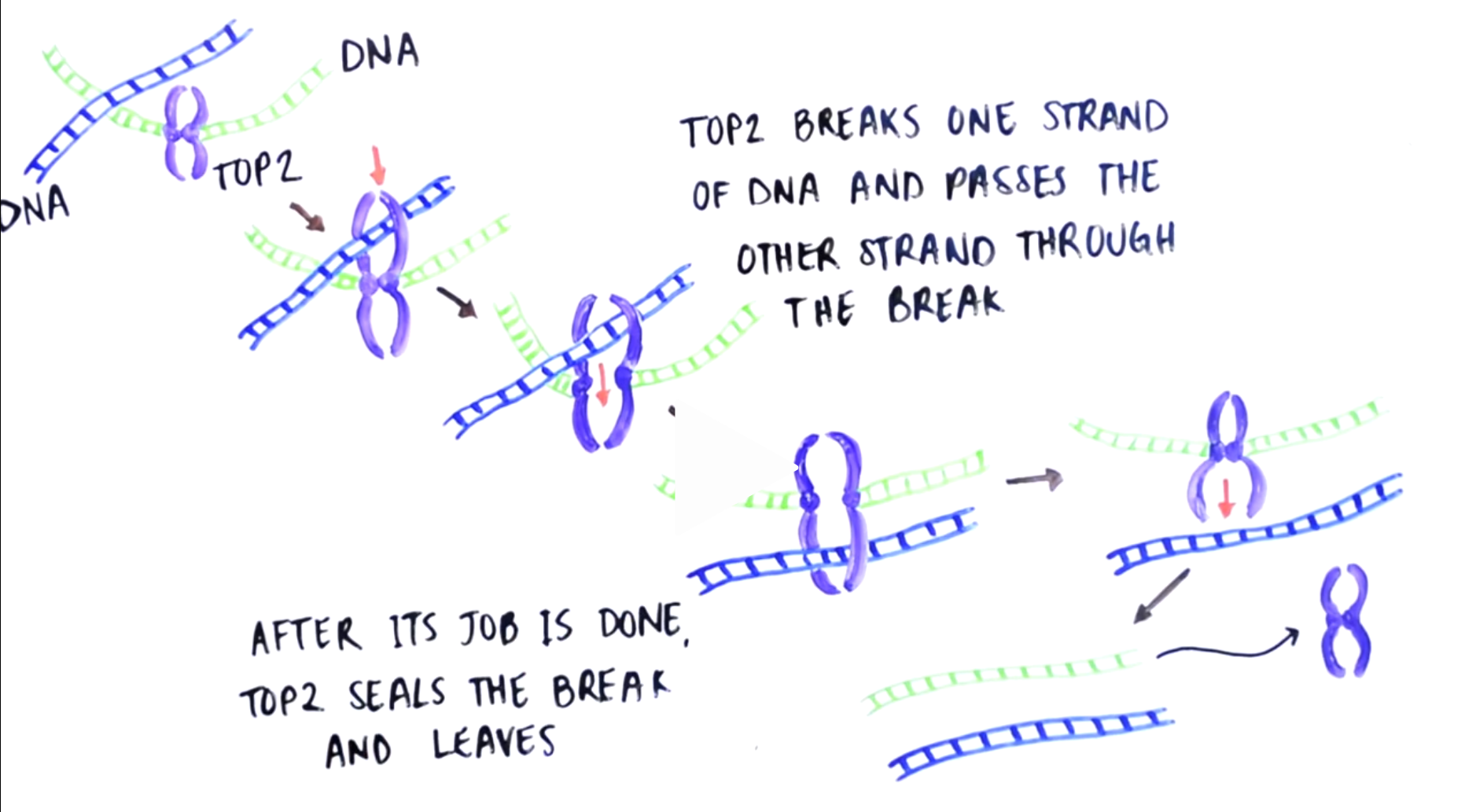


**Fig. 2 for reviewer.** A snapshot from the video abstract from

work of Canela, Andres et al.

About the relationship between FaCINs and DSB repair, it is reported that DSB positioned more closely are more likely to interact, and in FaCIN, we observed that neck neighbours of DSB sites are more likely to be DSBs (Fig. 4d in manuscript). This is might due to that the closely positioned DSBs cannot access the repairing factors hindered from a same chemical block or compact chromatin [*Scully, Ralph et al. Nature reviews. Molecular cell biology. (2019)*], indicating the role of structural template for FaCIN to coordinate different elements in DSB-related pathways.

We have added above content in our revised manuscript.

1. *Major*

*The biological meanings of "the 1242 loops with DSBs on one anchor and another linked by neck interactions" (Line 193). Further analysis is needed.*

**Response**: Thanks for this suggestion. Given previously reported that DSB contact neighbouring loci within a damaged TAD is a proper response，we speculate that these loops coinciding with neck interactions might contain information relevant to DNA-damage response. In such case, if a loop undergoes DSB at its anchor, its potential function might also be affected due to impaired loop extrusion, leaving traces on the nearby genes. Thus, we further looked into these 1242 loops and found that over 800 of them had one or both anchors coinciding with gene promoters.

We have provided this information in attached file named *file_5_for_referees-Loops_coinciding_with_neck_interactions.xlsx.*

1. *Major*

*I suggest the authors analyze the size of FaCIN, that is, calculate the distance between FaCIN node and its one-hop and two-hop neighborhoods, and examine the DNA elements in that genomic region. This may be important for gene regulation and even DSB repair.*

**Response**: Thanks for this comment. As the reviewer suggested, we analyzed the size of FaCINs as shown in *Fig. 9 for reviewer*. In FaCIN, the median length from prediction site to its 1-hop neighbour (i.e., neck neighbours) and 2-hop neighbours are on average 160-kb and 410-kb. Typically, proximal interactions are referred to those with genomic distance < 10-kb and distal ones are those ≥10-kb [*Holgersen, Erle M et al. Nature protocols (2021)*]. From the figure below, we can learn that **FaCIN includes both distal and proximal interactions and it is composed largely of** **those distal with rich 3D information, rather than mere proximal interactions** more due to linear genome proximity.


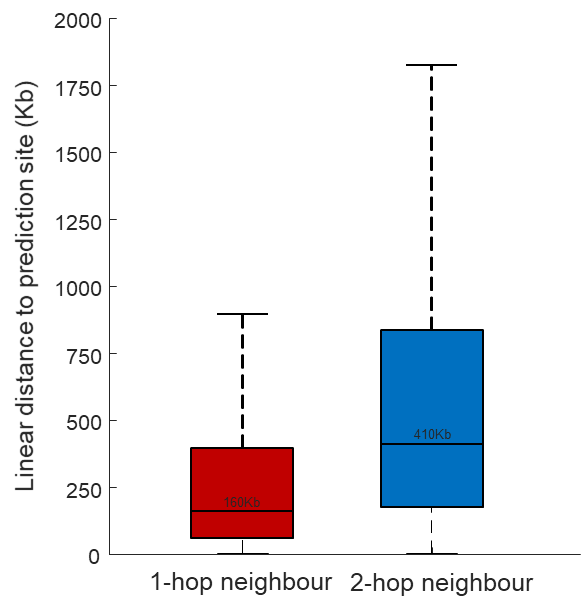


**Fig. 9 for reviewer.** FaCIN’s size in terms of distance from prediction site to 1-hop and 2-hop neighbours.

Besides, as the reviewer suggested, we also examined the DNA elements in genomic region lying between prediction site and neck neighbours or 2-hop neighbours.


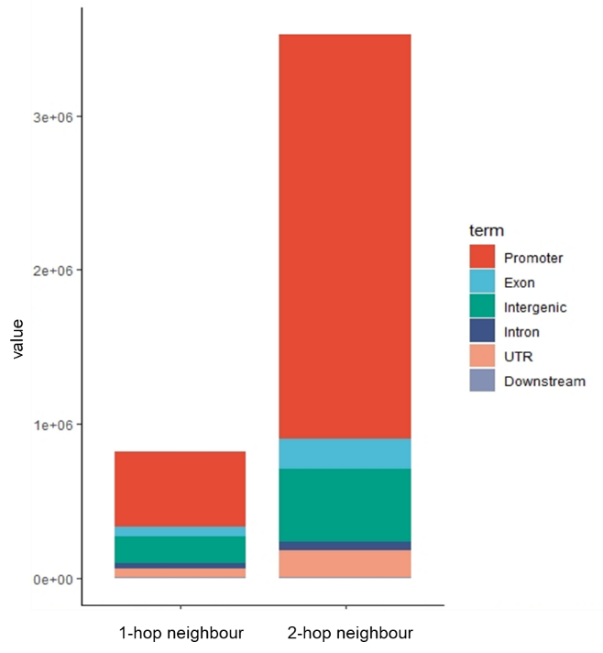


**Fig. 18 for reviewer.** Annotation for genomic regions between prediction site and 1-hop neighbour or 2-hop neighbour.

**Specific Comments:**

1. *What* *hypothesis tests the authors used are missed, Line 170, 194, Fig. 4d.*

**Response**: Sorry for the missing information of hypothesis tests. We have further gone through the manuscript with due care and complemented other similar omissions.

1. *Fig. 4e, the y-label, "Dnase signal" should be "DNaseI signal" or "DHS signal".*

**Response**: Sorry for our careless spelling. We have corrected it as DNase I signal.

1. *Fig 1.a, the number of 3-mer seq feature is 64 but not 256.*

**Response**: Thanks for the kind reminding. We have corrected it.

**-------------------------------------**

**Response to Reviewer #2**

**-------------------------------------**

**Reviewer #2 (Remarks to the Author):**

**General comment:**

*In this work, Sun et al. described a GNN-based method for predicting double-strand break (DSB) sites based on Hi-C contacts, sequence features, and chromatin (accessibility and CTCF binding) signals. The authors also used a method called GNNExplainer to interpret the models and identify features important for the predictions, based on which the concept of DNA fragility-associated chromatin interaction network (FaCIN) was proposed and some analyses related to it were performed.*

**Response**: From all the constructive comments and suggestions, we thank the reviewer for his/her superior expertise. We do appreciate the time and energies that the reviewer poured into our manuscript. According to following comments, we have reflected on how to present the whole model and results while avoiding a descriptive impression. Obviously, we did not balance it well.

To address the concerns, we have added necessary experiments as well as the analysis required by the reviewer. Our detailed responses to the comments are as follows (changes in revised manuscript are marked in red):

**Major comments:**

*1. No strong justifications are provided for the use of complex GNN in predicting DSB sites:*

***a.*** *If the purpose is to how that the prediction performance is improved, comparison with other existing methods and* *ablation studies (**with features only, with network structure only, with subsets of features only, without the self-attention mechanism, etc.) are needed to show the amount of improvement achieved.*

***b.*** *If the purpose is to provide biological insights, the final conclusions of the study, such as enrichment of DSB sites at loop regions and accessible chromatin, seem not particularly novel.*

***c.*** *Even with the interpretation method and the FaCIN concept, the manuscript offers limited insight into the general features of DSB sites. For instance, the authors have not explained how the chains and bifurcation motifs are generally related to DSB.*

**Response**:

Many thanks for this comment. It reminds us that we did not offer a good motivation to build this model, or enough explanations to establish the biological roles of FaCIN or neck interaction. **Our purpose is to provide biological insights.** In our revised manuscript, we have devoted more space to illustrate why we design this model, and informed the biological relevance of FaCIN and neck interaction.

**For comment a**: **Yet we were not mean to merely provide another tool** with higher prediction performance, **we agree with the reviewer’s suggestions that comparison with other methods will help to improve our work**. To achieve this:

First, we trained LightGBM [*Ke, Guolin et al. NIPS (2017)*] and Random Forest (RF) [*Breiman, L. Machine Learning (2001)*], two widely used methods to benchmark deep learning works. We also performed ablation experiments on LightGBM, RF and our model. Results in *Table 1 for reviewer* show that our proposed model DSB-GNN consistently outperformed LightGBM and RF across different sets of features. Besides, the integrated features brought a boosted performance for all three methods.

**Table 1 for reviewer.**

| Feature | **DSB-GNN**  AUC | **LightGBM**  AUC | **RF**  AUC |
| --- | --- | --- | --- |
| 3-mer | 0.7620 | 0.7298 | 0.6100 |
| 4-mer | 0.7700 | 0.7321 | 0.6184 |
| 5-mer | 0.7756 | 0.7256 | 0.6153 |
| k-mer | 0.7857 | 0.7331 | 0.6499 |
| DNase | 0.8865 | 0.8668 | 0.6984 |
| CTCF | 0.8531 | 0.8245 | 0.6751 |
| k-mer +DNase +CTCF | **0.9251** | 0.8862 | 0.6598 |

Second, we investigated two recently proposed methods dedicated to DSB prediction. One of them [*Mourad, Raphaël et al. Genome biology (2018)*] reported a Random Forests-based approach and achieved AUC = 0.97. However, this RF approach used far more features for prediction including DNase, CTCF, H2AZ, H3K4me1, H3K4me2, H3K4me3, H3K27ac, DNA motifs and DNA shape. These features, especially the epigenetic marks, are not equally available for general cell lines or tissues, which greatly hinders the application. Another method [*Ballinger, Tracy J et al. Genome biology (2019)*] used 21 features including DNase, POL2B, CTCF, histone modifications, replication timing, RNA-seq and DNA secondary structures. Instead of using AUC, this method used Pearson’s r as performance metric to evaluate the predicted DSB frequency and achieved r = 0.92.

Together, our model achieved comparative performance using fewer features. More importantly, it provides a more systematic view to study DSBs leveraging chromatin structural information, which distinguishes from other methods in both research perspectives and application scenarios. Given that our purpose is to provide biological insights of DSBs, this performance ensured us to further explore DSBs.

**For comment b and c:** We much appreciate this constructive criticism and we take this comment seriously. The enrichment of DSB at particular regions was not our final conclusions but the intermediate supporting arguments. The reviewer has awakened us to that our work was not clearly organized and our most treasured focus and novelty was not conveyed as completely it should have been. We here give the key points as follows.

**First**, **our effort to present those already reported findings** (such as DSB sites tend to occur at loop anchors and accessible chromatin regions) is not to make a repetitive conclusion, **but to illustrate that FaCIN is able to cover the current biological knowledge and thus is rooted in a biologically-reasonable ground**. That is, the consistency between our findings with those previously reported supports

**the validity of FaCIN, paving the way to further analysis for FaCIN**.

**Second**, **our highlights are FaCIN and its bottleneck pattern**. Unlike loop or TAD, FaCIN does not characterize any distinct genome regions with significant chromatin interactions, it aims to reveal **a universal form of how the** **fragility of a piece of DNA might be affected (or affect) by the whole genome in three-dimensional space**. **In brief,** **FaCIN helps to truly dissect the mechanisms driving DNA fragility under the 3D genome.**

Though considerable progress has been made, previous works focus on different aspects such as transcription, phase separation, DSB repair system, and DNA mobility in the context of DNA damage. These works provide only scattered knowledge and sometime produce ideas contradicting each other. For example, the histone modification H3K36me3, which is present along active transcription units, is thought to promote homologous recombination (HR), a major DSB repair pathway; paradoxically, H3K9me2/3 heterochromatin, which packages transcriptionally inactive regions of the genome, has also been implicated in promoting HR [*Schep, Ruben et al. Molecular cell (2021)*].

This is very likely due to that these works each offers only a glimpse of DSB from different aspects. Besides, these works only resolve the relationship between factors of their interest with DSB in a pairwise manner but cannot deal with hierarchical relationships of higher dimension (see the *Fig. 1 for reviewer*), while this hierarchical relationship is exactly what encodes much more information.


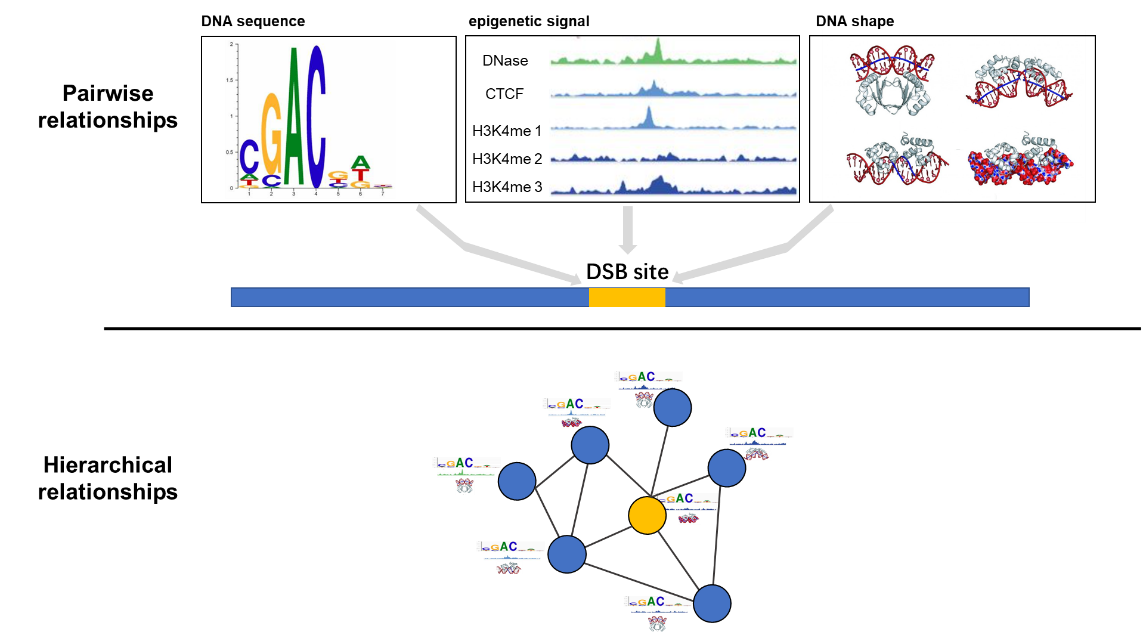


**Fig. 1 for reviewer.**

To overcome above limitations, **3D genome is a promising solution as it can organize those aspects into a systematic view, and it is fundamental enough to cover the various DSB-related events**. Computationally, it is not easy to cope with the hierarchical relationships from whole genome scale, except for GNN plus interpretable technique, of which the combination has achieved tremendous success. In our work, leveraging GNN and GNNExplainer, **we transform Hi-C data into a graph** where node represents genome bin and edge represents chromatin interactions. Therefore, **the epigenomic information, DNA shape, sequence preference, spatial proximity and other factors can always be attached to either the genome bin or the interaction**.

**Third,** through this way, **our work provides a more biologically informative model and allows a settlement for different phenomena with reasonable explanations**.

**For example**, it is reported that DSB positioned more closely are more likely to interact, and in FaCIN, we observed that neck neighbours of DSB sites are more likely to be DSBs (Fig. 4d in manuscript). This is might due to that the closely positioned DSBs cannot access the repairing factors hindered from a same chemical block or compact chromatin [*Scully, Ralph et al. Nature reviews. Molecular cell biology.(2019)*].

**Another example** is reported by Canela, Andres et al [*Cell (2017). PMID: 28735753*]: to release the torsional stress introduced by continues cell activities like transcription and replication, TOP2B transiently break and rejoin both DNA strands (see the *Fig. 2 for reviewer*). But at an unknown frequency, TOP2B can fail to rejoin the broken strands, leading the two physically interacting genomic regions turn to one has TOP2B-induced breaks and the other is intact. Likewise, in FaCIN, we observed that most (nearly 80%) of the neck neighbours of DSB sites are largely non-DSB.


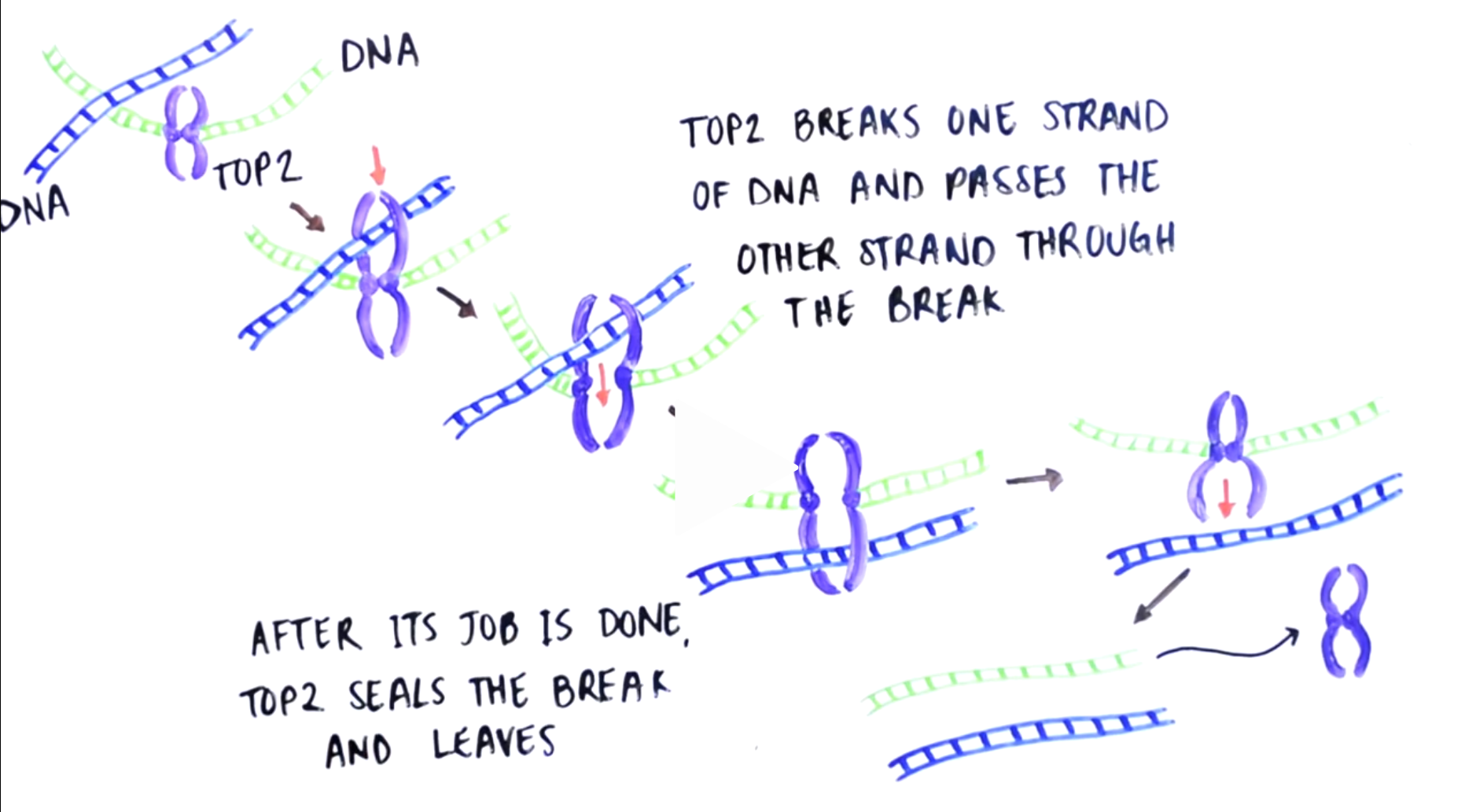


**Fig. 2 for reviewer.** A snapshot from the video abstract from

work of Canela, Andres et al.

Given above points, we respectfully request a reevaluation of our work’s novelty and we would like to provide further discussion. As rewritten parts corresponding to this comment are dispersed in introduction, results and methods sections and therefore not listed here. Please review the revised manuscript.

*2. Regarding "bottleneck" and "neck interactions":*

***a.*** *The key concept of "bottleneck" is not formally defined. The fancy illustrations in* *Figure 2a and Figure S2 do not help understand exactly what a bottleneck is, mathematically or in terms of genome architecture.*

***b.*** *The term "neck interaction" is quite confusing. According to the description in the manuscript, "for a given genome bin of interest, we refer to this genome bin as prediction site. FaCIN of this prediction site consists of interactions and each of them belongs to neck interactions or other interactions, according to whether it directly connects the prediction site." -- So, no matter which genomic bin is taken as the prediction site, its direct interactions in the FaCIN are always called neck interactions?*

**Response**: We are sorry that our previous representation failed to provide a clear illustration of above concepts. We have rewritten the corresponding parts and the responses are as follows.

**For comment a**: **We borrow the idea of bottleneck purely because this word refers to a most slender part of the typical shape of a bottle.** The bottleneck is just used to describe the topological pattern of FaCIN for visualization purpose. To describe what a bottleneck is, we provide more detailed explanations from both genome architecture and mathematic views.

**
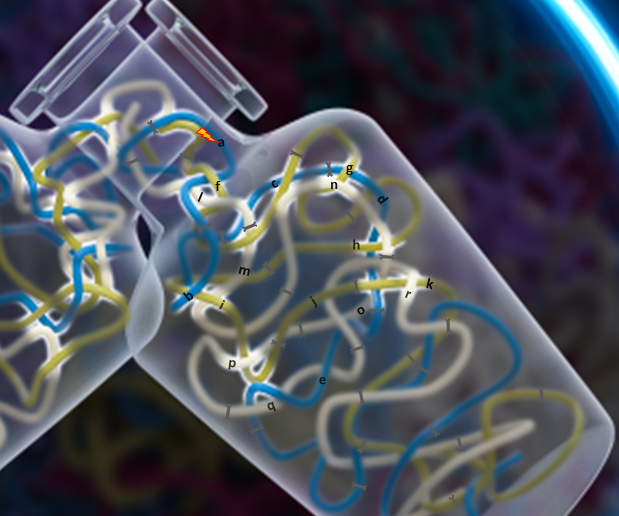
First**, under the view of 3D chromatin structure, FaCIN was identified as a group of 5-kb genomic bins and interactions. These genomic bins and interactions are crucial in DSB prediction and together formed a bottleneck-like structure. We take *Fig. 3 for reviewer* for a clearer explanation:

**
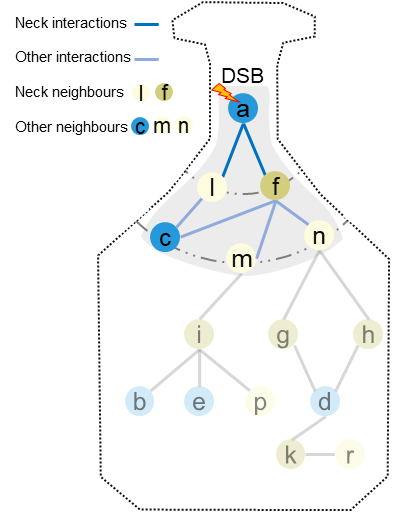
Fig. 3 for reviewer.** More details for FaCIN’s bottleneck pattern.

**The left figure provides a visual description of the bottleneck pattern where:**

- **Tangled lines** represent the intricate folding of chromatin, and different line colors are used for better distinction.
- **Highlighted regions** represent the physical contacts between pairs of genome bins.
- **Grey short thick lines** are used to mark consecutive 5-kb genome bins.

Look into the bottle, take the bin noted *a* as the prediction site, if we read along the chromatin in blue, we will find one by one the genome bins noted as *a*, *b*, *c*, *d*, *e* and so on. Likewise, the olive one consists of the bins from *f* to *k*; the off-white one consists of the bins from *l* to *r*.

**The right figure is a schematic graph of the left one where:**

- **Nodes** correspond to the genome bins in the left.
- **Edges** correspond to the highlighted physical contacts in the left.
- **Subgraph with a light grey background** is the FaCIN of node *a.*

In this figure, node *a* first associates with nodes *l* and *f*, while *l* and *f* separately associate with nodes of fast increasing number. It extends like a tree crown: direct interactions only account for a relatively small number, while the subsequent interactions form a pile of branches. These two parts contrastively form a topological shape really suggestive of a bottle. We have rewritten the corresponding parts for legends of Fig. S2.

The “bottleneck” concept aims to visually describe FaCIN and by examining this pattern, we identified the neck interaction which help uncover the intimate genomic elements for fragile sites.

**Second**, in mathematics, a famous theorem on bipartite graph named Hall’s theorem uses bottleneck to refer to a particular set of nodes. Briefly, as shown in *Fig. 4 for reviewer*, let G be a bipartite graph with vertex partitions L and R. There is a matching in G that covers L if no subset of L is a bottleneck. Here, the bottleneck means for a set S if its elements |S| > its neighbours |N(S)|, the set S is called a bottleneck. This point is often used as a proof by contradiction like below. (Hall’s theorem)


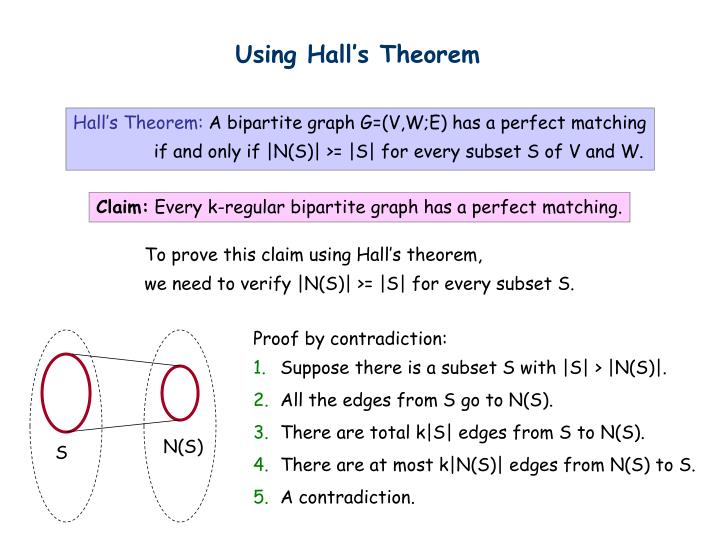


**Fig. 4 for reviewer.** Sketch for the bottleneck in proof using Hall’s theorem.

Our bottleneck pattern has a similar extended meaning to that of Hall’s theorem, which slightly differs in that the neck interactions have less elements than neighbours.

**For comment b**: **Yes**, just as the reviewer pointed out: “So, no matter which genomic bin is taken as the prediction site, its direct interactions in the FaCIN are always called neck interactions”. Neck interactions are those interactions directly contact with the prediction site (any genome bin of interest).

We understand that this answer might raise further concerns like: “So, given that almost every genome bin has its own neck interactions (except those isolated bins), are the neck interactions still important? In what sense?” or rather “Wouldn’t the ubiquity of neck interaction impair its importance?”.

Thus, we next explain why it, being the neck interaction or being the FaCIN, is still important, despite that it exists for almost every genome bin:

**First**, GNNExplainer [*Ying, Rex et al. Advances in neural information processing systems. 2019*], as a GNN interpreting technique, has been applied into many tasks across different fields, such as predicting the mutagenic effect of a given chemical molecule graph and discerning the Question-Answer (QA) interactions from Online-Discussion interactions. The following *Fig. 5 for reviewer* is taken from the original work of GNNExplainer, which briefly summarizes the learnt crucial structure of GNNExplainer to make decision. With the strong power of GNNExplainer, one can tell that the crucial structure quite matches the nature of corresponding tasks. For example, the ring structure in mutagenic effect prediction task and the topic reactions in QA task all well match to the prior knowledge.


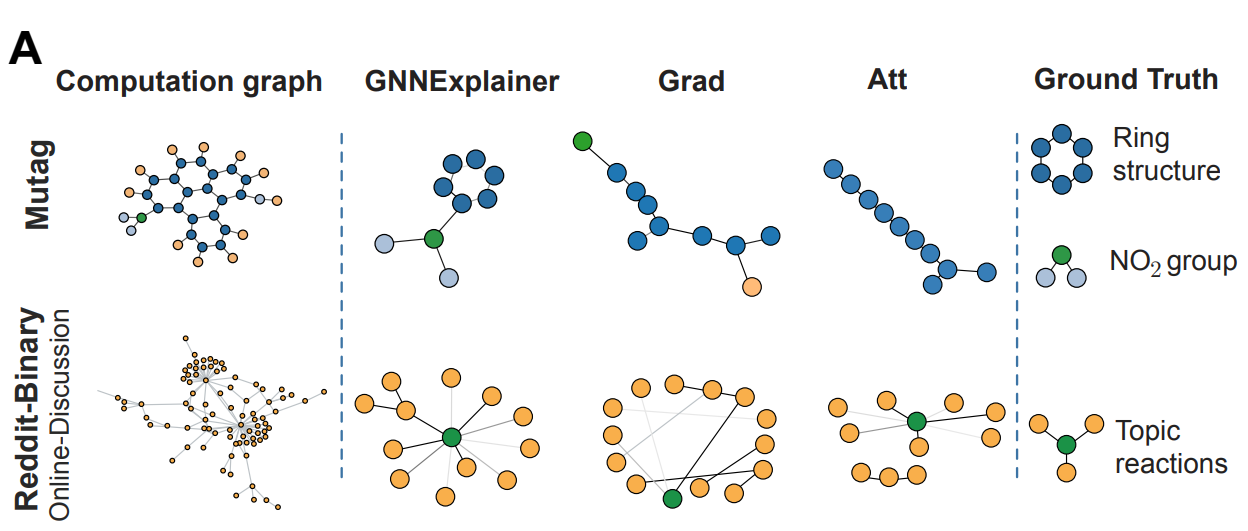

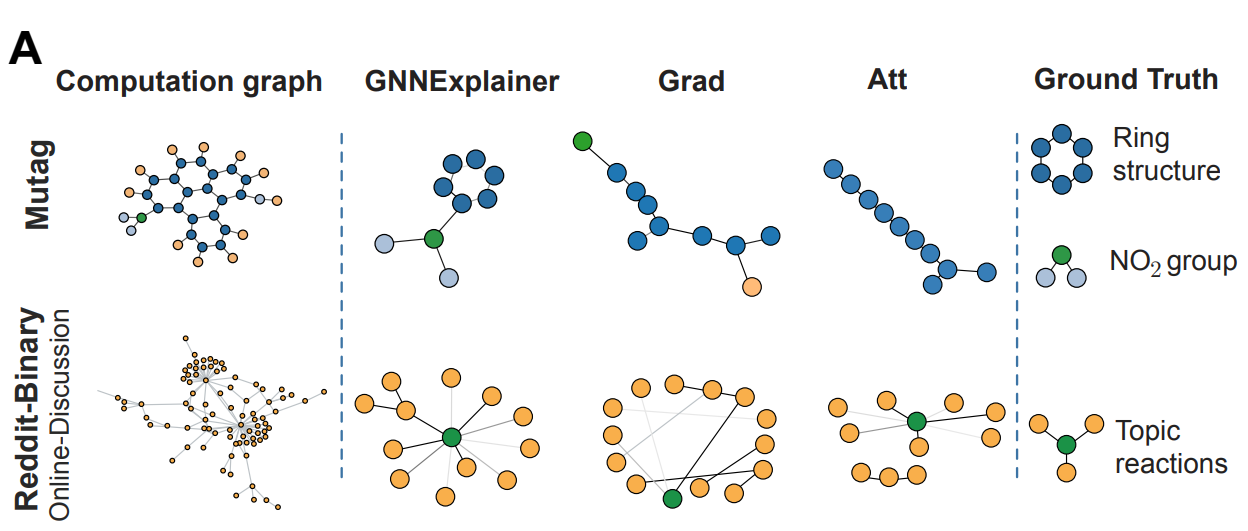


**Fig. 5 for reviewer**. An example to illustrate how GNNExplainer makes decisions.

Top: mutagenic effect prediction. Bottom: forum discussion classification.

**Above findings from other similarly complex domains can also illuminate and inform the importance of the enriched motifs in FaCIN.** Likewise, by implementing GNNExplainer in this DSB-related task, we identified the bottleneck-like FaCIN, and we believe this pattern as well as its neck interactions are of biological importance for DNA fragility.

**Second**, different from loop or TAD, FaCINs are not any distinct genome regions that have significantly higher number of chromatin interactions. Actually, FaCIN is proposed to help reveal a universal form of mutual interaction under the DSB context, that is, how each region (e.g., of size 5-kb) might be affected by the whole genome when we are concerned with its DNA fragility. Therefore, it is about something more “consensus”. By analogy with cloth, FaCIN shows the fabric or texture that depends on how the threads are woven together, but not the pattern or symbol on the cloth.

In summary, FaCIN is of biological importance for DNA fragility and its ubiquity does not impair its importance.

*3. The authors claim that they have used "deep graph neural network" in their work:*

***a.*** *However, it seems that in the whole manuscript the number of GAT layers used is not stated.*

***b.*** *It has been reported that having more GNN layers does not necessarily lead to better performance, sometimes even worse beyond 2-3 layers. It is therefore not clear why the authors advocate the "deep" nature of their network.*

***c.*** *The authors have not compared the prediction performance with different numbers of GNN layers to justify the need for a "deep" network.*

**Response**:

We merge the responses to above three questions into what follows. Our model consists of **three GAT layers** which should never be called “deep”. Sorry that we have used an inappropriate phrase. The claims like “deep GNN” have appeared in our manuscript three times and one of them is even in the title. We failed to chew over the three “deep” left in the main text but only focused on the methods section for model illustration. As can be seen from methods, we mainly highlighted our design for each GAT layer but did not emphasize that the model is unusually deep or has any advantage in its depth. We do apologize for this and have corrected this mistake in revised manuscript. We also attached the code for model construction as a file named *file_1_for_referees-Code_for_model_construction.py*.

*4. It is not certain from the manuscript how much the results are affected by Hi-C read depth and data resolution (i.e., bin size):*

***a.*** *The robustness of the method with respect to these parameters have not been evaluated.*

***b.*** *The NHEK Hi-C data set used does not have a high read depth, and therefore it is doubtful whether using 5-kb bin size would lead to too much noise and sparsity.*

**Response**: We thank the reviewer for rigorous thinking and constructive suggestions about the robustness of our model. In most Hi-C related studies, the selection of the resolution should match the read depth to avoid introduce too much noise and sparsity. This match is necessary for downstream analysis such as calling loops or TADs which involves identifying interactions with significantly higher contacts. Here, in this study, unlike loops or TADs, FaCIN focus on revealing a universal form in terms of chromatin interaction and therefore does not require to filter significant interactions. In contrast, what required by FaCIN is exactly the raw data as global as possible. The 5-kb bin size is used as a window to examine DSBs instead of to discern new loops or TADs. Despite that noise and sparsity problems might exist, they are solved actually within the DSB-GNN model. Nonetheless, we agree that to evaluate the robustness of our model against the mentioned parameters will further polish this work.

To address this concern, we evaluated our model against different **read depth**, **Hi-C resolution**, and different **Hi-C normalization**. Briefly, the data was first down-sampled into 4 subsets ranging in size from 20% to 100% of the initial sequencing depth. Each of the subset was then retained (raw) or normalized with KR or ICE. Then the datasets were further binned with different size of 5-kb, 10-kb and 25-kb. *Fig. 6 for reviewer* shows a summary of the data processing.


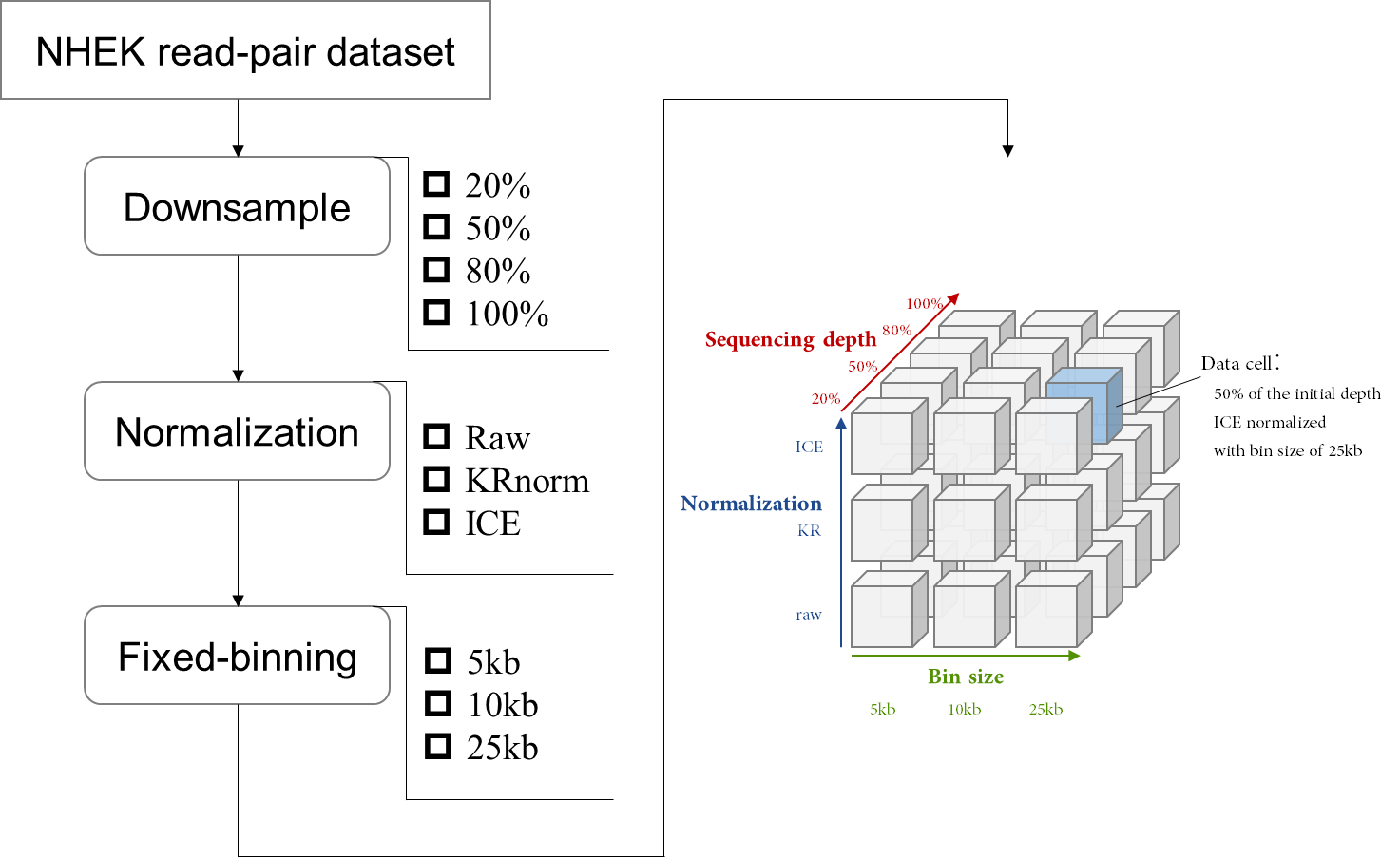


**Fig. 6 for reviewer.** Processing workflow to generate data of different settings.

First, we performed 80%, 50% and 20% down-sampling on Hi-C data of NHEK cell line. From the following statistics of the used NHEK Hi-C dataset (see *Table 2 for reviewer*, lower left), we can learn that reads number after down-sampling ranges from 132 million to 529 million, which covers a considerable range of Hi-C datasets (for example, the data gathered by 4DN Data Portal (see *Fig. 7 for reviewer*, lower right), summarized from https://data.4dnucleome.org/hic-data-overview).

| **Table 2 for reviewer.** | |
| --- | --- |
| Total Reads | 1,073 ,206,855 ≈ 1b |
| Filtered reads | 662,762,700 ≈ 663m |
| Total Contacts | 644,899,299 ≈ 645m |


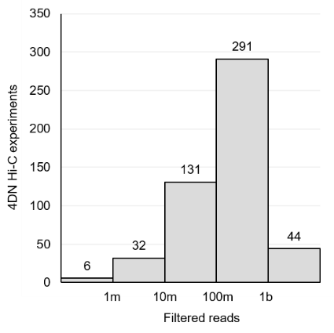


**Fig. 7 for reviewer.** Distribution of filtered reads for Hi-C data from 4DN Data Portal.

Then, we generated Hi-C raw contact map with different resolutions of 5-kb, 10-kb and 25-kb and we performed KR and ICE normalization on above Hi-C data.

Detailed results of DSB prediction are showed in *Table 3 for reviewer*.

| **Table 3 for reviewer.** | | Hi-C resolution | | | |
| --- | --- | --- | --- | --- | --- |
| Normalization | **RAW** | 5-kb | 10-kb | 25-kb | |
| Read depth | 20% | 0.8611 | 0.8670 | 0.8864 | |
|  | 50% | 0.8721 | 0.8816 | 0.8977 | |
|  | 80% | 0.9023 | 0.9015 | 0.9012 | |
|  | 100% | 0.9251 | 0.9020 | 0.9006 | |
|  | | | | | |
| Normalization | **KR** | 5-kb | 10-kb | 25-kb | |
| Read depth | 20% | 0.8658 | 0.8787 | 0.8807 | |
|  | 50% | 0.8790 | 0.8882 | 0.8929 | |
|  | 80% | 0.9023 | 0.9033 | 0.9014 | |
|  | 100% | 0.9228 | 0.9072 | 0.9023 | |
|  | | | | | |
| Normalization | **ICE** | 5-kb | 10-kb | 25-kb |  |
| Read depth | 20% | 0.8680 | 0.8711 | 0.8776 |  |
|  | 50% | 0.8775 | 0.8758 | 0.8878 |  |
|  | 80% | 0.9015 | 0.9007 | 0.8987 |  |
|  | 100% | 0.9222 | 0.9035 | 0.9008 |  |

Results showed that the predictive performance exhibited a moderate decrease (AUC from 0.9251 to 0.8611 on raw data) along with down-sampled Hi-C read depth from 100% to 20%, indicating the robustness of our model against data read depth. But it was not affected by different Hi-C normalization methods and this is not surprising because in our model the **normalization is no longer a preprocessing step but partly transferred to the GNN model**, as the graph weights derived from raw interactions are constantly updated during training. While for resolution, with sufficient read depth, low resolution slightly reduced the performance but it tended to in turn bring elevation for particularly insufficient read depth. This was natural since high resolution used in low read depth would introduce much noise and sparsity.

*5. Regarding the subset of Hi-C contacts used:*

***a.*** *The authors have not justified the omission of inter-chromosomal interactions.*

***b.*** *The authors have not carefully distinguished between proximal interactions more due to one-dimensional genomic* *proximity and distal interactions more due to three-dimensional genome architecture.*

***c.*** *The authors have not explained what quantity they have used to indicate interaction strength in the edge weights.*

**Response**: Thanks for reminding us to complete our preprocessing of Hi-C data as well as the methods details.

**For comment a**: Our reasons for not including inter-chromosomal interactions were two-fold.

First, the computation will not be easily manageable. Taking all inter-chromosomal interactions into account means that the adjacency matrix of graph will expand from chromosome-scale to genome-scale. Accordingly, the number of nodes will substantially increase (see *Table 4 for reviewer*) while pose a real challenge to computational time.

| **Table 4 for reviewer.** | |
| --- | --- |
| Configuration of local device   - GPU: Nvidia, 2080Ti - System: Linux 3.10 - Cuda: 11.2 | |
| Chromosome and node number | time cost |
| chr1: 43,717 | > 9 hours |
| chr 5: 34,956 | > 7 hours |
| chr10: 22,500 | > 5 hours |
| chr22: 6,587 | > 3 hours |
| Whole-genome:554,817 | \ |

Second, DSBs are widely dispersed across whole genome while inter-chromosomal interactions are much sparser than the intra-chromosomal ones, according to *Fig. 8 for reviewer* visualizing our used NHEK Hi-C data. The contact density for both intra- and inter-chromatin are calculated as below (*Table 5 for reviewer*). Intuitively, these interactions might only provide relatively limited information to help predict DSBs.


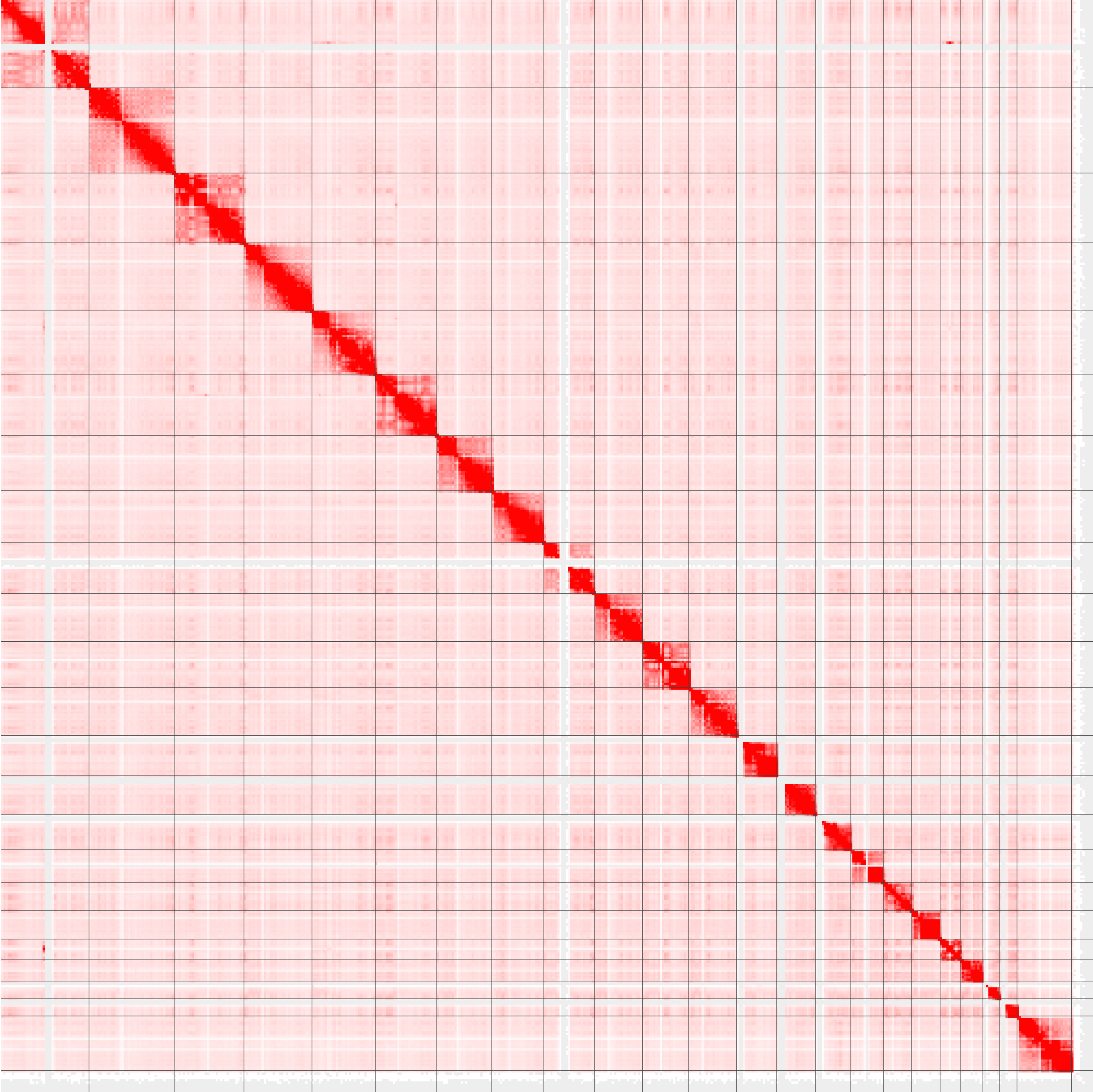


**Fig. 8 for reviewer.** The contact map of used NHEK Hi-C data.

| **Table 5 for reviewer.** | | |
| --- | --- | --- |
| contact density | Intra-chromosome | Inter-chromosome |
| Entry sum/Entry num | 0.0229 | 0.0013 |

**For question b**: Indeed, to distinguish between proximal and distal interactions is rather important for most Hi-C related studies, as the significance of an interaction might be distorted by linear proximity. While in our work, FaCIN is identified as a universal form of chromatin structural unit concerning with DNA fragility, which requires for the raw data as global as possible and does not exclude interactions according to significance. Therefore, we did not use any normalization to distinguish the proximal interactions from distal interactions. We next give more evidence to support this point from both **Hi-C data preprocessing** and **subsequent results analysis**.

**For Hi-C data preprocessing:**

We added experiments using KR- or ICE- normalized Hi-C data and the results indicated that additional normalization showed no signs of improvement for our model (please refer to the *Table 3 for reviewer* in above response to comment 4). This is not surprising as deep learning based models have exhibited the ability in many — though not all — tasks to remove the need of feature engineering. In our model, the **normalization is no longer a preprocessing step but partly transferred to the GNN model**, as the graph weights derived from raw interactions are constantly updated during training.

Therefore, given the overall trend that deep learning can deal with most tasks at least no worse than traditional methods, plus the performance of our model, we reason that a particular step to specially distinguish the proximal interactions from distal ones may not seem very necessary.

**For results analysis**, we analyzed the FaCIN’s size and results are shown in *Fig. 9 for reviewer*. In FaCIN, the median length from prediction site to its 1-hop neighbour (i.e., neck neighbours) and 2-hop neighbours are on average 160-kb and 410-kb.Typically, proximal interactions are referred to those with genomic distance < 10-kb and distal ones are those ≥10-kb [*Holgersen, Erle M et al. Nature protocols (2021)*]. From the figure below, we can learn that **FaCIN includes both distal and proximal interactions and it is composed largely of** **those distal with rich 3D information, rather than mere proximal interactions** more due to linear genome proximity. This also diminishes the need for distinguishing distal interactions from those proximal.


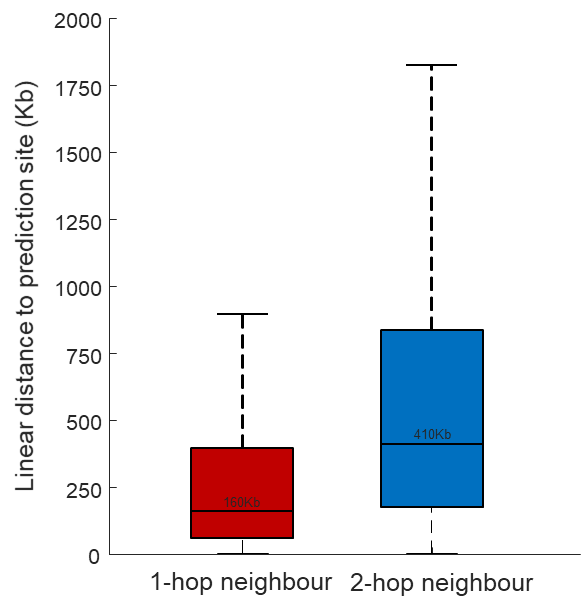


**Fig. 9 for reviewer.** FaCIN’s size in terms of distance from prediction site to 1-hop and 2-hop neighbours.

**For comment c**: We used the counts in raw observed contact maps as interaction intensity and we have updated the information in the revised manuscript.

*6. Regarding the GNNExplainer method:*

***a.*** *Exactly how masking is performed seems not clearly described. For instance, does it involve re-training the embedding and the classifier?*

***b.*** *Computationally, how feasible is it to mask each edge one by one to evaluate its importance?*

**Response**: Thanks for this comment.

**For question a**: sorry for the unclear description of GNNExplainer implementation. Briefly, the masking can be broken down into:

- The goal of masking : for given a node v, the basic goal of GNNExplainer is to identify a subgraph G_S_ ⊆ G, and the associated feature set X_S_ = { x_j_ | v_j_∈G_S_ } that are important for prediction of the node.
- How masking is performed: it is **achieved by** **matrix operations in an element-wise manner by following steps**:

**1.** According to an initial masking matrix, loss is calculated. Note that this loss is different from the training loss of DSB-GNN, it is used to measure to what extent the current masking matrix can satisfy the goal of identifying the optimum subgraph GS and feature set X_S_.

**2.** Under the guidance of above loss, the masking coefficients are adjusted.

**3.** Steps 1 and 2 are iterated until the conditions are met.

Specifically, the masking approach **does not involve the re-training** **for DSB-GNN model**.

**For question b**: It is a quite legitimate concern about computational efficiency. Mathematically, we transformed DSB prediction into a node classification task on graph. As the GNN-based model uses a layer-wise rule for both propagation and update for all nodes, for each prediction site, the edge masking only needs to be performed inside the scope most relevant to the prediction site rather than the whole-graph scale. In our work, each prediction was made actually by the integrated information from the region within N level of connections (known as hops) around the node, where N is the number of used layers. Despite a narrowed masking scope, to explain for every node really consumed some time. To provide more information, we here reported the computation resource and time cost (see *Table 4 for reviewer* above).

| **Table 4 for reviewer.** | |
| --- | --- |
| Configuration of local device   - GPU: Nvidia, 2080Ti - System: Linux 3.10 - Cuda: 11.2 | |
| Chromosome and node number | time cost |
| chr1: 43,717 | > 9 hours |
| chr 5: 34,956 | > 7 hours |
| chr10: 22,500 | > 5 hours |
| chr22: 6,587 | > 3 hours |
| Whole-genome:554,817 | \ |

All details of masking processes have been added to the Methods section in the revised manuscript.

*7. Different parts of the methodology, and correspondingly the results, may depend strongly on some threshold values:*

***a.*** *Threshold for two genomic bins to be considered interacting according to a Hi-C data set. For instance, the "neck interaction" shown in Figure 5b does not look like a strong interaction.*

***b.*** *Threshold for the amount of change in a prediction such that the omission of a Hi-C interaction is considered importantly affecting the prediction*

**Response**: Thanks for this comment and these are all legitimate concerns as we previously failed to clearly state that whether these results were obtained with threshold or not. Actually, **we tried to keep the model’s performance as independent as possible from threshold parameters**. For example, we did not use customized threshold values to identify loops, TADs, or genomic elements such as enhancers. We obtained them either from the original literature or from the widely recognized database.

**For comment a**: As earlier mentioned, FaCIN is identified as a universal form of chromatin structural unit concerning with DNA fragility, which requires for the raw data as global as possible. Therefore, **we only discarded the contacts of value ≤ 1** construct the adjacency matrix from Hi-C raw contact maps and it means we retained top 20% of contacts (See *Fig. 10 for reviewer*) and this is the only threshold in this work specified by us. **Therefore, the neck interactions** obtained in downstream analysis **are not necessarily strong interactions.** The neck interactions are determined by their importance to the prediction, which is a result of multi factors involving the topological location, the epigenetic features as well as the interacting strength. The contact value of “neck interaction” shown in Figure 5b is 2, which indicates a weak interaction. Besides, a classic study about network motifs [*Milo, R et al. Science (2002)*] reported that patterns that are functionally important but not statistically significant could exist, which otherwise would be missed if we perform an overly strict filtering.


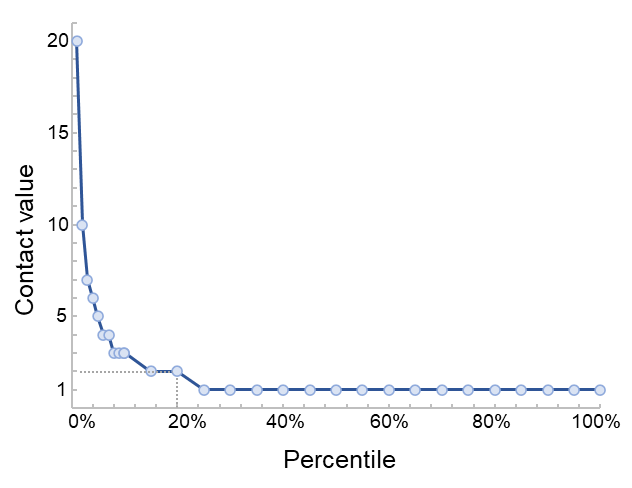


**Fig. 10 for reviewer.** Distribution of contact value in Hi-C raw contact maps.

**For comment b**: We appreciate for the reviewer’s keen insight into this point. During analyzing the explanation from GNNExplainer, how to measure the impact brought by a masked interaction is quite important. To make a threshold is a natural way, however it does not apply here due to limitations from two aspects. First, as mentioned earlier, the GNN-based model uses a layer-wise rule for both propagation and update for all nodes. In our work, each prediction was made actually by the integrated information from the region within three (the number of used layers) level of connections around the node. Therefore, we should restrict the FaCIN to be covered by the integration-involved area and otherwise the interactions might have no contribution even if they are above a specified threshold. Second, for a trade-off between computation cost and informative explanation, the upper limit of FaCIN’s interactions should be moderate. Therefore, we did not perform thresholding to distinguish the important interactions from the trivial ones and we just select no more than 10 edges from the top performers. Assume the changes brought by removing an edge can be quantified as ΔP, then the importance score for this edge is actually a function of ΔP. One interaction is considered as important for the prediction when its importance score is high enough to become one edge in FaCIN. We have rewritten the corresponding parts in the manuscript.

*8. "These motifs provided statistical evidence of the bottleneck pattern in FaCIN":*

***a.*** *In fact, the authors have not provided any statistical evidence that these motifs are enriched given the Hi-C contact map. They have only said that the "forward chain" and "binary parallel" motifs are most frequent, but they may also be most frequent in the Hi-C interactions in general.*

***b.*** *Even if the motifs are significantly enriched in the FaCINs, how this would provide statistical evidence of the bottleneck pattern is unclear.*

**Response**:

**For comment a:** Actually, as the reviewer pointed out, statistical analysis is required to evaluate the enrichment of FaCIN motifs. To address this, we generated **randomized graphs using Random Walk on raw Hi-C contact maps and they have the same overall characteristics as does the FaCIN**: a connected subgraph for a genome bin which covers at maximum a two-hop region with no more than 10 edges. Then, we **performed subgraph searching** on these randomized graphs and found the top subgraphs were quite different from those for FaCIN (*Fig. 11 for reviewer*). They account for patterns that appear only because the general Hi-C interactions, and their appearing frequencies are shown. In summary, the reported motifs are enriched in FaCIN rather than the general Hi-C interactions. We also attached above results on randomized graph in a compressed file named *file_2_for_referees-Subgraph_search_result_on_random_graph.zip*. Due to file size limitation, it only includes results from chr1-chr3.


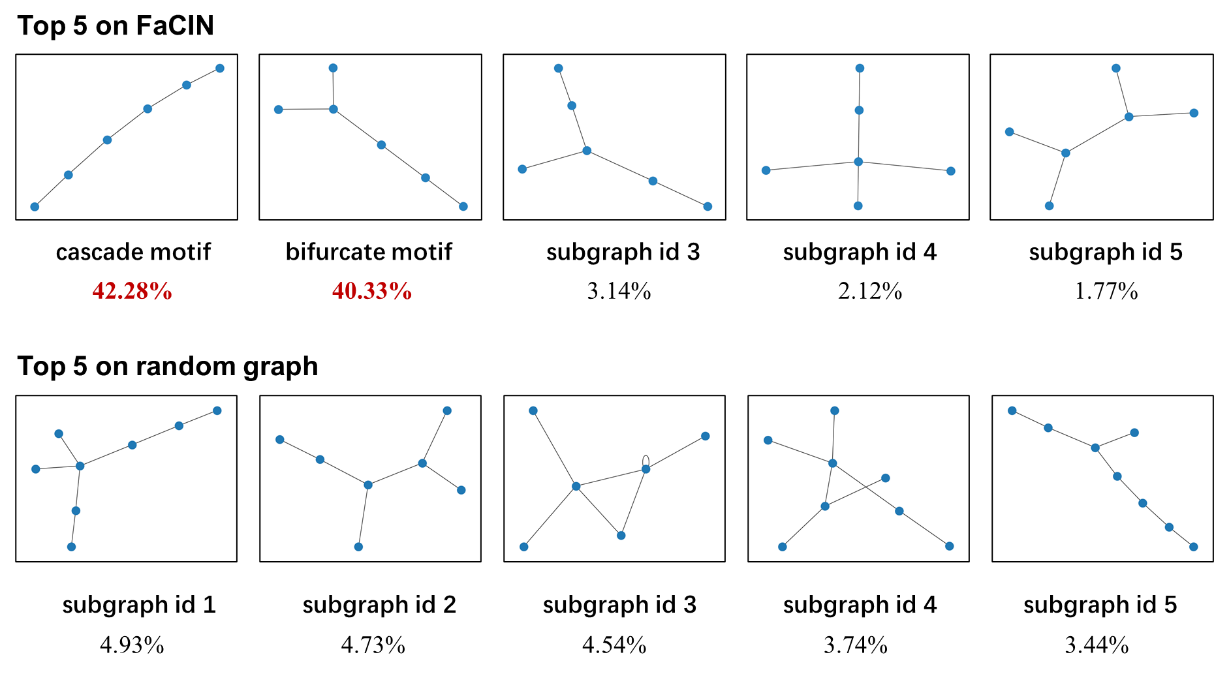


**Fig. 11 for reviewer.** Comparison between motif obtained from FaCIN and

random Hi-C interaction.

**For comment b:** We are sorry that the previous conclusion was not strict. By comparing with motifs from random Hi-C interaction, we can tell that the cascade motif and bifurcate motif generally exhibit a slender shape. They are actually a necessary reflection of the bottleneck-like FaCIN in terms of topological structure, while unable to provide sufficient evidence.

*9. Regarding the chromatin loops:*

***a.*** *It is necessary to state their definitions.*

***b.*** *The number (19,632) seems very small if "chromatin loops" simply mean reliable chromatin interactions.*

***c.*** *The mentioning that "Loops are basically groups of interactions with special functions, such as enhancer-promoter (E-P) linkage" is confusing. Is functional role a requirement for a chromatin interaction to be called a chromatin loop? Shouldn't "chromatin loop" be a more general concept?*

**Response**: We appreciate the reviewer’s careful examination.

**For question a**: Indeed, our previous definition and description of loop were not clear, which may confuse readers. We revised the definition of chromatin loop as “*Loops are pairs of loci that show significantly closer proximity which tend to have special functions such as enhancer-promoter (E-P) linkage*” [*Rao, Suhas S P et al. Cell (2014)*].

**For question b**: We downloaded the loops list from the work of Salameh et al. [*Salameh, Tarik J et al. Nature communications. (2020)*]. They used a supervised learning framework named Peakachu for chromatin loop detection in the same data of ours and achieved great performance. The number (19,632) was in line with expectation.

**For question c:** We agreed with the reviewer that the functional role is not a requirement for a loop. We hope the new definition of loop “*Loops are pairs of loci that show significantly closer proximity which tend to have special functions such as enhancer-promoter (E-P) linkage*” can dispel the confusion.

*10. In the whole paper, when p-values are reported, the corresponding statistical tests are not specified.*

**Response**: Thanks for the reminding and the corresponding descriptions have been rewritten as follows (in red):

1. “We found that the neck interactions were significantly enriched in loop anchors (p < 0.001, hypergeometric test, Fig. 3a).”. Specifically, given the size N of the whole-genome interactions and the number of loops M (the desired attribute is owing loop anchors), the hypergeometric distribution is used to measure the probability of drawing *k* interactions owing loop anchors over *n* trials (without replacement), where *n* is equal to the number of neck interactions.

2. “In addition, we identified 1,242 loops with DSBs on one anchor and another anchor linked by neck interactions (Fig. 4b, Additional file 3), and this number was significantly higher than that for random interactions (p < 0.001, hypergeometric test).”. Likewise, the enrichment is also measured by hypergeometric distribution except that the desired attribute changes to that at least one loop anchor has DSB.

3. “We found that neck neighbours of DSB sites were significantly enriched in coding regions, including CDS and UTRs, compared with those of non-DSB sites (Fig. 4c, p < 0.001, chi-square test).”. We employed chi-square ($\chi^{2}$) test to determine whether there is a statistically difference between the observed frequencies of neck neighbours showing up in coding regions, for DSB and non-DSB two categories of center node.

**Specific Comments:**

*1. Contacts in a Hi-C map may be due to 3D chromatin interactions or genomic rearrangements. The authors have not made any efforts to distinguish between them.*

**Response**:

The reviewer is correct that contacts in a Hi-C map may be due to genomic rearrangements. In our previous manuscript, **we reported the results from NHEK cell line** and it is **a normal cell line**. **In** **HiNT** [*Wang, Su et al. Genome biology (2020)*], a recently developed computational **method for** **detecting copy number variations and translocations from Hi-C data**, the **NHEK cell line**, together with other four normal cell lines, are **used to** **construct a clean background** interaction matrix **to eliminate the biases of cancer cell** lines by background subtraction. Thus, we reasoned that **the impact from genomic rearrangements is relatively limited.** Therefore, it seems not very necessary to deal with the genomic rearrangements at current stage.

But this issue, to distinguish real chromatin interactions from the genomic rearrangements, is certainly worth a re-design for Hi-C preprocessing should our model be applied to tumour datasets. We thank the reviewer for pointing out the direction to improve this work.

*2. Transcription is described by the authors as "a threat to genome stability", but in fact* *transcription-coupled repair is also a major mechanism for maintaining genome stability.*

**Response**:

Thanks for this comment. Exactly as the reviewer said, **transcription-coupled repair is indeed a major mechanism to maintain genome stability**. **It is on the grounds that unrepaired DNA lesions interfere with transcription** and describes how transcription-associated events promote the repair **for an existed DSB**. For example, transcription-coupled nucleotide excision repair (**TC-NER**) is triggered by the installing of elongating RNAPII molecules at DNA lesions [*Xu, Jun et al. Nature (2017)*].

However, when we talk about **the factors contributing to DSB**, **the transcription also is a major part**, as **the dynamic DNA structure prepared for transcribing a gene actually impairs the genome stability** [*Ui, Ayako et al. Cancer science (2020)*; *Uusküla-Reimand, Liis et al. Genome biology(2016)*]. For example, DNA breaks are often induced to relieve the torsional stress from replication or transcription [*Puc, Janusz et al. Nature reviews. Molecular cell biology vol. 18,8 (2017)*]. We therefore speculated that it was our rough using of ‘transcription’, a general word, that should be responsible for the confusion. To avoid such a misunderstanding, we have specified the biological context wherever the role of “transcription” is mentioned. For example, “Besides, topological stress mediated by transcription or replication as well as periodically spaced DNA bending also plays a positive role in DSB formation.”

If the above fails to clear up the question, we will be glad to offer further improvements.

*3. "E edges represent Hi-C contact maps between nodes" -> "Edges in E represent Hi-C contacts between nodes"*

**Response**: Thanks for the careful corrections. We have changed the previous sentence into “…, where nodes in V represent 5-kb genome bins and edges in E represent Hi-C contacts between nodes”.

*4. "Its bottleneck pattern indicates that the aggregated information ultimately flows to one destination" -- Although the Hi-C interaction network is undirected, the GAT layers are ordered and therefore there is a direction of information flow. Does* *the "bottleneck pattern" really support "flows to one destination"?*

**Response**: To better answer the reviewer’s question, **we first translate it into a straightforward manner**, that is, does the “bottleneck pattern”, which derives from undirected Hi-C interaction network, really support “flows to one destination”, which exists in the ordered GAT layers? In case we miss any concern, we next **provide explanations** to the two possibilities in what follows.

**If the above translation is correct, the answer is no.** **The bottleneck pattern is not related to the computation process in GAT layers**. Before further explanation, it seems necessary for us to first clarify the concept of “***aggregated information***”. **Here the information is biological but not computational**. A brief comparison between them is given in following table (only involve their properties mentioned in our work, not for strict definition):

**Table 6 for reviewer.** Comparison of biologically and computationally aggregated information in this study

|  | Biological information | Computational information |
| --- | --- | --- |
| Data | Via chromatin interactions, the signals generated from events thousands of bases apart, such as genes transcription, TF binding and DNA or histone modification, they can often be brought directly or indirectly into proximity to any given site or particularly those DSB sites. | All depends on the input data fed into the GNN network.  Here, for each input graph, its topological structure is from Hi-C data, its node feature is from DNase-seq, ChIP-seq data and K-mer DNA sequence. |
| Information aggregation | Implicit.  Consists of heterogeneous biological signals and can be approximately modeled by aggregating different signals like chromatin accessibility and activity of genes being transcribed and so on. | Explicit  Composed of differently structured data like signal matrix in Euclidean space and graph data and so on. Mathematically subject to the whole GNN network. |
| Outcome | Integrated influence on DNA fragility of specific sites. | Predicted possibility for DSB event occurs at specific sites. |

**If the translation is not correct**, then the reviewer’s question is exactly about **why the bottleneck pattern supports the direction of the biologically aggregated information flow**. Given the abstract properties of biological information, **we discuss it based on two general characteristics of biological principles**.

**First**, genes, chromatin structures and chromatin modifications, they all convey information. This **information is further organized in a hierarchical structure** whose features are ordered, constrained and related [*Searls, David B. Nature (2002)*; *Zhao, Shuai et al. Nature reviews. Cancer (2021)*].

**Second**, the **well-known concept of “feedback”** plays a fundamental role in biological systems. Despite the huge differences compared with cybernetics, one of the most distinctive characters of life is attributed to its informatics properties as a complex system that can handle information and respond to kinds of stimuli [*Levchenko, Andre et al. Current opinion in biotechnology (2014)* and *Suderman, Ryan et al. Proceedings of the National Academy of Sciences of the United States of America (2017)*]. For example, the interactions between ligand-receptor pairs in cell-cell communications are typically analyzed as **information flows** **where the “direction” does exist** **during** **signal transmitting** [*Jin, Suoqin et al. Nature communications (2021)*].

In current DSB-related issue, when we focus on any genomic region, it can be either an undertaker that receives all signals leading to DSB [*Canela, Andres et al. Cell (2017)*], or an initiator that triggers a series of responses to recruit repairing proteins [*Miné-Hattab, Judith et al. Trends in cell biology (2013)*]. For simplicity, we here do not consider DSB repair. **Taking DSB as a result of the gathered information from genomic regions at distance** (which form the interactions in FaCIN), one can easily tell **the direction is towards the break site**. **Just like some tributaries of a river will converge into a main stream before the river reaches the sea**.

**Given the above two points, plus the fact that only 1.6 direct interactions connect with the prediction site, the bottleneck pattern supports the overall consistent direction in this biological “information flow”.** To avoid the above misunderstanding, we have changed the description of corresponding part.

*5. "Motifs, such as* *transcription factor-binding site and* *regulatory network motifs" -- These are very different types of motifs that are seldom considered together.*

**Response**: Thanks. It is an awkward mistake. We here intended to use these two different concepts to exemplify the abstract motif of both biological significance and topological pattern. But we neglected that these two motifs, transcription factor-binding site and regulatory network motifs, should not be compared with either logic or evidence. **Since we focus on motif searching on graph, we remove the TF-related words**.

*6. "The most frequent motifs exhibited a 'forward chain' and 'binary-parallel' mode":*

***a.*** *Why is it considered "forward"?*

***b.*** *Why is it called "binary-parallel" here and "bifurcate" in Figure 2c?*

**Response**:

**For question a**: We regret that the "forward" motif was named with puny consideration. For a unified purpose, we at first described it as a “forward” chain because we wanted to include all the structural settings no matter the specific directions between each cascaded node pairs are coherent or incoherent. But the reviewer’s question makes us realize that the implicit direction of the word “forward” should not be neglected. We have replaced the “forward” with a more appropriate term “cascade”.

**For question b**: The “binary-parallel” was initially misused and afterwards, the one in figure 2c was corrected to “bifurcate” while the one in text was left uncorrected due to carelessness. We have ensured their names are consistent in the revised manuscript.

*7. "recent Hi-C experiments have generated more than 30 publicly available datasets" -- This sentence suggests that the authors do not really have a good idea of the amount of publicly available Hi-C data.*

**Response**: Thank for the kind reminding. We reinvestigated and in detail summarized the publicly available Hi-C data collected from cell lines, normal tissues, and tumor tissues. In the revised version, we have added over 200 publicly available Hi-C datasets in the file attached as *file_3_for_referees-Summarized_Hi-C_available_datasets.xlsx*.

**-------------------------------------**

**Response to Reviewer #3**

**-------------------------------------**

**Reviewer #3 (Remarks to the Author):**

**General comment:**

*The authors proposed a deep graph neural network (GNN) model to predict DSB from DNA sequence and 3D chromatin structure. They then applied GNNExplainer, a masking-based method to study feature importance, and defined "DNA fragility-associated chromatin interaction network" (FaCIN), a connected graph of top important features and edges predicted by GNNExplainer. They found that that direct interactions in FaCIN (which they called neck interactions) showed interesting associated with TAD and loops, and argued that they can serve as chromatin structural determinants of DSB formation. While the application of GNN and GNNExplainer to study the impact of 3D chromatin structure on DSB is novel, more details should be provided on the methods description, and additional experiments should be performed to establish that the FaCIN learnt by the model are biologically meaningful. See below for detailed comments.*

**Response**: We thank the reviewer for the positive evaluation for the manuscript and so on. According to following comments, we have added necessary experiments as well as the analysis required by the reviewer. Our detailed responses to the comments are as follows (changes in revised manuscript are marked in red).

**Major comments:**

1. *Method descriptions:*

*While the algorithm details of GNN and GNNExplainer is well described, detailed description of the preprocessing of Hi-C is missing. Preprocessing of Hi-C data is crucial to remove biases and to identify real contact events. This step is thus crucial for the model to learn meaningful connections associated with DSB. However, the authors provided zero description of the preprocessing of the Hi-C data.* *Did the authors use raw or preprocess contact map? How is it normalized? At what cutoff is a contact considered an edge in the graph? This information is important for a fair evaluation of the model performance, and whether the features the model learnt are biologically relevant, or just systematic biases in Hi-C experiments.*

*In addition, the following information are also missing from the Methods section:*

***a.*** *details of all statistical tests being performed.*

***b.*** *in the section of characterization of neck interactions, authors mentioned "we detected E-P loops", how are E-P loops detected. Are enhancers defined based on using histone marks?*

***c.*** *how is "topological subgraph search" performed?*

**Response**: Sorry, that in our previous manuscript some details of Methods are missing. For Hi-C data preprocessing, the details are as follows:

1. We used raw contact map.
2. We did not perform normalization. Because FaCIN focuses on revealing a universal form in terms of chromatin interaction and thus what required by FaCIN is exactly the raw data as global as possible.
3. We did not perform a strict filtering and only discarded the contacts of value ≤ 1. The reasons are two-fold. One is mentioned in above point 2. Another is because in complex networks, patterns that are functionally important but not statistically significant could exist [*Milo, R et al. Science (2002)*], which otherwise would be missed if we make much filtering. Therefore, almost every non-zero contact was taken as an edge. We entirely agree with the reviewer that the details of Hi-C preprocessing are rather crucial. Thus, we have added this part in the Methods section in the revised manuscript.

**For comment a**: Thanks for the reminding and the corresponding descriptions have been rewritten as follows (in red):

1. “We found that the neck interactions were significantly enriched in loop anchors (p < 0.001, hypergeometric test, Fig. 3a).”. Specifically, given the size N of the whole-genome interactions and the number of loops M (the desired attribute is owing loop anchors), the hypergeometric distribution is used to measure the probability of drawing *k* interactions owing loop anchors over *n* trials (without replacement), where *n* is equal to the number of neck interactions.

2. “In addition, we identified 1,242 loops with DSBs on one anchor and another anchor linked by neck interactions (Fig. 4b, Additional file 3), and this number was significantly higher than that for random interactions (p < 0.001, hypergeometric test).”. Likewise, the enrichment is also measured by hypergeometric distribution except that the desired attribute changes to that at least one loop anchor has DSB.

3. “We found that neck neighbours of DSB sites were significantly enriched in coding regions, including CDS and UTRs, compared with those of non-DSB sites (Fig. 4c, p < 2.2e-16, chi-square test).”. We employed chi-square ($\chi^{2}$) test to determine whether there is a statistically difference between the observed frequencies of neck neighbours showing up in coding regions, for DSB and non-DSB two kinds of center node.

**For question b**: We downloaded the enhancers from a highly recognized database EnhancerAtlas 2.0 [*Gao, Tianshun, and Jiang Qian. Nucleic acids research (2020)*]. In this database, Gao et al. defined enhancers based on 12 high-throughput experiment methods (e.g. H3K4me1/H3K27ac, DNase-seq/ATAC-seq, P300, POLR2A, CAGE, ChIA-PET, GRO-seq, STARR-seq and MPRA). They developed an unsupervised learning approach to weigh each track of experiment methods and combined them to determine the consensus enhancers. The promoters are defined as the intervals (−2000bp to +2000bp) around a transcriptional start site (TSS). If a 5-kb genome bin overlaps with a promoter (an enhancer), then we refer to it as a promoter node (enhancer node). Then, an interaction that connects one enhancer and one promoter was used as E-P interaction.

**For question c**: The search for topological subgraph aims to find whether FaCIN contains enriched motif. For all the FaCINs obtained from an individual chromosome, the searching is performed as follows:

1. Create an empty list in which to store the subgraph and its occurrence number.
2. Examine a FaCIN and use a *is_isomorphic* function (from NetworkX, a Python package) to determine whether current FaCIN is identical to any existed subgraph in list. If it is, the number of correspond subgraph plus 1. If it is not, store the current FaCIN as a new subgraph in the list. Continue this step until examining through all FaCINs.
3. At last, count occurrence numbers for all present subgraphs and only those subgraphs with significantly higher occurrence number can be taken as motifs.

As a result, our reported motifs are organized in a chromosome-wise manner and, the cascade motif and bifurcate motif are the top-2 out of all candidates which account for over 80% FaCINs on whole genome. Please find above results in file *file_4_for_referees-Top_5_motifs_of_FaCINs.docx*.

1. *Benchmarking:*

*The main contribution of this manuscript is building a DSB prediction model by incorporating 3D chromosomal information. And the focus is on how 3D interactions contribute to DSB. Thus, it is important to establish that by incorporating 3D information, the model can predict DSB with improved performance. An important benchmark would be comparing the GNN model to a model that predicts DSB from the same set of features (CTCF, DNase and k-mers), but without Hi-C information.*

**Response**: Thanks for this suggestion. We agree with the reviewer that it is important to prove the contribution of incorporating Hi-C. **A stringent way is to re-train our model DSB-GNN by dropping Hi-C information while retaining other features**. **However, it is not applicable** because we used Hi-C contact maps to construct the graph as the basic input and without Hi-C information, all the other features (CTCF, DNase and k-mer) will find nowhere to attach to. Therefore, as the reviewer suggested, we turned to traditional machine learning methods that can work without Hi-C information, such as LightGBM [*Ke, Guolin et al. NIPS (2017)*] and Random Forest (RF) [*Breiman, L. Machine Learning (2001)*], which are often used to benchmark deep-learning works.

**The comparisons combined with ablation studies are shown** in the *Table 1 for reviewer* below.

**Table 1 for reviewer.**

| Feature | **DSB-GNN**  AUC | **LightGBM**  AUC | **RF**  AUC |
| --- | --- | --- | --- |
| 3-mer | 0.7620 | 0.7298 | 0.6100 |
| 4-mer | 0.7700 | 0.7321 | 0.6184 |
| 5-mer | 0.7756 | 0.7256 | 0.6153 |
| k-mer | 0.7857 | 0.7331 | 0.6499 |
| DNase | 0.8865 | 0.8668 | 0.6984 |
| CTCF | 0.8531 | 0.8245 | 0.6751 |
| k-mer +DNase +CTCF | **0.9251** | 0.8862 | 0.6598 |

From the results, we can learn the proposed model DSB-GNN consistently performs better over LightGBM and RF across different sets of features. Besides, the integrated features brought a boosted performance for all three methods.

1. *The authors should provide more evidence that the FaCIN reveals a bottleneck pattern.*

*The authors observe that while each prediction site has on average 91 direct interactions, only 1.6 out of top FaCIN interactions are direct.* *91 and 1.6 are not comparable, since author mentioned in the Methods that for FaCIN, "we limit the (FaCIN) graph size to be max 10 top ranking edges …". Thus, the comparison in* *Fig. S3 is misleading. A fair comparison should be comparing (# direct interaction / # total interaction) for each prediction site to 1.6/10 in FaCIN. A* *statistical test should be performed to suggest whether FaCIN is enriched/depleted of direct interactions.*

**Response**: Thanks for pointing out the above deficiencies. **We divide the comment into three questions**:

**a**. more evidence is needed to support FaCIN’s bottleneck pattern;

**b**. the comparison between all direct interactions and neck interactions is unfair;

**c**. statistical test is needed to prove that FaCIN is enriched/depleted of direct interactions.

**For question a**: It is very difficult to strictly prove that a graph has a certain shape, since **shape is unlike any regular properties of graph such as connectivity or density that we can calculate, it is about the visible characteristic** of an item or its outline or outer surface. It depends on how we look at it. **We therefore explain FaCIN’s bottleneck shape by giving both example** and **statistics**.

**Example**: If we overlook the node types, the following graphs (in *Fig. 12 for reviewer*) are all isomorphic as they all contain the same number of nodes connected in the same way.


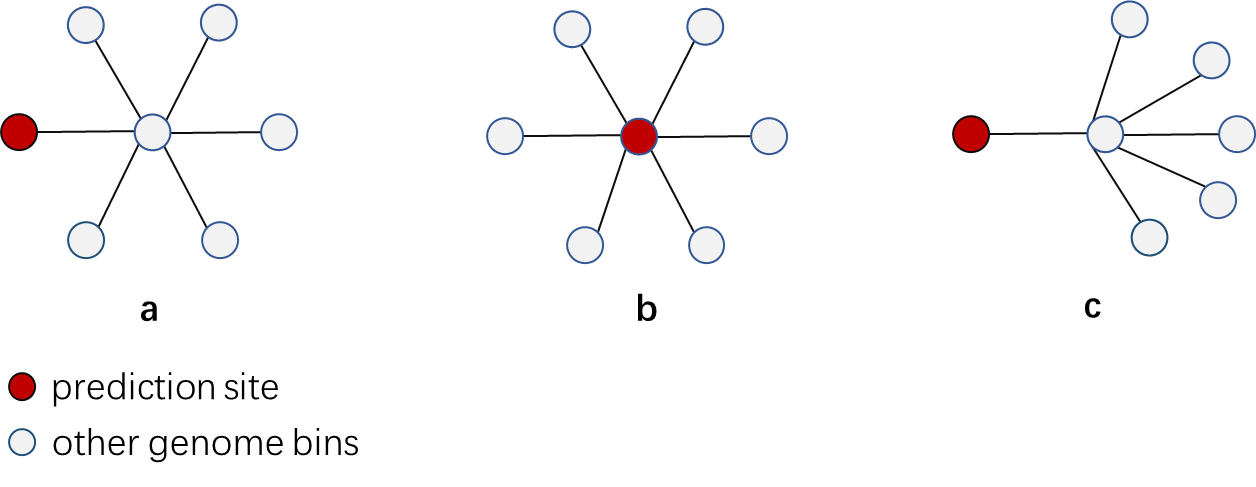


**Fig. 12 for reviewer.** Comparison between bottleneck pattern (a, c) and cycle pattern (b).

**However, a FaCIN cannot be determined unless its prediction site is determined first**. **So,** **the prediction site must precede other genome bins** **and it is the core of** how the fragility of a piece of DNA is affected by associating with the whole genome. **Therefore, the nodes should not be treated without distinction**. In above figure, graph **a** is what we referred to as a typical **bottleneck** **pattern**, and its shape becomes more visible when we adjust it into a totally equivalent graph **c.** This pattern illustrates a manner where the prediction site directly communicates with one neck neighbor and the neck neighbor gathers or pools the biological information from many more genome regions at distance. While graph **b** is another kind often called as a **cycle** **pattern** and it describes an entirely different manner where the prediction site is evenly affected by multiple neighbors around. These two patterns correspond to quite different meanings.

**Statistics:** We calculated the **betweenness centrality** for each node in FaCIN. **Betweenness centrality indicates how often a node appears on the shortest path** **between any random node pairs and it actually measures how well a node is connected across the whole graph**. For example, in the graph below (*Fig. 13 for reviewer*), there are three 3-step shortest paths between the red and green nodes, and two of them have the yellow node, so the betweenness probability of the yellow node for the red-green node pair is 2 / 3 = 0.667.


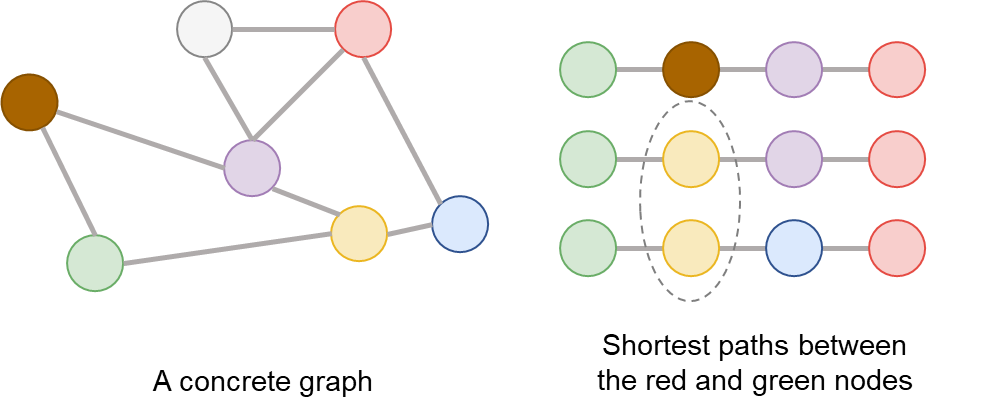


**Fig. 13 for reviewer.** An example to illustrate how to calculate betweenness centrality.

We calculated the **betweenness centrality of each node for the node pair of prediction site and any other node**, and we classified them into two groups according to whether the node belongs to 1-hop or 2-hop neighbors. For this metric, the higher the value, the stronger the mediating or broker role. Thus, in a graph of bottleneck pattern, **we can expect that the neck neighbors (1-hop neighbors) should have on average a much higher betweenness centrality** due to their special position, and we did observe the result as expected in *Fig. 14 for reviewer*.


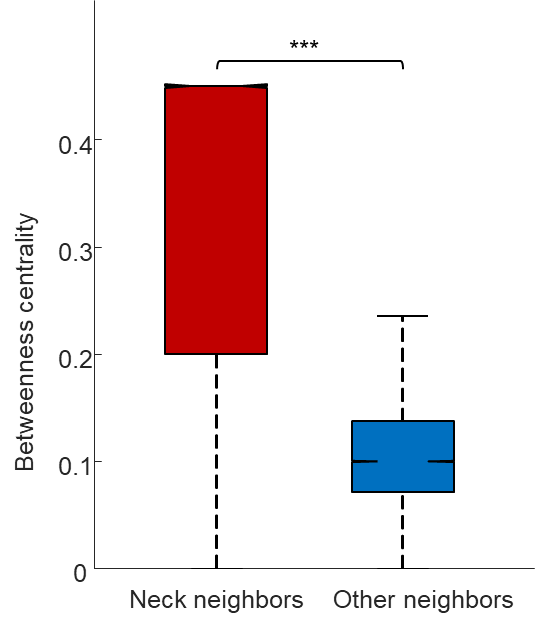


**Fig. 14 for reviewer.** Comparison for betweenness centrality of

neck neighbors and other neighbors. (*p < 0.001, t-test*)

**For question b**: We agree with the reviewer that in this comparison FaCIN’s upper limit of 10 interactions should not be omitted and otherwise we might be misunderstood, thus we changed the Fig. S3 adding a mediating bar to indicate this upper limit.

**Our intention** for comparing 91 and 1.6 was **to emphasize that not all direct interactions were important** because there are **nearly a hundred candidates** of direct interactions, but **only 1.6 of them show up in FaCINs**. This is interesting, as **intuitively we expected the direction interactions might account for the majority of FaCIN**. For example, think of **a corner case**: assume the average direct interactions of a prediction site is less than 10, and nearly every direct interaction is present in FaCIN. **This seems to more accord with our intuition** and means that direct interactions have absolutely dominant influence over prediction sites than those indirect. **But the results did not turn like that**.

Besides, **some indirect interactions went ahead of direct ones might reveal a more complicated DSB mechanism than it appears**.

**For question c**: **As the reviewer suggested, we** **added a hypergeometric test**. Direct interactions and indirect interactions within 2-hop region for each prediction site are 91 and 9446 on average. Therefore, assume there is an interaction set contains 91 direct and 9446 indirect and we randomly choose 10 of them. The probability that we choose 1.6 direct interactions is around 0.0915 (using *phyper (lower.tail=F)* function in R). **This result does not support a statistically significant enrichment or depletion**. **The point we exactly intended to highlight**, **was** **not the distribution of direct interactions**, **but the much more complicated driving factors of DSB**. As previous work reported, DNA breaks are often induced to relieve torsional stress in response to replication or transcription [*Uusküla-Reimand, Liis et al. Genome biology. 2016*]. **Likewise, FaCIN’s interactions might play a role in transmitting biological information (like physical stress) to the prediction site, in a cascading manner**, which account for the relatively small number of direct interactions in FaCIN.

1. *Questions regarding the features used for GNN model.*

***a.*** *Why use kmer as features? Why not use convolutional filters as feature extractors as used in many deep genomic models? 3,4,5-mers used in this study can lose information on local positional dependency, Which longer k-mers or CNN kernels can better captures.*

***b.*** *Peak number is not a good estimator of local accessibility, it is sensitive to the smoothing parameter of the peak caller. A sequence of small peaks can be called as a "broad peak", and similarly sometimes broad peaks can be split to smaller peaks. Especially for DNase, having a very broad peak versus a single narrow peak have very different meaning for the accessibility of the bin, while both will have a peak number of one. The authors should provide more justification for their choice of features.*

**Response**: Thanks for this comment and we split it into two questions.

**For question a**: About **why we used k-mer features** and **why not choose CNN** as feature extractors or using longer k-mer.

**We choose k-mer features** **due to the amounting evidence for the genome-wide DNA sequence preference of DSB events** (please find in the table below)**. However, the sequences reported in these works are all scattered.** We wonder whether a simple combination by bases of short length would give any hint that a DSB is prone to occur. Admittedly, **using convolutional neural network (CNN)** can integrate more local positional information and thus **will very likely promote the prediction performance.** **But** **in this way, the feature for each genome bin is generated from the hidden embeddings of several convolutions, which is much less interpretable** compared to k-mer features. Using **longer k-mer** will lead to more costly computation as one base increase for k-mer will bring more than 4 times computational cost. For a trade-off between performance and interpretability, we used k-mer (k=3, 4, 5) DNA sequence instead of CNN.

| **Table 7 for reviewer.** | | |  |
| --- | --- | --- | --- |
| **Year** | **Title** | **Journal** | |
| 2013 | Hyperthermia-induced genotoxic effects in human A549 cells | Mutation Research | |
| 2019 | The sequence preference of gamma radiation induced DNA damage as determined by a polymerase stop assay | International Journal of Radiation Biology | |
| 2019 | Comparison of Different Methods to Determine the DNA Sequence Preference of Ionising Radiation-Induced DNA Damage | Genes | |
| 2019 | The genome‑wide sequence preference of ionising radiation‑induced cleavage in human DNA | Molecular Biology Reports | |
| 2019 | Activation of Oncogenic Super-Enhancers Is Coupled with DNA Repair by RAD51. | Cell Reports | |

**For question b**: We are sorry about the unclear description and we actually calculated the DNase I signal density for each genome bin of 5-kb. We have corrected this part in Methods section.

1. *The paragraph on "motif search" is confusing.*
2. *The authors discussed "motif analysis" in terms of "motifs, such as transcription factor binding site and regulatory network motifs", but the analysis is on topological subgraphs. A TF motif search would involve performing TF motif enrichment analysis using tools like FIMO or Homer to look for enriched PWM at DNA sequences.*
3. *The author looked for enriched topological subgraph, which is not related to TFs. The authors also didn't explain their approach in the Methods,*
4. *The authors didn't explain the biological relevance of the top hits presented in fig. 2c or additional file 2.*
5. *Why does* *the presence of "forward chain" and "binary-parallel" suggest that "**FaCIN contains the universal building blocks for chromatin organization at the interaction level"?*

**Response**: We split this comment into **four questions** and then give our response to them point by point.

**For comment a: About the vague beginning of this paragraph** (the last paragraph in Result2). Sorry we left a confusing impression. After a reexamination, we speculate that the reason behind this confusion is our **farfetched combination** of two different concepts—TF motif and network motif. We regret this awkward mistake. We originally intended to use the two concepts to exemplify the word of motif under the context of biology. **We meant to specify the motifs of our interest are those with both topological pattern and biological significance**. But we neglected that they two, transcription factor-binding site and regulatory network motifs, should not be compared with either logic or evidence.

**Since we focus on motif searching on graph, we remove the TF-related words**.

**For comment b:** The motif searching **aims to find the topological subgraphs that recur with significantly higher times than expected by chance**. In our revised manuscript, we have **added the details (in Methods section)** to illustrate how we performed this searching.

**For comment c: About the biological relevance of the top two motifs presented** in Fig. 2c or additional file 2.

**First, we performed an additional experiment to prove the top two motifs were enriched in FaCINs**. Specifically, we generated **randomized graphs using Random Walk on raw Hi-C contact maps and they have the same overall characteristics as does the FaCIN**: a connected subgraph for a genome bin which covers at maximum a two-hop region with no more than 10 edges. Then, we **performed subgraph searching** on these randomized graphs and one can tell the **difference** between the FaCIN-enriched motifs and new obtained, **from both visual shape and frequency**. (*Fig.11 for reviewer*). **The** **new obtained account for patterns that appear only because the general Hi-C interactions**. We also attached above results on randomized graph in a compressed file named *file_2_for_referees-Subgraph_search_result_on_random_graph.zip*. Due to file size limitation, it only includes results from chr1-chr3.


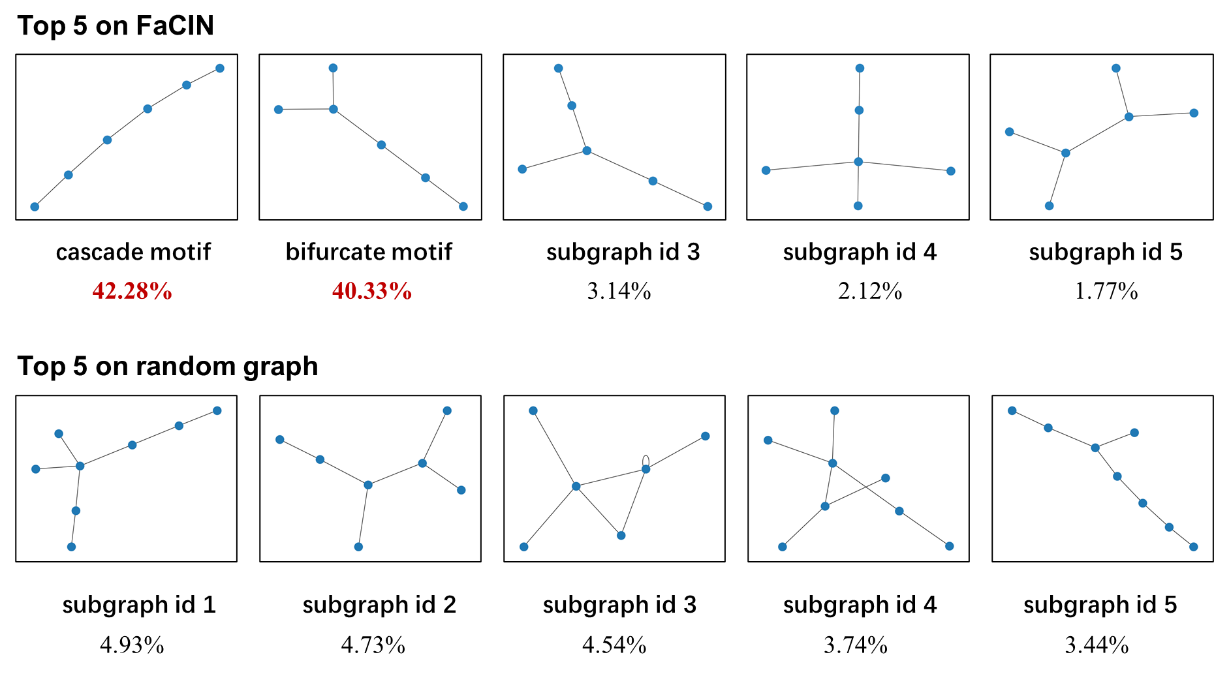


**Fig.11 for reviewer.** Comparison between motif obtained from FaCIN and

random Hi-C interaction.

**Second**, GNNExplainer [*Ying, Rex et al. Advances in neural information processing systems. (2019)*], as a GNN interpreting technique, has been applied into many tasks across different fields, such as predicting the mutagenic effect of a given chemical molecule graph and discerning the Question-Answer (QA) interactions from Online-Discussion interactions. The following *Fig.5 for reviewer* is taken from the original work of GNNExplainer, which briefly **summarizes the learnt crucial structure of GNNExplainer to make decision**. With the strong power of GNNExplainer, one can tell that **the learnt crucial structure quite matches the nature of corresponding tasks**. For example, the ring structure in mutagenic effect prediction task and the topic reactions in QA task, they both well match the prior knowledge.


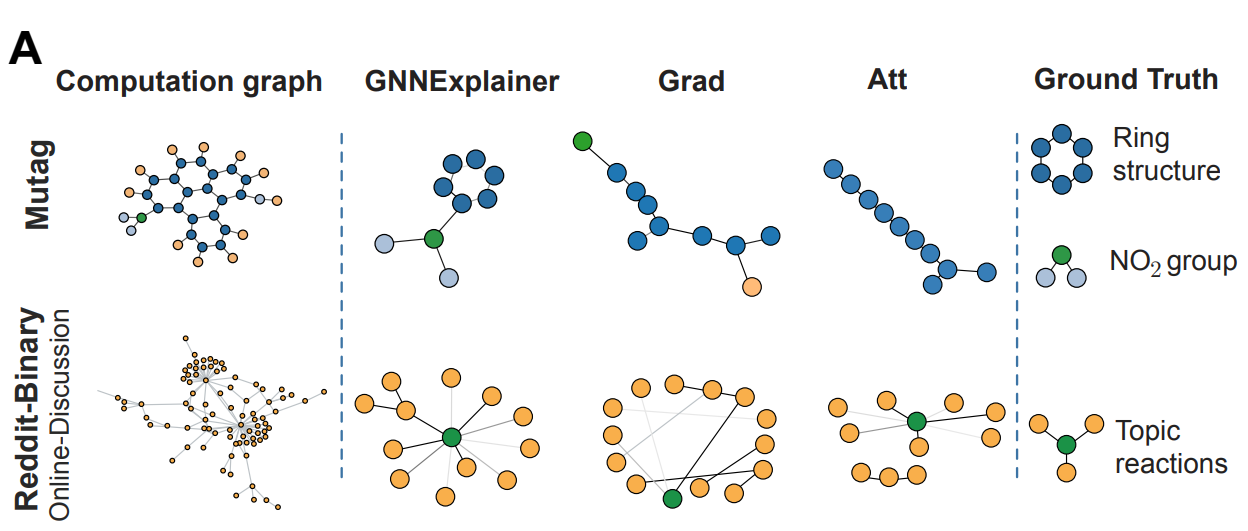

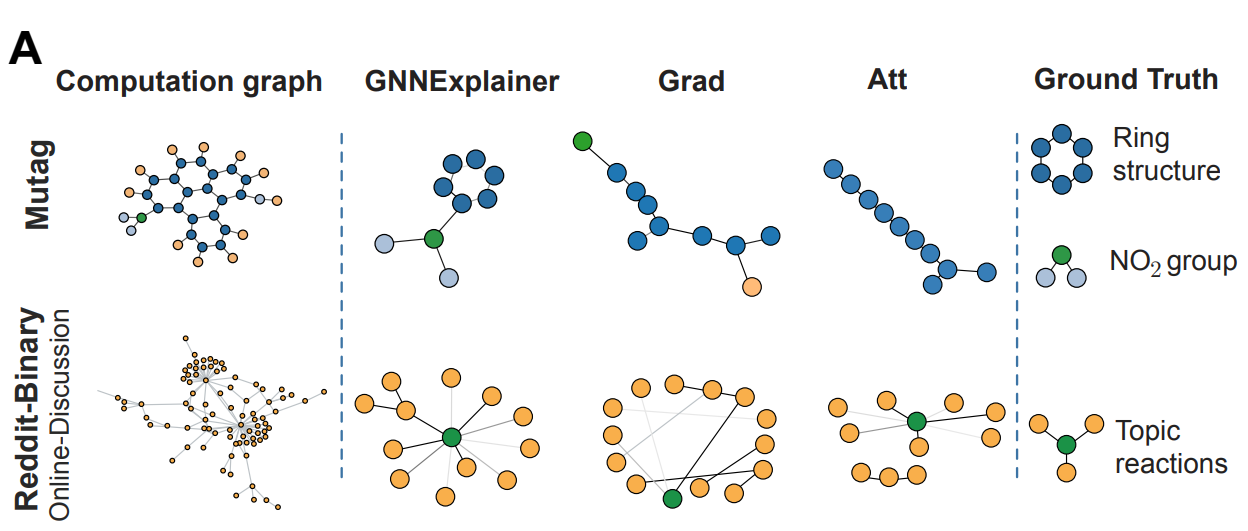


**Fig. 5 for reviewer**. An example to illustrate how GNNExplainer makes decisions.

Top: mutagenic effect prediction. Bottom: forum discussion classification.

**Above findings from other similarly complex domains can also illuminate and inform the importance of the enriched motifs in FaCIN.** Likewise, by implementing GNNExplainer in this DSB-related task, we identified the bottleneck-like FaCIN, and we believe this pattern as well as the its neck interactions are of biological importance for DNA fragility.

**For question d:** About why the presence of two top motifs suggest that "FaCIN contains the universal building blocks for chromatin organization at the interaction level".

*(Although we have carefully gone through the context of this sentence, we are still not sure whether the following interpretation can approach the cause of this question. We politely ask for more clues if we fail to get the right question.)*

The reviewer **seems to question the validity of our summary about the potential role of FaCIN**, or rather, **ask for the causal evidence linking** the presence of top hits motifs and the corresponding claim that FaCINs contain universal building blocks. This is a little beyond what we were meant to convey. As there is **no causal relationship between the above two things**. **Instead, they are basically talking about the same thing and their relationship is more like the inside and the outside**.

**We speculate that it is our previous presentation** that “*These motifs … suggest that FaCIN contains the universal building blocks …”* **resulted this confusion**. *Suggest*, an overused verb, here brought a sense of causal implication. To clarify this, we have rewritten this as “… the existence of motif was in itself informative, indicating that FaCINs contain the universal building blocks ....”.

**Specific Comments:**

***1.*** *GNNExplainer assigns importance scores to different input features. The authors only focused on CTCF and DNase features. What about the other k-mer features. Any explanation for the top k-mer features? Do they match any known sequence patterns such as TF motifs?*

**Response**: We appreciate the reviewer’s keen insight into this point.

**The Top 10 important k-mer features are shown *in the Fig. 15 for reviewer* (the bule boxed)** **and they all belong to 5-mer group.**


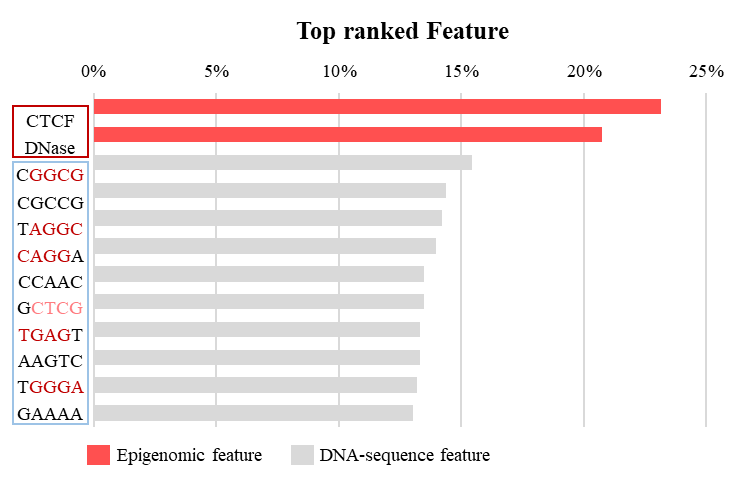


**Fig. 15 for reviewer.** Features that ranked top for DSB prediction.

**We** **used MEME 5.4**, a motif-based sequence analysis tool, **to extract a 20bp-long sequence that is most recurring near DSB sites** in NHEK cell line. **This DSB-preferred sequence is shown in the *Fig. 16 for reviewer*.**


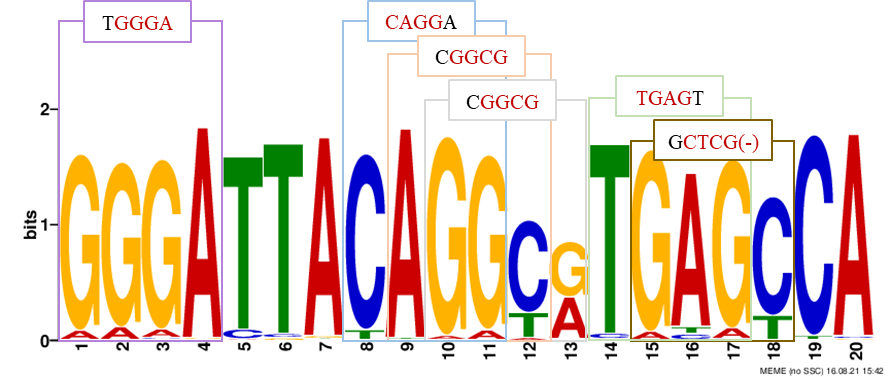


**Fig. 16 for reviewer.** DSB-preferred sequence.

**We found that** **6 out of the Top10 features have a 4-bp consecutive overlap (colored in red in *Fig. 15 for reviewer*) with the DSB-preferred sequence.**

*Probability for any 5-mer feature matching 4-bp of the DSB-preferred sequence by chance is:*

*((20−4+1)×4×2)/4^5 =136/1024≈0.13*

*where 20-4+1 is the maximum number of possible locations, 4 represents the four possibilities (T,C,G and A) for the one base other than the overlapping part, and 2 represents this one base either appear at the head or at the end. For simplicity, this formula does not consider the impact that might be induced by repeated sub-sequences.*

Therefore, if by chance, the probability we find this matching happen on six 5-mer features will reach at least a magnitude several orders lower than 0.13. Yet these 5-mer features have partial overlap and are not independent, the definite value of probability will for sure fall into 0.13^2 ~ 0.13^6, i.e., 0.0169~4.83E-6. **It suggested that the important features ranked by GNNExplainer do have a link with DSB events**.

However, though we find it interesting, an obvious question confused us: since these 5-mer features have a 4-bp overlap with the DSB-preferred sequence, why do the corresponding 4-mer features (identical with the overlapping part) fail to rank the top? We speculate there exists **a more complex relationship lying behind the simple matching**. One way to explore it might be using longer feature such as 6-mer or 10 mer, but the price is to undertake a costly computation for features amount to 4^6 = 4,096 ~ 4^10 =1,048,576. At current stage, we did not think of a better solution and hence left this issue to receive future answer. In our revised manuscript, we have added above results in additional files.

1. *Fig3a, Fig.3d, and Fig.S4 need better legends. Details of statistical test should be better explained. For example, in Fig. 3a what are the boxes and points? What is the statistical test being used?*

**Response**:

We have rewritten the legends as the reviewer suggested. Statistical information and meanings of different symbols are provided.

1. *"**CTCF and DNase signals were significantly higher at neck neighbors …", what are their importance score with GNNExplainer?*

**Response**: The importance score of CTCF and DNase signals were also calculated with GNNExplainer. The masking strategy also works for node feature. That is, removing the corresponding feature (either CTCF or DNase I) while keep the other information unchanged, the effect of CTCF or DNase I signal can be measured for each genome bin’s prediction. In summary, these two kinds of signals, each has an importance score list for whole genome bins. On average, the score for CTCF signal is 0.585 and for DNase I is 0.578. The distribution of importance score for these two signals with other K-mer features is show in *Fig. 17 for reviewer* below (Fig. S1b in manuscript). This distribution is calculated with importance scores averaged over half a million prediction sites from whole genome. Therefore, even a small margin in the score also makes sense.

**
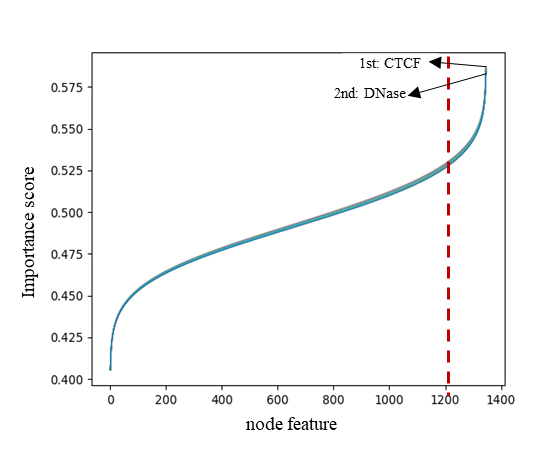
Fig. 17 for reviewer.** Distribution of importance score for 1346 features.

1. *There's only 1.6 neck interaction per bin, but the focus of the analysis is only on neck interactions? Can the authors also discuss the rest of the FaCIN interactions?*

**Response**: Thanks for the suggestion. We previously focused on neck interactions because they play a key role in shaping FaCIN’s unusual bottle-like pattern. Being the slender part of FaCIN, neck interactions carry the most direct exchange of biological information under the 3D genome structure.

As the reviewer suggested, we have also analyzed the other (2-hop) interactions. We found that each prediction site has **on average 9,446 2-hop interactions** and the genomic area lying between prediction site and 2-hop neighbours are considerably enlarged compared to that between 1-hop neighbours. Their median lengths are on average **160-kb and 410-kb**. From the *Fig. 9 for reviewer*, we can learn that FaCIN includes both distal and proximal interactions and it is composed largely of those distal with rich 3D information. The considerable range covered by 2-hop interaction indicates that long range interactions play an important role in affecting DNA fragility.


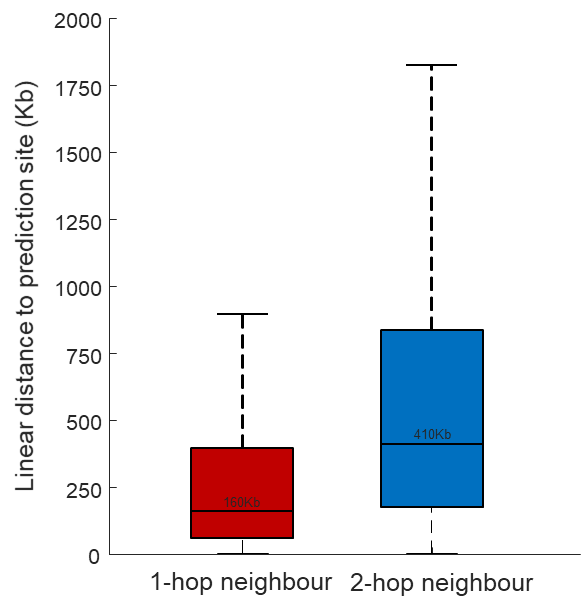


**Fig. 9 for reviewer.** FaCIN’s size in terms of distance from prediction site to 1-hop and 2-hop neighbours.

1. *Citation 18: formatting error.*

**Response**: Sorry for this mistake and we now have fixed it.

**Second round of review**

**Reviewer 1**

This revision has addressed all my proposed issues.

**Reviewer 2**

The authors have spent a lot of efforts on responding to the reviewer comments, which should be commended. Unfortunately, some comments remain not addressed, and some responses are irrelevant to the comments. As a result, the manuscript still contains a lot of fundamental issues.

Comments given in the first round of review that remain not properly addressed are listed below.

1a. The authors have compared their DSB-GNN method with two simple methods, LightGBM and RF, which is useful. However, their claim that they cannot compare with the more specialized methods proposed by Mourad et al. and Ballinger et al. seems not valid for a number of reasons. First, ChIP-seq data (required by these methods) are in general easier to obtain than Hi-C data (required by DSB-GNN). Second, some ChIP-seq data are in fact available for the NHEK cell line. Third, it may be possible to run those two methods with some data types omitted or set to constant/random values.

Also, the authors have not performed the ablation studies requested in the original comment. Therefore, even if DSB-GNN really has superior performance as compared to other methods, the relative contributions of the different components of the method (features, Hi-C network, self-attention mechanism, etc.) to the performance gain remain unclear.

1b,c. The authors emphasized that their main finding is the bottleneck pattern, which is "a universal form of how the fragility of a piece of DNA might be affected (or affect) by the whole genome in three dimensional space. In brief, FaCIN helps to truly dissect the mechanisms driving DNA fragility under the 3D genome". Exactly how bottleneck is defined remains unclear after this revision (see 2a below). What this pattern implies regarding chromatin organization is also unclear. Does it simply mean that if a region is fragile to DSB, other regions close to it in the 3D space are also likely fragile? Or are there additional conditions required such as the detailed connections and chromatin states of those other regions?

2a. In the mathematical definition given, the meanings of |S| and |N(S)| are not stated. Assume |S| means the number of nodes in set S, and N(S) means the set of neighbors of the nodes in S excluding the nodes in S. Then there are many types of subgraphs that match this definition. For example, any connected component is a bottleneck, because the nodes in a connected component are not connecting to any other nodes and thus |N(S)|=0. In the most extreme case, if all genomic regions are connected through chromatin interactions, the whole genome together is a bottleneck. It is therefore unclear conceptually what insights about effects of genome architecture on DSB fragility the bottleneck pattern is supposed to provide.

The authors do not seem to have given the mathematical definition of bottleneck in the revised manuscript, or performed actual calculations to show that the subgraphs they frequently found from the FaCINs are bottlenecks.

The description of Hall's Theorem in the response letter seems completely irrelevant -- the Hi-C contact graph is not bipartite in general, and whether there is a perfect matching between nodes in the Hi-C contact graph is unrelated to the current work.

In the response letter, is is said that "Our bottleneck pattern has a similar extended meaning to that of Hall's theorem, which slightly differs in that the neck interactions have less elements than neighbours." How is the meaning extended? How is Hall's Theorem relevant here?

The fancy figure with two bottles sharing the same neck region is very confusing -- Does the definition of a bottleneck requires two bottles?

2b. A long explanation is provided but it seems relevant to the comment, which is about the rationale for calling direct interactions involving the prediction site "neck interactions".

3. The authors have not responded to the request of comparing the results of different numbers of GNN layers.

4. The authors argue that the robustness of their model "was not affected by different Hi-C normalization methods and this is not surprising because in our model the normalization is no longer a preprocessing step but partly transferred to the GNN model, as the graph weights derived from raw interactions are constantly updated during training." Apparently the authors have mixed up two different concepts, namely Hi-C data normalization, for removing biases, and learning weights in a GNN, for deriving features useful for predicting DSBs.

5b. With a bin size of 5kb, most chromatin interactions would not be considered proximal according to the authors' definition (of <10kb), and thus this definition is not suitable in this context.

The key question, which the authors have not answered, is whether patterns in the Hi-C contact graph related to DSBs are really due to non-trivial 3D genome structures or simply due to 1D genomic closeness. A simplistic binary classification of interactions into "proximal" and "distal" seems inappropriate here.

5c. By using raw counts, there could be noise and biases in the data that affect the analysis.

6. The description of the masking procedure is incomplete. For example, what is the initial masking matrix? What is the loss exactly? What does it mean by "the goal of identifying the optimum subgraph Gs(v) and feature set Xs(v)"? How are the masking coefficients adjusted?

7. The authors claim that they "tried to keep the model's performance as independent as possible from threshold parameters". In fact, requiring a contact count of at least 2 is exactly a demonstration of picking an arbitrary threshold and the performance could be affected by this choice. It is thus not clear why the authors believe they have reduced the dependency of their method's performance on thresholds.

8a. If a FaCIN is a connected subgraph that covers "at maximum a two-hop region", why do some motifs discovered (shown in "Fig. 11 for reviewer") involve nodes that are more than four hops away from each other?

9a. The definition for chromatin loops given is incomplete. How is the significance of proximity determined? Is closeness adjusted for 1D distance or not? It is not clear which subset of Hi-C contacts are considered chromatin loops based on this definition.

9b. Due to the unclear definition of chromatin loops, it is difficult to evaluate whether having 19,632 loops genome-wide is reasonable.

9c. If the functional role is not a requirement for a loop, why is it still part of the definition text within the quotation marks given in the response to 9a?

10. There are some issues about the statistical tests:

1,2. In the hypergeometric test, is the p-value defined as the probability of having exactly k interactions or at least k interactions? The authors' response seems to suggest the former.

The calculations performed by the authors mix the counting of nodes and edges together. Consider a graph A=B-C, where "=" means a loop interaction and "-" means a non-loop interaction. Based on the authors' definitions, N=2 and M=1. Now, suppose A=B is the only neck interaction, and so k=1 and n=1. Using these values to perform a hypergeometric test (exactly how is actually not described by the authors) would lead to a wrong result. The correct result is that no matter how one edge is drawn, the interaction always involves a loop anchor (node B), and thus the p-value should be 1.

3. If a chi-square test is performed in the way described, how were the authors able to conclude that the neck neighbors are enriched in coding regions rather than depleted from them, since the chi-square test is only about dependency between two variables but not their direction of dependency?

Why are UTRs considered "coding regions"?

**Reviewer 3**

The authors performed thorough experiments to validate the performance and robustness of the methods. And also included additional discussions to suggest the biological relevance of the work. They've addressed all of my comments.

**Authors Response**

**Response to Reviewer #2**

*1a. The authors have compared their DSB-GNN method with two simple methods, LightGBM and RF, which is useful. However, their claim that they cannot compare with the more specialized methods proposed by Mourad et al. and Ballinger et al. seems not valid for a number of reasons. First, ChIP-seq data (required by these methods) are in general easier to obtain than Hi-C data (required by DSB-GNN). Second, some ChIP-seq data are in fact available for the NHEK cell line. Third, it may be possible to run those two methods with some data types omitted or set to constant/random values.*

**Response**: Thanks for pointing out the inadequate experiments in last revision and sorry for the impression that we would never wish to give. To address this, we have implemented these two methods and compared with our method.

First, we compared DSB-GNN with method proposed by Mourad et al. **We reproduced this method and got similar results** as reported in their paper: AUROC = 0.9678 when using a total of 10 kinds of data, and AUROC = 0.9437 when using CTCF and DNase-seq data only. As described in Mourad et al’s method, training dataset was constructed as follows (See *Fig. 1 for reviewer*): positive samples were DSB sites obtained by DSBCapture sequencing data, and negative samples were those non-DSB sites with genomic sequences that well matches in sizes, GC, and repeat contents of DSB sites. **Positive to negative ratio in this dataset was 1:1**. The authors discussed “The impact of controls on prediction” in their fifth result section, they pointed out that **good AUROC but lower PR curve** were obtained when **meeting higher class imbalance**. Overall, the whole genome, DSBs and non-DSBs are imbalanced. In our task, 76,604 5kb genomic regions are marked as DSBs by DSBCapture and other 466,019 genomic regions are non-DSBs. The ratio was ~1:6. We then performed Mourad et al’s method and DSB-GNN on this imbalance dataset. As showed in *Fig. 2 for reviewer*, Mourad et al’s method obtained **AUROC = 0.9351 and AUPR = 0.738** while DSB-GNN obtained **AUROC = 0.9251 and AUPR = 0.8224**. When training high imbalance datasets, AUPR was more suitable to evaluate model performance than AUROC. Thus, Mourad et al’s method was more advanced in studying specific genomic domain and DSB-GNN was more suitable for genome-wide study of DSBs.


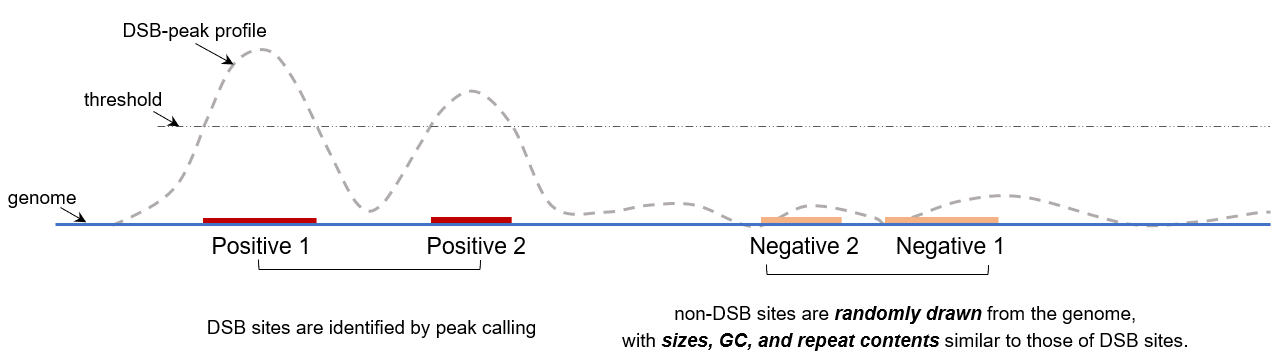
**Fig. 1** **for reviewer**. Dataset construction in Mourad et al. (2018).


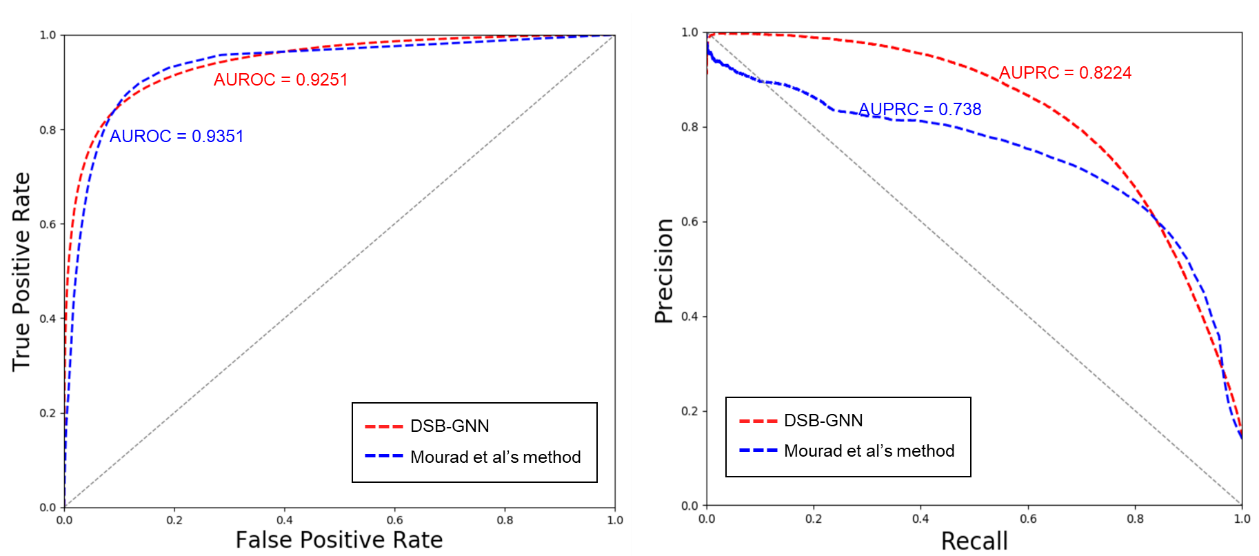


**Fig. 2 for reviewer.** Performance comparison using whole-genome imbalanced dataset.

Second, we compared DSB-GNN with method proposed by Ballinger et al. **We reproduced this method and got similar results as reported in their paper**: Pearson’s r=0.9228 when using a total of 21 kinds of data (*Fig. 3 for reviewer*, left), and Pearson’s r=0.8653 when using CTCF, DNase-seq and Hi-C data (*Fig. 3 for reviewer*, right). This method estimates the DSB frequency per 50kb region along the whole genome with a random forest regression model. We intended to systematically compare this method with DSB-GNN, but a direct comparison might not perfectly suit here due to the differences on task property and resolution, that is, Ballinger et al. aim to predict DSB frequency per 50kb (a real-valued rate) while DSB-GNN aims to predict the DSB existence per 5kb (an integer being either 0 or 1).


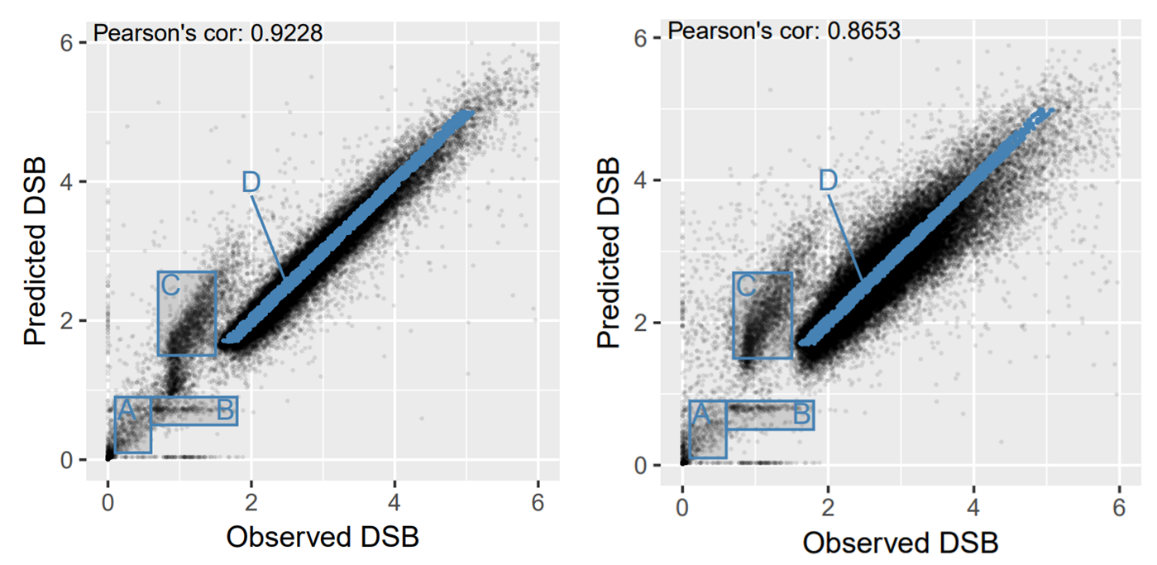


**Fig. 3 for reviewer.** Reproduced results for method of Ballinger et al.

All these three methods (Mourad et al, Ballinger et al and our DSB-GNN) have tried to leverage chromatin structure information into exploring DSBs. Mourad et al used DNA shape (predicted using R package DNAshapeR) plus transcription factor motif and achieved performance AUROC=0.8380. Ballinger et al used Hi-C data to measure the chromatin structure signal for each 50kb genomic bin and called DSBs with these signals, CTCF and DNase signals. DSB-GNN used Hi-C data to construct graph and identified DNA fragility associated structural unit (FaCIN) by GNN and GNN interpretation technique. All these works highlight the importance of chromatin structure in studying DSBs, while differ in the way the DNA fragility be modeled with chromatin organization reasonably incorporated.

*Also, the authors have not performed the ablation studies requested in the original comment. Therefore, even if DSB-GNN really has superior performance as compared to other methods, the relative contributions of the different components of the method (features, Hi-C network, self-attention mechanism, etc.) to the performance gain remain unclear.*

**Response**: We apologize for not providing sufficient information in last revision. To clarify the contributions of all components of DSB-GNN, we performed complete ablation experiments by removing each information once at a time. The scripts are provided in *Extended data 1 for reviewer*. Details are as following:

1. **For node sub-features**: we drop them individually.

2. **For Hi-C information**: simply dropping Hi-C network is not applicable, because we used Hi-C contact maps to construct the graph as the basic input and if the graph does not exist, the whole graph neural network could no longer work. We replaced the adjacency matrix (calculated from Hi-C data) with all zeros and all other elements remained the same.

3. **For components of the model design**: To test the contribution of self-attention mechanism, we compared DSB-GNN with a GCN-based model which has almost a same structure except **without self-attention mechanism**. Besides, we performed more ablation experiments on the model design to test the contributions of other components including edge coding, centrality encoding, positional encoding and Jumping Knowledge (JK) structure.

| Table.1 for reviewer.  Ablation results for removing each single component of DSB-GNN | | | |
| --- | --- | --- | --- |
| Information Type | | Specific Setting | AUC |
| node sub-feature | 3-mer+4-mer+5-mer+DNase+CTCF (DSB-GNN) | | 0.9251 |
|  | 4-mer+5-mer+DNase+CTCF | | 0.9208 |
|  | 3-mer+5-mer+DNase+CTCF | | 0.9187 |
|  | 3-mer+4-mer+DNase+CTCF | | 0.9154 |
|  | 3-mer+4-mer+5-mer+DNase | | 0.9168 |
|  | 3-mer+4-mer+5-mer+CTCF | | 0.8421 |
| Hi-C network | with real Hi-C network (DSB-GNN) | | 0.9251 |
|  | with all-zero adjacency matrix | | 0.9004 |
| Model components | with self-attention (DSB-GNN) | | 0.9251 |
|  | without self-attention | | 0.9118 |
|  | without edge encoding | | 0.9174 |
|  | without centrality encoding | | 0.9204 |
|  | without positional encoding | | 0.9218 |
|  | without JK structure | | 0.9192 |

Above results indicate that:

1. Regarding the contribution of different node features, the highest was DNase-seq data, and it was followed by CTCF ChIP-seq data and K-mer sequence feature.
2. Hi-C network plays an important role as it promotes AUROC with nearly 2.5 percentages when it is already over 0.9.
3. Different components of model design have varying levels of contributions. Among them, self-attention mechanism, edge encoding and JK structure play more important roles.

Thanks for the reviewer’s advice in improving the quality of our method, and we have added these results into Methods section in the revised manuscript.

*1b,c. The authors emphasized that their main finding is the bottleneck pattern, which is "a universal form of how the fragility of a piece of DNA might be affected (or affect) by the whole genome in three dimensional space. In brief, FaCIN helps to truly dissect the mechanisms driving DNA fragility under the 3D genome". Exactly how bottleneck is defined remains unclear after this revision (see 2a below). What this pattern implies regarding chromatin organization is also unclear. Does it simply mean that if a region is fragile to DSB, other regions close to it in the 3D space are also likely fragile? Or are there additional conditions required such as the detailed connections and chromatin states of those other regions?*

**Response**: Thanks for the reviewer to point out where we have fallen short in last revision. We split this comment into two parts.

The first part is about the clear definition and meaning of bottleneck. As it also presents as a major problem in *Comment* *2a* below, we combined the answers into an integrated one. **Please refer to the response *2.a* for more information**.

The second part consists of two specific questions.

Q1*: Does it simply mean that if a region is fragile to DSB, other regions close to it in the 3D space are also likely fragile?*

**Answer**: The “other regions” are not those regions that are simply close to a DSB site. In our work, we found that regions that were identified as neck neighbours in FaCIN are fragile to DSB (*Fig. 4 for reviewer*). Specifically, for a given DSB site, its neck neighbour has a 22% probability of developing DSB and this is significantly higher than the background.


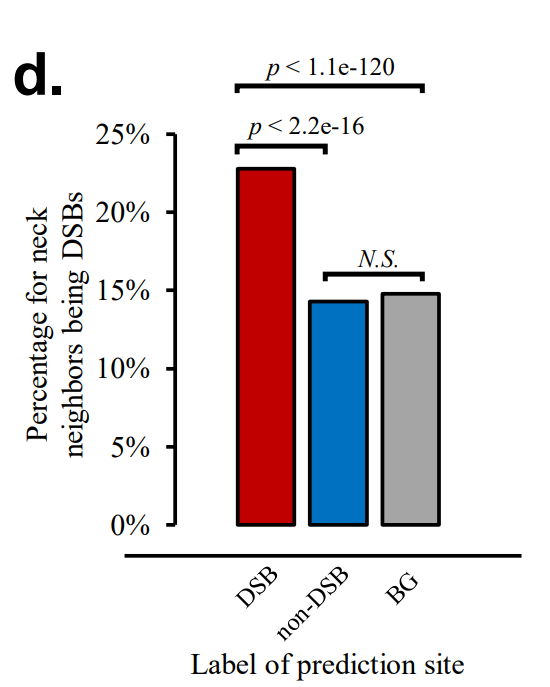


**Fig. 4 for reviewer.** Original Fig. 4d in the manuscript.

These neck neighbours are not necessarily those regions most close to a DSB site (*Fig. 5 for reviewer* and *Table.2 for reviewer*). As discussed earlier, the neck neighbours do not need to be those regions most proximal to prediction site in 3D spatial distance (measured by higher contacts) or 1-D genomic distance (measured by bases in between). From this point, FaCIN suggests that DSB mechanism might be more complicated than it appears.


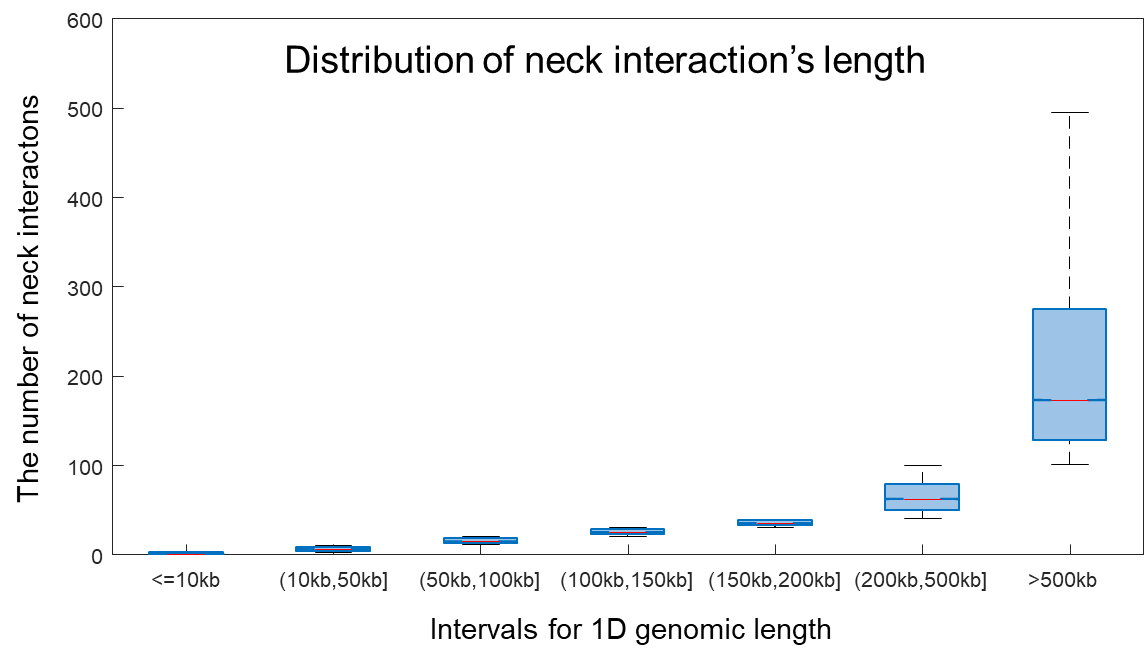


**Fig. 5 for reviewer.** Distribution of 1D genomic distance from neck neighbour to prediction site (e.g., the length of neck interaction). Each box represents the number of interactions whose 1D genomic length fall into the corresponding interval. Different intervals for interaction length are on the horizontal axis and the number of interactions on the vertical.

**Table.2 for reviewer.**

| Interval (kb) | (0,10] | (10,50] | (50,100] | (100,150] | (150,200] | (200,500] | (500, max] |
| --- | --- | --- | --- | --- | --- | --- | --- |
| Percentage | 4.88% | 17.26% | 15.48% | 10.88% | 8.08% | 24.14% | 19.29% |

Q2*: Or are there additional conditions required such as the detailed connections and chromatin states of those other regions?*

**Answer**: According to our observation on FaCIN’s bottleneck pattern, there are additional conditions for such a DSB co-occurrence. Given a region that is vulnerable to DSB, if another region nearby tends to develop DSB as well, this region needs to satisfy: (i) it has direct interactions with the DSB region and (ii) if the direct interaction is removed, the status of the original DSB region will change. Actually, above conditions can be interpreted as the definition of neck neighbour: (i) a node whose shortest path to the prediction site on Hi-C contact graph is only 1 step and (ii) remove the edge between this node and prediction site will affect DSB-GNN’s decision-making and leads to a variation of prediction score that ranks higher (top 10) than that can be led to by removing other nodes.

*2a. In the mathematical definition given, the meanings of |S| and |N(S)| are not stated. Assume |S| means the number of nodes in set S, and N(S) means the set of neighbors of the nodes in S excluding the nodes in S. Then there are many types of subgraphs that match this definition. For example, any connected component is a bottleneck, because the nodes in a connected component are not connecting to any other nodes and thus |N(S)|=0. In the most extreme case, if all genomic regions are connected through chromatin interactions, the whole genome together is a bottleneck. It is therefore unclear conceptually what insights about effects of genome architecture on DSB fragility the bottleneck pattern is supposed to provide.*

*The authors do not seem to have given the mathematical definition of bottleneck in the revised manuscript, or performed actual calculations to show that the subgraphs they frequently found from the FaCINs are bottlenecks.*

*The description of Hall's Theorem in the response letter seems completely irrelevant -- the Hi-C contact graph is not bipartite in general, and whether there is a perfect matching between nodes in the Hi-C contact graph is unrelated to the current work.*

*In the response letter, is said that "Our bottleneck pattern has a similar extended meaning to that of Hall's theorem, which slightly differs in that the neck interactions have less elements than neighbours." How is the meaning extended? How is Hall's Theorem relevant here?*

**Response**: We appreciate for the reviewer’s generosity of attention and please accept our apology for having caused a serious miscommunication involving the Hall's theorem quoted in our last reply. We described this theorem with the intention to show how we were inspired by it to use the word “bottleneck”, rather than providing it as the exact mathematical definition of FaCIN.

**First, let us answer the questions arise from our ambiguous quote about Hall’s theorem.**

1. The meanings of |S| and |N(S)|?

Answer: They are just ordinary denotations used define the number of nodes in two parts of a bipartite graph. They actually have no relationship with Hi-C interaction.

1. How is the meaning (of Hall's theorem) extended? How is Hall's Theorem relevant here? Answer: The original meaning of bottleneck in Hall's theorem involves a comparison between the number of two group of nodes; in our case, we observed the number of 2-hop neighbours are generally greater than that of 1-hop neighbours in FaCIN. We admit it might be farfetched to say them somewhat alike, but we really got inspiration from this theorem to name FaCIN’s topological pattern.

**Second, we give the exact definition of the bottleneck:**

For each node, its FaCIN is a connected graph denoted as **G_Bottleneck = {*N*, *E*}**, where ***N*** represents the node set and ***E*** represents the edge set. Denote an edge (undirected) connecting a pair of nodes ***n****_i_* and ***n****_j_* as ***e***_(_*_i,j_*_)_. Denote the prediction site in FaCIN as ***n****_p_*.

For prediction site ***n****_p_*, we first measured the hops of its neighbours by calculating the shortest distance from ***n****_p_* to its neighbour. For example, if prediction site ***a*** interacts with node ***b***, node ***b*** interacts with node ***c***, but node ***c*** does not interact with prediction site ***a***. Shortest distance between ***a*** and ***b*** is 1 and ***b*** is a 1-hop neighbour of ***a***. Similarly, shortest distance between ***a*** and ***c*** is 2 and ***c*** is a 2-hop neighbour of ***a***. Here, we focus on 1-hop neighbours denoted as ***N****_1h_*(***n****_p_*) and 2-hop neighbours denoted as ***N****_2h_*(***n****_p_*).

From above calculation, ***E*** edge set is defined as no more than 10 most influential edges that are determined via GNNExplainer. Each edge ***e***_(_*_i,j_*_)_ in ***E*** needs to satisfy:

***n****_i_*∈{***n****_p_*, ***N****_1h_*(***n****_p_*), ***N****_2h_*(***n****_p_*)}

***n****_j_*∈{***n****_p_*, ***N****_1h_*(***n****_p_*), ***N****_2h_*(***n****_p_*)}

i ≠ j

$\boldsymbol{\Delta v}$ ranks in Top10, if ***e***_(_*_i,j_*_)_ removed

$\boldsymbol{\Delta v}$ is calculated by GNNExplainer and represents the difference of prediction scores before and after removing an edge, and the difference value is positively correlated to the contribution of this edge to a correct prediction. If a node whose total interactions on Hi-C contact graph are less than 10, its ***E*** edge set includes just these interactions.

***N*** node set includes those nodes connected by edges in ***E***.

**Third, we performed actual calculations supporting the bottleneck:**

To begin with, we introduce the concept of **betweenness** **centrality**, which indicates how often a node appears on the shortest path between any random node pairs. For example, in *Fig. 6 for reviewer*, the betweenness centrality of the yellow node for the red-green node pair is 2/3 = 0.667, as the number of shortest paths between red-green node pair is 3 and among them the yellow node appears twice. **For this metric, if a node has a higher value, it has a stronger mediating or bridging role**.


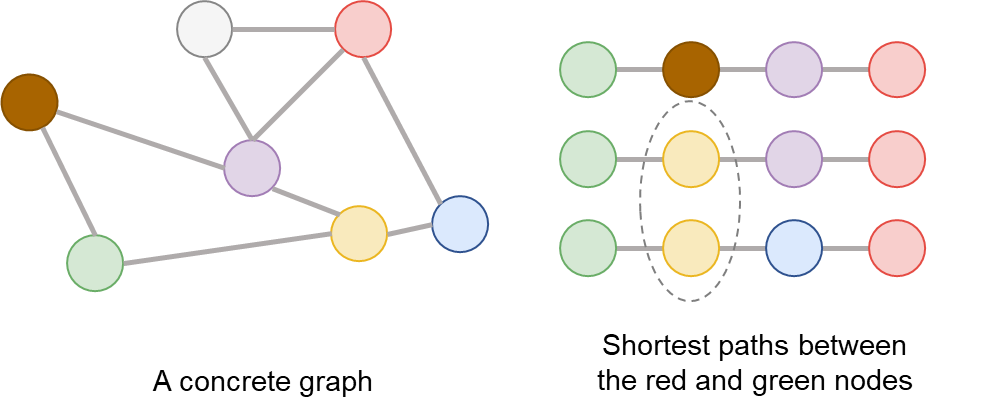


**Fig. 6 for reviewer.** An example to illustrate how to calculate betweenness centrality.

In brief, betweenness centrality actually measures how well a node is connected across the whole graph. We calculated the betweenness centrality of 1-hop and 2-hop neighbours to see how often they appear on the shortest path between other nodes and prediction site. **We found that the 1-hop neighbours have on average a much higher betweenness centrality** (*Fig. 7 for reviewer*)**, meaning that 1-hop neighbours are in bottleneck positions which have more control over the information in FaCIN. This is also the reason why we named the 1-hop** **neighbours as neck neighbours** and the interactions between prediction site and 1-hop neighbours as neck interactions.


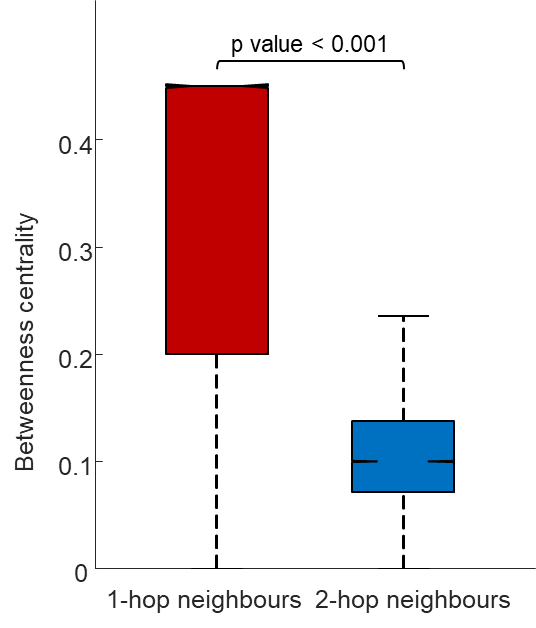


**Fig. 7 for reviewer.** Comparison for betweenness centralities of

1-hop neighbours and 2-hop neighbours (t-test).

**Fourth is regarding to biological insights:**

We know that genes, chromatin structures and chromatin modifications, they all convey biological information. This information is further organized in a hierarchical structure whose features are ordered, constrained and related [*Searls, David B. Nature (2002)*; *Zhao, Shuai et al. Nature reviews. Cancer (2021)*]. As for DSB, the biological information can be from various sources. For example, the torsional stress on DNA strands induced by replication or transcription often lead to DSBs [*Uusküla-Reimand, Liis et al. Genome biology. 2016*]. Currently, it is widely accepted that 3D genome has an effect on DNA fragility but how can the chromatin organization affect DSBs in general is unknown. The bottleneck pattern suggests that the fragility of a piece of DNA is associated with other genomic regions in a cascading manner, that is, the prediction site directly communicates with neck neighbours of small number and neck neighbours gather biological information from many more genome regions at distance. Besides, FaCIN’s size varies from kilobase to megabase, indicating the DNA fragility-associated genome organization might exceed the space of ordinary TAD level. This also helps us to understand long-range chromatin interactions from a new perspective.

Together, thanks for the reviewer’s patience and constructive advice, and we have added these description and discussion in the revised manuscript.

*The fancy figure with two bottles sharing the same neck region is very confusing -- Does the definition of a bottleneck requires two bottles?*

**Response**: Thanks for this comment as a timely reminder. The definition of bottleneck only involves one bottle and we have modified the corresponding figures in both the main text of manuscript (*Fig. 8 for reviewer, part of the original Fig. 2a*) and the additional file. Hope it provide a clear illustration of above concepts, and we have updated the corresponding parts in the revised manuscript.


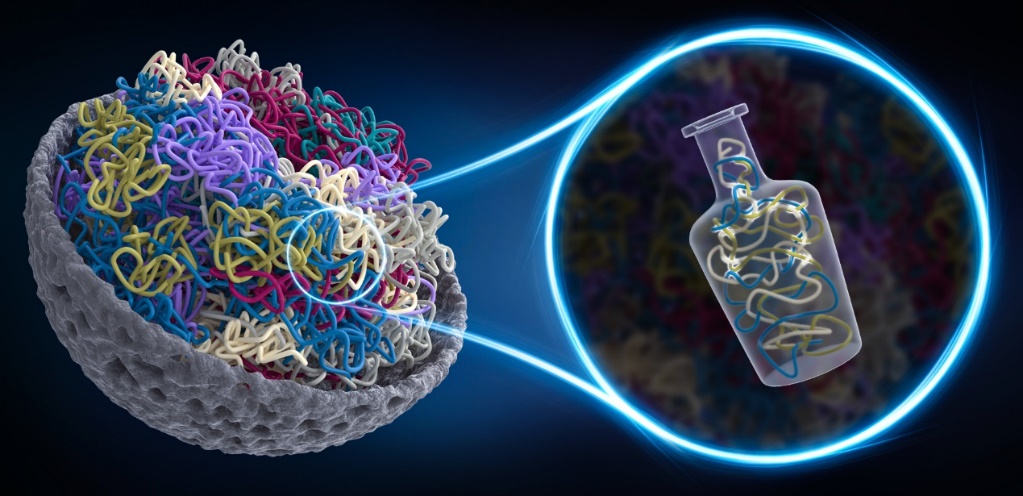


**Fig. 8 for reviewer.**

*2b. A long explanation is provided but it seems relevant to the comment, which is about the rationale for calling direct interactions involving the prediction site "neck interactions".*

**Response**: Thanks for the reviewer’s patience. We feel sorry that our previous description and last reply was poorly organized. We merge the last and the current comments and answer the questions involved as below:

Q1: *questions raised in last comment: “So, no matter which genomic bin is taken as the prediction site, its direct interactions in the FaCIN are always called neck interactions?”*

Answer: Yes, just as the reviewer pointed out. Neck interactions are those interactions directly contact prediction site in FaCIN.

Q2: *The rationale for calling direct interactions involving the prediction site "neck interactions”.*

Answer: After the discussion in **2.a**, the third and fourth sections, it is straightforward that we name 1-hop neighbours as the neck neighbours since they have higher betweenness centrality and are in bottleneck positions in FaCIN.

*3. The authors have not responded to the request of comparing the results of different numbers of GNN layers.*

**Response**: We apologize for not adequately addressing this question. In last revision, the time was tight for us to address all comments from three reviewers and due to the limited computing resources, we failed to meet this request. We now have coordinated more computing resources to make up this test.

We trained different models with number of GAT layers ranging from 1 to 5. As shown in *Table. 2 for reviewer*, the 3-GAT layer model achieves the highest AUROC. Differences in performance between models are not large. Good performance benefits from attention mechanism and JK implementation in our model. AUROC decreases when using GCN or dropping JK. All these experiments have been added in the revised manuscript.

| **Table. 2 for reviewer.** | | |
| --- | --- | --- |
| **Setting** | **Number of layers** | **AUROC** |
| GAT with JK | 1 GAT layer | 0.9184 |
|  | 2 GAT layers | 0.9231 |
|  | 3 GAT layers (DSB-GNN) | 0.9251 |
|  | 4 GAT layers | 0.9238 |
|  | 5 GAT layers | 0.9214 |
| GCN with JK | 1 GCN layer | 0.9089 |
|  | 2 GCN layers | 0.9118 |
|  | 3 GCN layers | 0.9073 |
| GAT without JK | 1 GAT layer | 0.9097 |
|  | 2 GAT layers | 0.9116 |
|  | 3 GAT layers | 0.9134 |
|  | 4 GAT layers | 0.9123 |
|  | 5 GAT layers | 0.9108 |

*4. The authors argue that the robustness of their model "was not affected by different Hi-C normalization methods and this is not surprising because in our model the normalization is no longer a preprocessing step but partly transferred to the GNN model, as the graph weights derived from raw interactions are constantly updated during training." Apparently the authors have mixed up two different concepts, namely Hi-C data normalization, for removing biases, and learning weights in a GNN, for deriving features useful for predicting DSBs.*

**Response**: We feel sorry that this misunderstanding seems to arise from our imprecise usage of term “weight”. **We meant to use weight to represent Hi-C information that is constructed into a weighted graph as the input of DSB-GNN model, rather than the neural network parameters**. We understand that Hi-C data normalization is often required since many factors in Hi-C experiment can affect the contact count.

Regarding to “the graph weights derived from raw interactions are constantly updated during training”, we actually intended to describe how the graph (i.e., the input constructed from Hi-C data) undergoes changes in terms of its node embeddings and edge embeddings. During iterative training, the parameters of DSB-GNN change, and the embeddings of the graph in data flow also change. More details about how the changes of graph embeddings take place during iterative model training are provided in *Extended file 1 for reviewer* to save space in our response.

Experiments showed that the performance of DSB-GNN with raw Hi-C data is comparable to that of DSB-GNN using normalized Hi-C data via KR or ICE (*Table. 3 for reviewer*), indicating that DSB-GNN is capable to deal with Hi-C data even with noises or biases. Please refer to the response to *5c comment* for more information as the questions have some overlap.

Thanks for the reviewer’s reminding again. We have carefully contextualized the key words we used and rewritten the corresponding part in the manuscript.

| **Table. 3 for reviewer** | | | |
| --- | --- | --- | --- |
| Normalization | RAW | KR | ICE |
| AUROC | 0.9251 | 0.9229 | 0.9222 |

*5b. With a bin size of 5kb, most chromatin interactions would not be considered proximal according to the authors' definition (of <10kb), and thus this definition is not suitable in this context. The key question, which the authors have not answered, is whether patterns in the Hi-C contact graph related to DSBs are really due to non-trivial 3D genome structures or simply due to 1D genomic closeness. A simplistic binary classification of interactions into "proximal" and "distal" seems inappropriate here.*

**Response**: As the reviewer pointed out, we admit that only using 10kb to distinguish proximal interactions from distal ones is insufficient. However, there seems to be no uniform standard to make such a classification. For example, Holgersen, Erle M et.al (*Nature protocols*, (2021)) defines those interactions ≥ 10kb as distal while Rao et.al (*Cell*, (2014)) uses 20kb as a threshold and so on. We here calculated the distribution of 1D genomic distance from prediction site to 1-hop neighbours (i.e., neck neighbours) and 2-hop neighbours, shown in *Fig. 9 for reviewer* and *Fig. 10 for reviewer* below. Their percentages are shown in *Table. 4 for reviewer.*


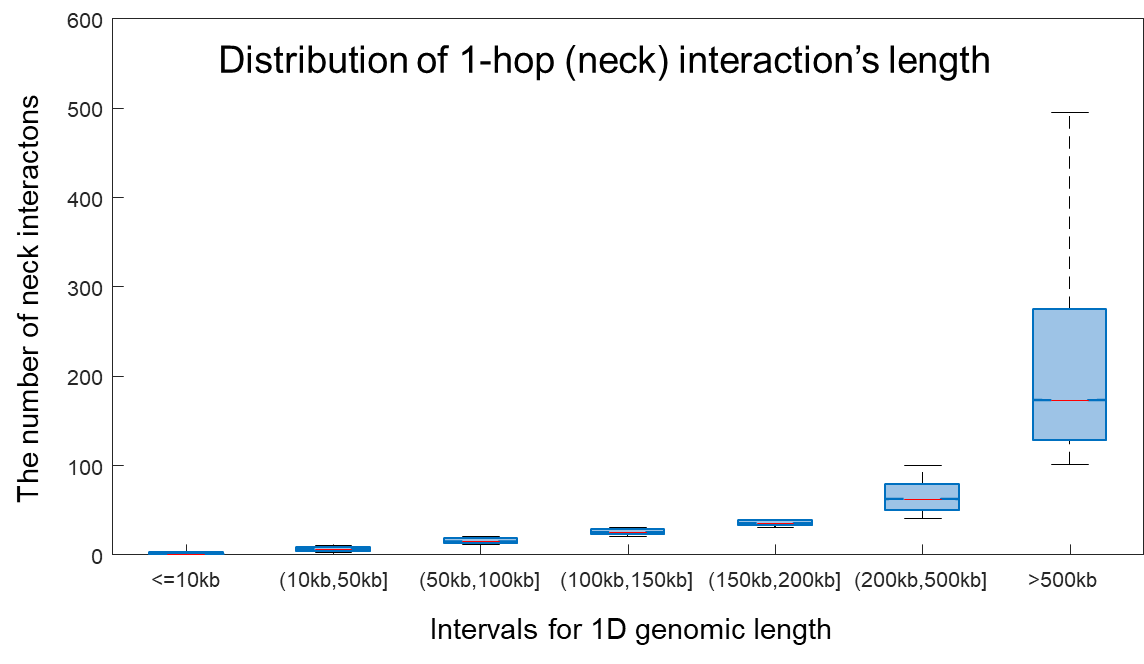


**Fig. 9 for reviewer.** Distribution of 1D genomic distance from 1-hop (neck) neighbour to prediction site (e.g., the length of 1-hop interaction). Each box represents the number of interactions whose 1D genomic length fall into the corresponding interval. Different intervals for interaction length are on the horizontal axis and the number of interactions on the vertical.


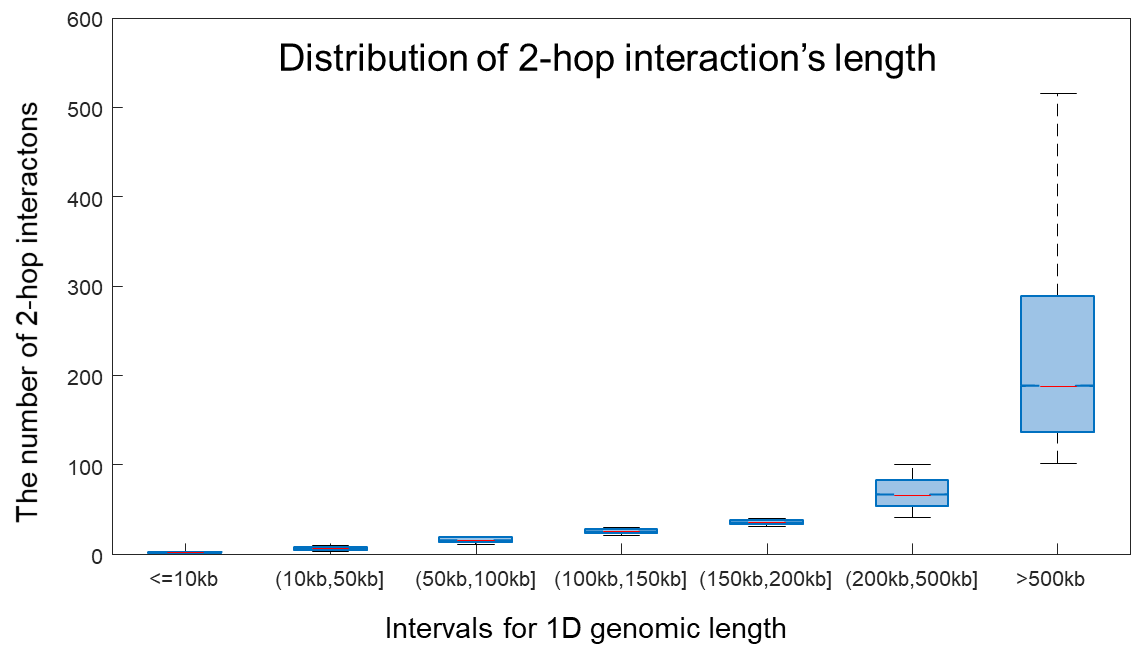


**Fig. 10 for reviewer.** Distribution of 1D genomic distance from 2-hop neighbour to prediction site (e.g., the length of 2-hop interaction). Symbols have the same meaning as in Fig. 9 for reviewer.

**Table.4 for reviewer.**

| Interval (kb) | (0,10] | (10,50] | (50,100] | (100,150] | (150,200] | (200,500] | (500, max] |
| --- | --- | --- | --- | --- | --- | --- | --- |
| Percentage  of 1-hop | 4.88% | 17.26% | 15.48% | 10.88% | 8.08% | 24.14% | 19.29% |
| Percentage  of 2-hop | 1.56% | 6.23% | 7.50% | 6.99% | 6.47% | 28.55% | 42.69% |

From above results, we can learn that **FaCIN includes both distal and proximal interactions.** Even if we set the threshold to be 100kb, FaCIN’s pattern still shows rich information from distal interactions under 3D genome. For example, neck interactions that span over 100kb account for 62.39% of the total and this percentage for 2-hop interactions is as high as 84.7%.

We agree that to distinguish proximal interactions from distal interactions is important since the significance of an interaction might be distorted by linear proximity. While in our work, FaCIN aims to identify a universal form of chromatin structural unit concerning with DNA fragility, which requires the raw data to be as global as possible. To this end, it is not perfectly applicable to our task to only focus on distal interactions.

Thanks for the reviewer’s suggestions again and we have added above information in the manuscript to show that FaCIN’s pattern contains rich information from 3D genome structures.

*5c. By using raw counts, there could be noise and biases in the data that affect the analysis.*

**Response**: Actually, as the reviewer pointed out, there could be noise and biases in Hi-C raw counts data. For general Hi-C down-stream analysis, such as TAD and loop identification, using KR- or ICE- normalized Hi-C data is more suitable than raw counts data. One could not have identified reliable loops without removing the biases in Hi-C data as the definition of loop directly depends on the quality of Hi-C data. However, for DSB-GNN, the ground truth (DSB or non-DSB label) derives from DSB-Capture, which is another experimental technique unrelated to Hi-C experiment. Therefore, the noise and biases in Hi-C data would not do harm to the reliability of DSB label. As the ground truth is fixed, whether a model is affected by the noise or biases of input will manifest in its performance, for example, the performance of DSB-GNN will drop if the data noise or biases cannot be overcome.

Thus, to test whether Hi-C data normalization is required in DSB-GNN predicting DSB, we trained DSB-GNN models with raw counts data, KR-normalized data and ICE-normalized data, respectively. AUROCs are very close for these three models (*Table. 3 for reviewer*). Neural networks are powerful to extract effective information from noisy data and exhibited the ability to remove feature engineering process. Using raw counts in our DSB-GNN does not impair DSB prediction performance. In addition, current normalization methods all make assumptions that certain factors in Hi-C experiments are responsible for the biases or that the biases are scalar, multiplicative and one-dimensional and so on. But whether these assumptions are applicable to the context of DSB is not actually known.

Thanks for the reviewer’s advice, and we have added this result and discussion in the revised manuscript.

| **Table. 3 for reviewer** | | | |
| --- | --- | --- | --- |
| Normalization | RAW | KR | ICE |
| AUROC | 0.9251 | 0.9229 | 0.9222 |

*6. The description of the masking procedure is incomplete. For example, what is the initial masking matrix? What is the loss exactly? What does it mean by "the goal of identifying the optimum subgraph Gs(v) and feature set Xs(v)"? How are the masking coefficients adjusted?*

**Response**: Many thanks in advance for the reviewer’s time and patience! We response this question one by one as following. The entire masking procedure is very detailed and will take up a lot of space here, we also provided a separated file for the entire process (Please see *Additional file 2 for reviewer*).

Q1: *What is the initial masking matrix?*

Answer: Take edge mask as an example, the key computation in masking procedure, can be formulated as: $A_{c}\odot\sigma(M)$, where $A_{c}$ denotes the associated binary adjacency matrix of computation graph *G_c_* (a subgraph which includes all information for a model to predict a node, about its meaning please refer to section 2.1 in *Additional file 2 for reviewer*), $M$∈ℝ ^n×n^ denotes a real-valued mask matrix whose parameters that GNNExplainer aims to learn, $\odot$ denotes element-wise multiplication, and $\sigma$ denotes the sigmoid that maps the mask matrix to [0, 1]^n×n^. Similarly, there also exist a *F* which denotes the mask matrix for node features. The initial masking matrix refers to the randomly initialized $M$ or *F*. More details can be found in sections 2.3 and 2.4 in *Extended file 2 for reviewer*.

Q2: *What is the* *loss exactly?*

Answer: The loss is the cross entropy between the label and the prediction after masking as:$loss=CrossEntropy\left( \Phi\left( G_{c}\boldsymbol{,}M \right),Label \right)$.

Q3: *What does it mean by "the goal of identifying the optimum subgraph Gs(v) and feature set Xs(v)"?*

Answer: The optimum subgraph Gs(***v***) means the subgraph of the computation graph (neighbourhood concerned with a node defined by GNN model), which is composed of those edges that have highest mutual information *MI* with the node ***v***’s prediction. Likewise, the optimum feature set Xs(***v***) is a subset of node features limited to Gs(***v***) that are most important measured by *MI*. to identify Gs(***v***) and Xs(***v***) is the goal of GNNExplainer and therefore is also the optimizing objective of masking procedure.

Q4: *How are the masking coefficients adjusted?*

Answer: Masking parameters (coefficients) are adjusted through optimizing the loss formulated in answer of Q2. The specific optimizer is Stochastic Gradient Descent (SGD).

Again, thanks for the reviewer’s remind, we have added above description including initial masking matrix, loss function, coefficients adjusted etc in the Methods of our revised manuscript.

*7. The authors claim that they "tried to keep the model's performance as independent as possible from threshold parameters".* *In fact, requiring a contact count of at least 2 is exactly a demonstration of picking an arbitrary threshold and the performance could be affected by this choice. It is thus not clear why the authors believe they have reduced the dependency of their method's performance on thresholds.*

**Response**: Sorry for this unthoughtful claim without adequate experiments on threshold selection for model performance. We required the raw contact count to be at least 2, mainly from the need to reduce graph size and density while improve computational efficiency. To this end, we calculated the distribution of raw contact values (See *Fig. 11 for reviewer*), and selected 2 as threshold. After such a filtering, the regions corresponding to the graph nodes cover 92.52% of the whole genome including 99.14% of DSB sites identified by DSBCapture; the total interactions sum up to about 26 million and graph sparsity on different chromosomes range from 0.002 on chr 2 to 0.013 on chr 21; the running time of DSB-GNN is 2.76 hr. However, as the reviewer pointed out, we did not really test the effect of this choice on model performance.


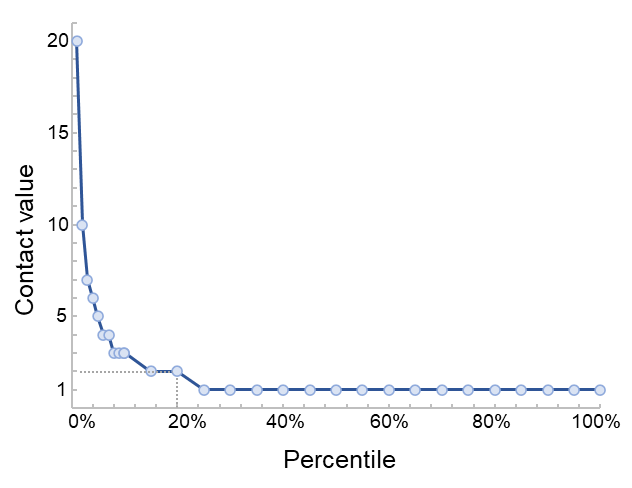


**Fig. 11 for reviewer.** Distribution of Hi-C raw contact values on NHEK dataset.

To address this point, we made up the test for different thresholds, that is, contact count ≥1, ≥2, ≥3, ≥4, ≥5. Interaction numbers under each threshold are showed in *Fig. 12 for reviewer*. AUROC, running time and GPU memory required under each threshold are listed in *Table. 5 for reviewer*. On one hand, AUROC of contact count ≥2 is over 1% higher than those of contact count ≥3, ≥4 , ≥5. On the other hand, due to the dense interactions under contact count ≥1, the running time of contact count ≥1 is longer and GPU memory required is much larger.


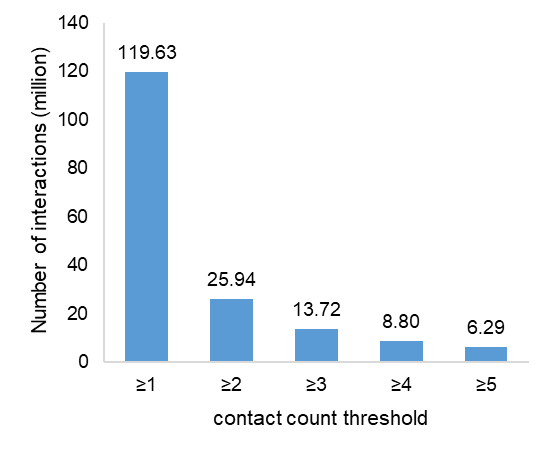


**Fig. 12 for reviewer.** Number of interactions under different thresholds.

**Table. 5 for reviewer**

| Configuration of local device  CPU: Intel(R) Xeon(R) Silver 4214@2.20GHz, 12 cores  GPU: RTX 3090, 24G | | | |
| --- | --- | --- | --- |
|  | AUROC | GPU memory | Running time (hr) |
| contact count ≥1 | 0.9248 | 12GB | 3.75 |
| contact count ≥2 | **0.9251** | 8GB | 2.76 |
| contact count ≥3 | 0.9147 | 6GB | 2.61 |
| contact count ≥4 | 0.9123 | 5GB | 2.47 |
| contact count ≥5 | 0.9108 | 3GB | 2.33 |

Thanks for the reviewer’s constructive suggestion, we have added above experiments in our revised manuscript. In current data source, selecting contact count ≥2 as threshold is satisfactory. For other Hi-C data (for example, with higher resolution), different contact count thresholds may be more effective to improve model performance.

*8a. If a FaCIN is a connected subgraph that covers "at maximum a two-hop region", why do some motifs discovered (shown in "Fig. 11 for reviewer") involve nodes that are more than four hops away from each other?*

**Response**: as the reviewer pointed out, FaCIN is a connected subgraph that covers at maximum a two-hop region. Motifs showed in last response were also constructed by nodes within two-hops. We think what is misleading is the unclear definition of FaCIN as discussed in comment 1b,c and 2a. Node hops were defined as the steps from query node to center node (prediction site).For example, in *Fig. 13 for reviewer*, step from node *b* to node *a* is 1, so the hop of node *b* to node *a* is 1, and similarly, the hop of node *c* to node *a* is 2. From all the nodes within 2 hops and connections between them, FaCIN was defined as the important nodes and important connections detected by GNNExplainer. In this scenario, more 2 hop nodes could exist in one FaCIN while their 1 hop node not exists. For example, 2 hop nodes *e*, *f*, *g* are in FaCIN while their 1 hop node *m* not. A subgraph of this FaCIN is *a*-*b*-*c*-*d*-*e*-*f*-*g*, which is a form of cascade motif.


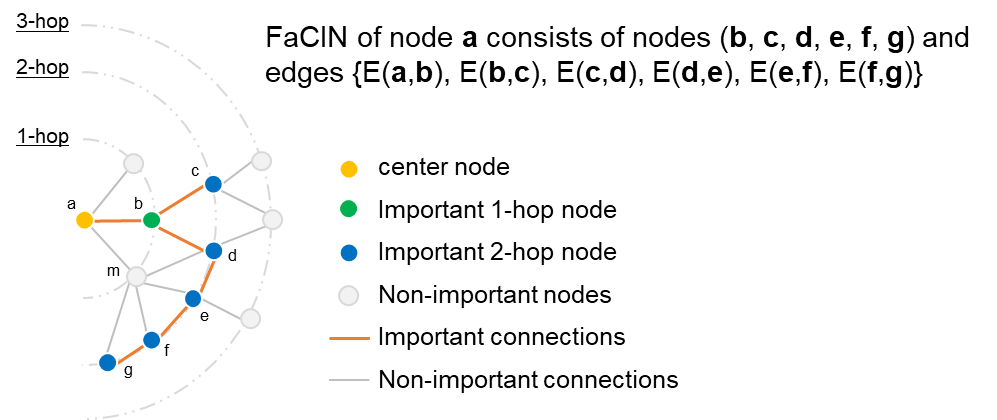


**Fig. 13 for reviewer.**

To describe the motifs more clearly, we have provided sufficient description of FaCIN and examples of cascade motifs (See *Fig. 14 for reviewer*) in the revised manuscript.


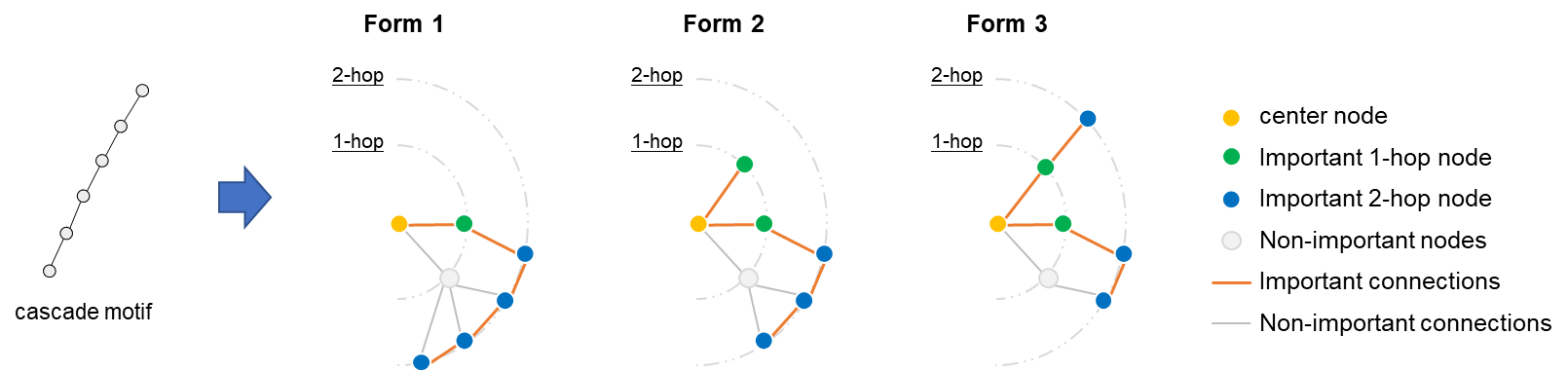


**Fig. 14 for reviewer.**

*9a. The definition for chromatin loops given is incomplete. How is the significance of proximity determined? Is closeness adjusted for 1D distance or not? It is not clear which subset of Hi-C contacts are considered chromatin loops based on this definition.*

**Response**: Thanks for the careful review, and we are sorry for the confusion caused by the unclear definition of chromatin loops.

To address it, we clarified the detailed definition of chromatin loops based on two classic literatures (*Rao, S.S., et al., Cell, 2014. 159(7):p.1665-80* and *Jerkovic et al., Nat Rev Mol Cell Biol, 2021. 22(8):p511-528*) in 3D chromatin structure studies. “**A chromatin loop occurs when stretches of genomic sequence that lie on the same chromosome (configured in cis) are in significantly closer physical proximity to each other than to intervening sequences**”. In other words, chromatin loop is a structure in which two loci located in cis are closer, in 3D space, than intervening loci or other neighbouring loci. This definition implies that chromatin loops manifest as local peaks in a proximity ligation dataset, which occur between two points whenever they interact with each other significantly more than with random points in their neighbourhood. In Hi-C data, the typical peak reflects a two-to-five-fold increase in contact frequency at a peak pixel, and tends to decay in a roughly circular fashion across an “interaction region” in the heatmap. These focal peaks imply that two loci are in close proximity, but that this proximity relationship is not shared by intervening loci. As such, they indicate the presence of chromatin loops.

Rao et al. developed HiCCUPS (Hi-C Computational Unbiased Peak Search) to identify DNA loops, and this algorithm explained **how is the significance of proximity determined**. Detailed information is provided in *Extended file 3 for reviewer*. Briefly, the algorithm is divided into the following steps:

a. The “observed over expected” (O/E) matrix calculation: **The O/E matrix is the result of closeness adjusted for 1D distance**. This uses a genome-wide 1D model to account for the increased number of contacts seen at short distances due to random polymer interactions driven by one-dimensional genome proximity. (For more details, please see 1^st^ section in *Extended file 3 for reviewer*).

b. Local expected value calculation: Based on O/E matrix, HiCCUPS calculates multiple local expectation values for every pixel in order to rule out the possibility that another local feature, such as the edge of a domain, could lead to a spurious, peak-like enrichment. (For more details, please see 2^nd^ section in *Extended file 3 for reviewer*).

c. **Multiple hypothesis testing**: HiCCUPS uses a modified Benjamini-Hochberg FDR control procedure, dubbed “λ-chunking” by binning pixels into “hypothesis classes” based on their expected value, which is specifically designed to work with the unique statistical structure of Hi-C data and rigorously enforces thresholds of local enrichment. (For more details, please see 3^rd^ section in *Extended file 3 for reviewer)*.

**The significance of proximity is determined by the number of contacts in the pixel and the 10% FDR threshold on each of their four local expected values**. The pixels were identified as locally enriched if the number of contacts in the pixel was greater than the 10% FDR threshold on each of their four local expected values. Because this enrichment was assessed for each neighbourhood separately, the resulting procedure is stringent and markedly improves peak-call reliability.

*9b. Due to the unclear definition of chromatin loops, it is difficult to evaluate whether having 19,632 loops genome-wide is reasonable.*

**Response**: Sorry for the unclear description of 19632 loops used in our study. These 19632 loops were directly obtained from 3D Genome Browser (<http://3dgenome.org>) provided by Peakachu method (*Salameh, T.J., et al., Nat Commun, 2020. 11(1):p.3428*). Peakachu uses the definition of chromatin loops from the above two literatures (*Rao, S.S., et al., Cell, 2014. 159(7):p.1665-80* and *Jerkovic et al., Nat Rev Mol Cell Biol, 2021. 22(8):p511-528*), and authors of Peakachu method have conducted independent validation to demonstrate the validity and reliability of Peakachu in predicting genome-wide chromatin loops from genome-wide contact maps. In this literature, the authors performed Peakachu on NHEK cell line and provided these 19632 genome-wide chromatin loops. Please see 4^th^ section (Validation of Peakachu) in *Extended file 3 for reviewer* for more details.

In the past two years, Peakachu has been cited in 22 articles including Nature Biotechnology (*Deshpande, A.S., et al., Nat Biotechnol, 2022.40(10):p.1488-1499*), Nature Methods (*Wang, X., et al., 2021.18(6):p.661-668*), Journal of Hematology & Oncology (Deng, S., et al., J Hematol Oncol, 2022. 15(1):p.49) and Genome Biology (lyyanki, T., et al., Genome Biol, 2021. 22(1):p.105) etc.

Hope these details together with above definition in Response 9a can help confirm our reasonable use of 19632 chromatin loops.

*9c. If the functional role is not a requirement for a loop, why is it still part of the definition text within the quotation marks given in the response to 9a?*

**Response**: as the reviewer pointed out, **functionality is not necessary to define a chromatin loop**.

Our last response of loop definition “Loops are pairs of loci that show significantly closer proximity **which tend to have special functions such as enhancer-promoter** (E-P) linkage” comes from the previous study (*Rao, Suhas S P et al. Cell (2014)*). We think the authors tried to express that loops tend to enrich in function elements than expected as they pointed out that “2854 peaks with promoters and enhancers in our GM12878 annotation of 9448 peaks (30%), as opposed to 653 (7%) in a comparable random annotation”.

Thanks for the reviewer’s careful consideration, and to avoid this misunderstanding, we revised the definition of chromatin loops as showed in Response 9a.

*10. There are some issues about the statistical tests:*

*1,2. In the hypergeometric test, is the p-value defined as the probability of having exactly k interactions or at least k interactions? The authors' response seems to suggest the former.*

*The calculations performed by the authors mix the counting of nodes and edges together. Consider a graph A=B-C, where "=" means a loop interaction and "-" means a non-loop interaction. Based on the authors' definitions, N=2 and M=1. Now, suppose A=B is the only neck interaction, and so k=1 and n=1. Using these values to perform a hypergeometric test (exactly how is actually not described by the authors) would lead to a wrong result. The correct result is that no matter how one edge is drawn, the interaction always involves a loop anchor (node B), and thus the p-value should be 1.*

**Response**: As the reviewer pointed out, our hypergeometric test was not clearly described, and our claim “We found that the neck interactions were significantly enriched in loop anchors” was wrong by mixing nodes and edges together. What we intended to say is “We found that the neck interactions were significantly enriched in loop interactions”, not “loop anchors”, thanks for the reviewer for pointing out our mistake.

As shown in *Fig. 15 for reviewer*, the upper figure was the illustration of loop interactions and non-loop interactions, and the lower figure was used to show neck interactions at the same genomic regions as the upper figure. Four numbers were used to perform hypergeometric test: **N** indicates the number of the whole-genome interactions; **M** indicates the number of loop interactions; **n** indicates the number of all neck interactions, and **k** indicates the number of neck interactions which are also loop interactions.

N, M, n, k is 25917290, 19632, 865635, 1150, respectively. We calculated the probability that at least k neck interactions are also loop interactions, not exactly k neck interactions. We performed R cmd “phyper (k-1, M, N, n, lower.tail = FALSE) and got p-value = 4.30e-71. We have updated these details in the revised manuscript.


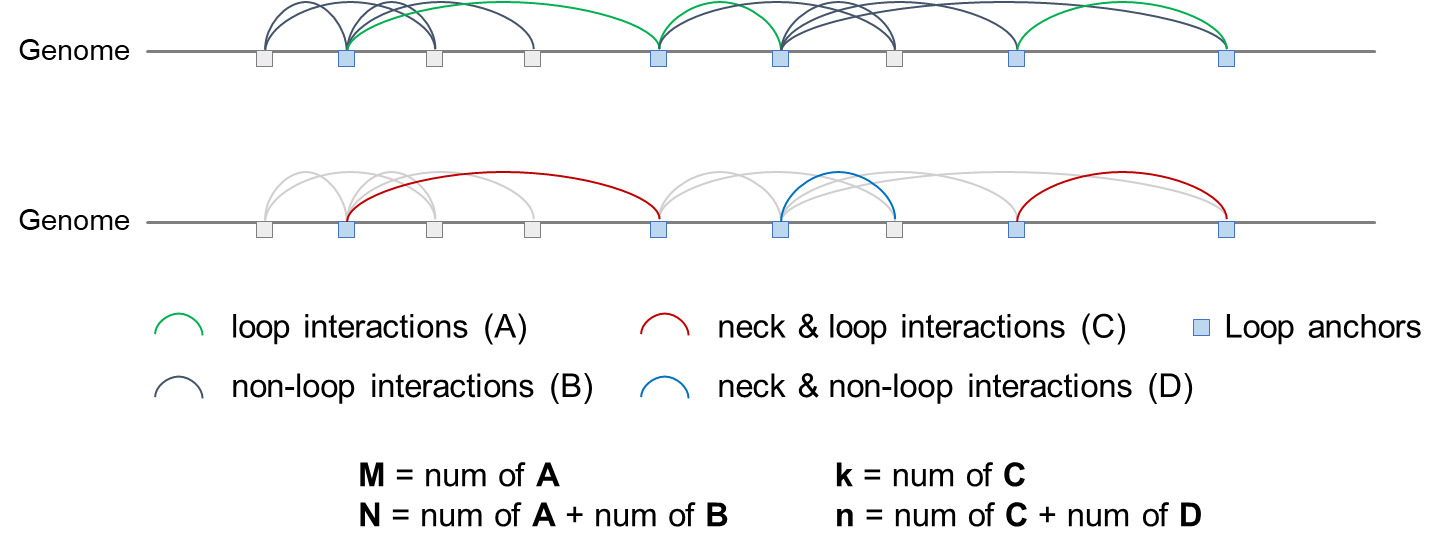


**Fig. 15 for reviewer.** Description of hypergeometric test calculation in our work.

*3. If a chi-square test is performed in the way described, how were the authors able to conclude that the neck neighbors are enriched in coding regions rather than depleted from them, since the chi-square test is only about dependency between two variables but not their direction of dependency?*

**Response**: The reviewer was right that chi-square test is only about dependency between two variables, and it is not enough to support our claim.

To address this, we first conducted the 2 x 2 matrix (*Table. 6 for reviewer*). Then, we calculated the Odds Ratio as OR = (36987/88874) / (121524/618249) = 2.1177. Finally, we performed chi-square test using R function “chisq.test” and got χ^2^ = 12077 as well as p-value < 2.2e-16. Likewise, for UTR, the 2 x 2 matrix is *Table. 7 for reviewer*. The OR = (10253/115608) / (20448/719325) = 3.1199 and we performed “chisq.test” again and got χ^2^ = 9106.8 as well as p-value < 2.2e-16. These results showed that “*Neck neighbours of DSB sites were enriched in coding regions CDS* *(Odds Ratio = 2.12,* *χ^2^ = 12077, p-value < 2.2e-16) as well as in UTRs (Odds Ratio = 3.12, χ^2^ = 9106.8, p-value < 2.2e-16)), compared with those of non-DSB sites (Fig. 4c)*”.

Thanks for pointing out our misuse of hypothesis testing and we have corrected this part in the revised manuscript.

**Table. 6 for reviewer**

|  | Neck neighbour location | |
| --- | --- | --- |
| prediction node type | CDS | not CDS |
| DSB | 36987 | 88874 |
| non-DSB | 121524 | 618249 |

**Table.7 for reviewer**

|  | Neck neighbour location | |
| --- | --- | --- |
| prediction node type | UTR | Not UTR |
| DSB | 10253 | 115608 |
| non-DSB | 20448 | 719325 |

*Why are UTRs considered "coding regions"?*

**Response**: Sorry for this mistake. We were meant to state that “neck neighbours of DSB sites were significantly enriched in coding regions CDS as well as in UTRs”. We have it corrected in the new revision.
